# A cell-free biosynthesis platform for modular construction of protein glycosylation pathways

**DOI:** 10.1038/s41467-019-12024-9

**Published:** 2019-11-27

**Authors:** Weston Kightlinger, Katherine E. Duncker, Ashvita Ramesh, Ariel H. Thames, Aravind Natarajan, Jessica C. Stark, Allen Yang, Liang Lin, Milan Mrksich, Matthew P. DeLisa, Michael C. Jewett

**Affiliations:** 1 Department of Chemical and Biological Engineering, Northwestern University, 2145 Sheridan Road, Tech E136, Evanston, IL 60208 USA; 2 Center for Synthetic Biology, Northwestern University, 2145 Sheridan Road, Tech B486, Evanston, IL 60208 USA; 3 Department of Biomedical Engineering, Northwestern University, 2145 Sheridan Road, Evanston, IL 60208 USA; 4 Medical Scientist Training Program, Feinberg School of Medicine, Northwestern University, Morton Building 1-670, 303 E. Chicago Avenue, Chicago, IL 60611 USA; 5 Department of Microbiology, Cornell University, 123 Wing Drive, Ithaca, NY 14853 USA; 6 Department of Chemistry, Northwestern University, Evanston, IL 60208 USA; 7 Robert Frederick Smith School of Chemical and Biomolecular Engineering, Cornell University, 120 Olin Hall, Ithaca, NY 14853 USA; 8 Nancy E. and Peter C. Meinig School of Biomedical Engineering, Cornell University, Weill Hall, Ithaca, NY 14853 USA

**Keywords:** Biological techniques, Biotechnology, Synthetic biology, Chemical biology, Glycobiology

## Abstract

Glycosylation plays important roles in cellular function and endows protein therapeutics with beneficial properties. However, constructing biosynthetic pathways to study and engineer precise glycan structures on proteins remains a bottleneck. Here, we report a modular, versatile cell-free platform for glycosylation pathway assembly by rapid in vitro mixing and expression (GlycoPRIME). In GlycoPRIME, glycosylation pathways are assembled by mixing-and-matching cell-free synthesized glycosyltransferases that can elaborate a glucose primer installed onto protein targets by an *N*-glycosyltransferase. We demonstrate GlycoPRIME by constructing 37 putative protein glycosylation pathways, creating 23 unique glycan motifs, 18 of which have not yet been synthesized on proteins. We use selected pathways to synthesize a protein vaccine candidate with an α-galactose adjuvant motif in a one-pot cell-free system and human antibody constant regions with minimal sialic acid motifs in glycoengineered *Escherichia coli*. We anticipate that these methods and pathways will facilitate glycoscience and make possible new glycoengineering applications.

## Abstract (web summary)

Constructing biosynthetic pathways to study and engineer glycoprotein structures is difficult. Here, the authors use GlycoPRIME, a cell-free workflow for mixing-and-matching glycosylation enzymes, to evaluate 37 putative glycosylation pathways and discover routes to 18 new glycoprotein structures

## Introduction

Protein glycosylation, the enzymatic process that attaches oligosaccharides to amino acid sidechains, is among the most abundant and complex post-translational modifications in nature^1,2^ and plays critical roles in human health^1^. Glycosylation is present in over 70% of protein therapeutics^3^ and profoundly affects protein stability^4,5^, immunogenicity^6,7^, and activity^8^. The importance of glycosylation in biology and evidence that intentional manipulation of glycan structures on proteins can improve therapeutic properties^4,6,8^ have motivated many efforts to study and engineer protein glycosylation structures^9–11^.

Unfortunately, glycoprotein engineering is constrained by the number and diversity of glycan structures that can be built on proteins and platforms available for glycoprotein production^9,12^. A key challenge is that glycans are synthesized in nature by many glycosyltransferases (GTs) across several subcellular compartments^1^, complicating engineering efforts and resulting in structural heterogeneity^3,12^. Furthermore, essential biosynthetic pathways in eukaryotic organisms limit the diversity of glycan structures that can be engineered in those systems^9,13^. Bacterial glycoengineering addresses these limitations by expressing heterologous glycosylation pathways in laboratory *Escherichia coli* strains that lack endogenous glycosylation enzymes^13,14^. Several asparagine (*N*-linked) glycosylation pathways have been successfully reconstituted in bacterial cells^13–17^ and cell-free systems^18–21^. In particular, cell-free systems, in which proteins and metabolites are synthesized in crude cell lysates, can accelerate the characterization and engineering of enzymes and biosynthetic pathways^22–25^. *E. coli*-based cell-free protein synthesis (CFPS) systems can produce gram per liter titers of complex proteins in hours^26^, enabling the rapid discovery, prototyping, and optimization of metabolic pathways without reengineering an organism for each pathway iteration^23–25^.

However, existing cell-free glycoprotein synthesis platforms have yet to fully exploit this paradigm because they rely on oligosaccharyltransferases (OSTs) to transfer prebuilt sugars from lipid-linked oligosaccharides (LLOs) onto proteins. OSTs are difficult to express because they are integral membrane proteins that often contain multiple subunits^1^. Furthermore, the LLO substrate specificities of OSTs limit modularity and the diversity of glycan structures that can be transferred to proteins^27^. Finally, LLOs competent for transfer by OSTs are difficult to synthesize in vitro^12^. In fact, it has not yet been shown that LLO biosynthesis and glycosylation can be co-activated in vitro or that LLOs can be both transferred and extended in a bacterial CFPS system. Instead, LLOs must be derived from or pre-enriched in cell lysates by expression of LLO biosynthesis pathways in living cells^18–20^. Expressing LLO biosynthesis pathways in cells requires time-consuming cloning and tuning of polycistronic operons, cellular transformation, and the production of new lysates for each glycan structure. Taken together, the complexity of membrane-associated OSTs and LLOs as well as OST substrate specificities present obstacles for glycoengineering and prevent the facile construction and screening of multienzyme glycosylation pathways^12^.

*N*-glycosyltransferases (NGTs) may overcome these limitations by enabling the construction of simplified, OST- and LLO-independent protein glycosylation pathways^9,16,28^. NGTs are cytoplasmic, bacterial enzymes that transfer a glucose residue from a uracil-diphosphate-glucose (UDP-Glc) sugar donor onto asparagine sidechains^29^. Importantly, NGTs are soluble enzymes that can install a glucose primer onto proteins in the *E. coli* cytoplasm^16,17,22^. This primer can then be sequentially elaborated by co-expressed GTs^16,28^. Synthetic NGT-based glycosylation systems are not limited by OST substrate specificities and do not require protein transport across membranes or lipid-associated components^9^. These systems have elicited great interest as a complementary approach for synthesis of glycoproteins, including therapeutics and vaccines, that are difficult or impossible to produce using OST-based systems^9,16,22,28,30–32^. Several recent advances set the stage for this vision. First, rigorous characterization of the acceptor specificity of NGTs using glycoproteomics and the GlycoSCORES technique^17,22,31^ have revealed that NGTs modify N-X-S/T amino acid motifs. Second, the NGT from *Actinobacillus pleuropneumoniae* (ApNGT) has been shown to modify native and rationally designed glycosylation sites within eukaryotic proteins in vitro and in *E. coli*^16,17,22,28^. Third, the Aebi group and others recently reported the elaboration of the glucose installed by ApNGT to polysialyllactose^28^ or dextran^16^ motifs in *E. coli* cells as well as a chemoenzymatic method to transfer prebuilt oxazoline-functionalized oligosaccharides onto this glucose residue^30,32^. However, other biosynthetic pathways to build glycans using NGTs have not been explored^9^, perhaps due to slow timelines associated with building and testing synthetic glycosylation pathways in living cells. A cell-free synthesis platform based on ApNGT would accelerate glycoengineering efforts by enabling high-throughput and entirely in vitro construction, assembly, and screening of synthetic glycosylation pathways.

Here, we describe a modular, cell-free method for glycosylation pathway assembly by rapid in vitro mixing and expression (GlycoPRIME). In this two-pot method, crude *E. coli* lysates are selectively enriched with individual GTs by CFPS expression and then combined in a mix-and-match fashion to construct multienzyme glycosylation pathways. The goal of GlycoPRIME is to design, build, test, and analyze many combinations of enzymes without making new genetic constructs, strains, cell lysates, or purified enzymes for each combination to discover new biosynthetic pathways (including many not found in nature) to glycoprotein structures of interest. These enzyme combinations can then be transferred to biomanufacturing systems, such as living cells, and used to produce and test glycoproteins. A key feature of GlycoPRIME is the use of ApNGT to site-specifically install a single *N*-linked glucose primer onto proteins, which can be elaborated to a diverse repertoire of glycans. The use of ApNGT as the initiating glycosylation enzyme removes constraints on glycan structure imposed by OST specificities for LLOs and enables the first entirely in vitro glycosylation (IVG) pathway synthesis and screening workflow by obviating the need to synthesize glycans on LLO precursors in living cells.

To validate GlycoPRIME, we optimize the in vitro expression of 24 bacterial and eukaryotic GTs and combine them to create 37 putative biosynthetic pathways to elaborate the glucose installed by ApNGT on a model glycoprotein substrate. We generated 23 unique glycan structures composed of 1 to 5 core saccharides and longer repeating structures. These pathways yielded 18 glycan structures that have not yet been reported on proteins and provide new biosynthetic routes to therapeutically relevant motifs including an α1-3-linked galactose (αGal) epitope as well as fucosylated and sialylated lactose or poly-*N*-acetyllactosamine (LacNAc). We then demonstrate that pathways identified using GlycoPRIME can be transferred to cell-free and cellular biosynthesis systems by producing (i) a protein vaccine candidate with an adjuvanting αGal glycan^6,7,33^ in a one-pot cell-free protein synthesis driven glycoprotein synthesis (CFPS-GpS) platform and (ii) the constant region (Fc) of the human immunoglobulin (IgG1) antibody in the *E. coli* cytoplasm with minimal sialic acid glycans known to improve in vivo pharmacokinetics^5,34^. The GlycoPRIME method represents a powerful approach to accelerate the construction and screening of multienzyme glycosylation pathways. By identifying feasible synthetic glycosylation pathways, we anticipate that GlycoPRIME will enable future efforts to produce and engineer glycoproteins for compelling applications including fundamental studies and improved therapeutics.

## Results

### Establishing an in vitro glycoengineering platform

**Fig. 1:**
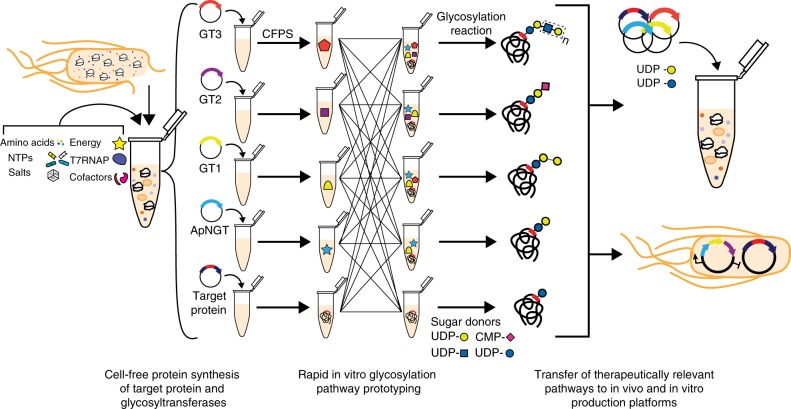
A platform for glycosylation pathway assembly by rapid in vitro mixing and expression (GlycoPRIME). GlycoPRIME was established to construct and screen biosynthetic pathways yielding diverse *N*-linked glycans. Crude *E. coli* lysates enriched with a target protein or individual glycosyltransferases (GTs) by cell-free protein synthesis (CFPS) were mixed in various combinations to identify biosynthetic pathways for the construction of various *N*-linked glycans. A model acceptor protein (Im7-6), the *N*-linked glycosyltranferase from *A. pleuropneumoniae* (ApNGT), and 23 elaborating GTs were produced in CFPS and then assembled with activated sugar donors to identify 23 biosynthetic pathways yielding unique glycosylation structures, several with therapeutic relevance. Pathways discovered in vitro were transferred to cell-free or cell-based production platforms to produce therapeutically relevant glycoproteins

We established GlycoPRIME as a modular, in vitro protein synthesis and glycosylation platform to develop biosynthetic pathways which elaborate the *N*-linked glucose priming residue installed by ApNGT to diverse glycosylation motifs including sialylated and fucosylated forms of lactose and LacNAc as well as an αGal epitope (Fig. 1).

**Fig. 2:**
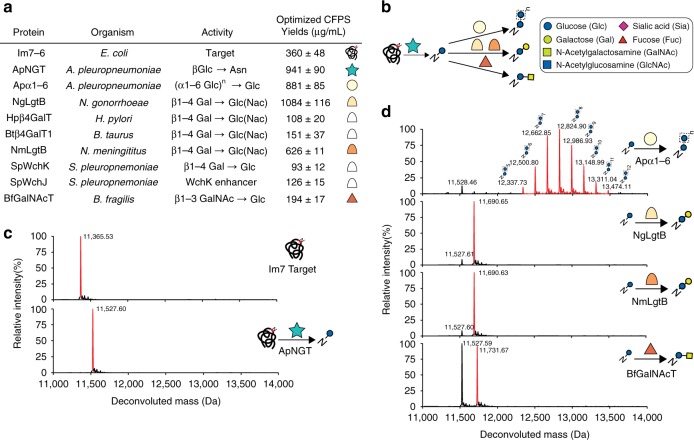
In vitro synthesis and assembly of one- and two-enzyme glycosylation pathways. **a** Protein name, species, previously characterized activity and optimized soluble CFPS yields for Im7-6 target protein, ApNGT, and GTs selected for glycan elaboration. References for previously characterized activities in Supplementary Table 4. CFPS yields indicate mean and standard deviation (s.d.) from *n* = 3 CFPS reactions quantified by [^14^C]-leucine incorporation. Full CFPS expression data in Supplementary Table 2 and Supplementary Figs. 1–2. **b** Symbol key and successful pathways for *N*-linked glucose installation on Im7-6 by ApNGT and elaboration by selected GTs. Glycan structures in this article use Symbol Nomenclature for Glycans (SNFG) and Oxford System conventions for linkages. Sialic acid refers to *N*-acetylneuraminic acid. **c** Deconvoluted mass spectrometry spectra from Im7-6 protein purified from IVG reactions assembled from CFPS reaction products with and without 0.4 µM ApNGT as well as 2.5 mM UDP-Glc. Full conversion to *N*-linked glucose was observed after 24 h at 30 °C. **d** Intact deconvoluted MS spectra from Im7 protein purified from IVG reactions containing 10 µM Im7-6, 0.4 µM ApNGT, and 7.8 µM NmLgtB, 13.9 µM NgLgtB, 3.1 µM BfGalNAcT, or 9.4 µM Apα1-6. IVG reactions were supplemented with 2.5 mM UDP-Glc as well as 2.5 mM UDP-Gal or 5 mM UDP-GalNAc as appropriate for 24 h at 30 °C. Observed mass shifts and MS/MS fragmentation spectra (Supplementary Fig. 3) are consistent with efficient modification of *N*-linked glucose with β1-4Gal, β1-4Gal, β1-3GalNAc, or α1-6 dextran polymer. Theoretical protein masses found in Supplementary Table 3. Hpβ4GalT, Btβ4GalT1, and SpWchJ + K did not modify the *N*-linked glucose installed by ApNGT (Supplementary Fig. 4). All spectra were acquired from full elution peak areas of all detected glycosylated and aglycosylated Im7-6 species and are representative of *n* = 3 independent IVGs. Spectra from m/z 100–2000 were deconvoluted into 11,000–14,000 Da using Bruker Compass Data Analysis maximum entropy method. Source data is available in the Source Data file

For proof of concept, we aimed to glycosylate a model protein with ApNGT in a setting that would enable further glycan elaboration in our GlycoPRIME workflow. Specifically, we identified CFPS conditions that provided high GT expression titers so that the minimum volume of GT-enriched lysate required for complete glycoprotein conversion could be added to each IVG reaction, leaving sufficient reaction volume and generating the substrate for further elaboration by mixing cell-free lysates. Based on our previous characterization of ApNGT acceptor sequence specificity^22^, we selected an engineered version of the *E. coli* immunity protein Im7 (Im7-6) bearing a single, optimized glycosylation sequence of GGNWTT at an internal loop as our model target protein (Supplementary Table 1 and Supplementary Note 1). We used [^14^C]-leucine incorporation to measure and optimize the CFPS reaction temperature for our engineered Im7-6 target and ApNGT (Supplementary Table 2 and Fig. 2a) and confirmed their full-length expression by SDS-PAGE autoradiogram (Supplementary Figs. 1–2). We found that 23 °C provided the most soluble product for these proteins, balancing greater overall protein production at higher temperatures and greater solubility at lower temperatures. We synthesized Im7-6 and ApNGT by CFPS and then mixed those reaction products together along with UDP-Glc in a 32-µl IVG reaction. We then purified the Im7-6 substrate using Ni-NTA functionalized magnetic beads and performed intact glycoprotein liquid chromatography mass spectrometry (LC-MS) (see “Methods”). We observed nearly complete conversion of 10 µM of Im7-6 substrate (11 µl) with just 0.4 µM ApNGT (1 µl) (Fig. 2c), as indicated by a mass shift of 162 Da (the mass of a glucose residue) in the deconvoluted protein mass spectra (theoretical masses shown in Supplementary Table 3). This shows that CFPS products can be directly assembled into IVG reactions to produce glycoprotein with remaining reaction volume for the addition of elaborating GTs.

Next, we identified 7 GTs with previously characterized specificities that could be useful in elaborating the glucose primer installed by ApNGT to relevant glycans (Fig. 2 and Supplementary Table 4). Previous works indicate that in *A. pleuropnemoniae*, the glucose installed by ApNGT is modified by the polymerizing Apα1-6 glucosyltransferase to form *N*-linked dextran^29^ and that this structure could be a useful vaccine antigen^16,35^. Recent work also showed that the β1-4 galactosyltransferase LgtB from *Neisseria meningitis* (NmLgtB) can modify an ApNGT-installed glucose in *E. coli*, forming *N*-linked lactose (Galβ1-4Glc-Asn)^28^. Here, we attempted to recapitulate these pathways in vitro and selected 5 additional enzymes with potentially useful activities (Fig. 2a). We chose the *N*-acetylgalactosamine (GalNAc) transferase from *Bacteroides fragilis* (BfGalNAcT) because the GalNAc residue it installs^36^ could serve as an elaboration point for *O*-linked glycan epitopes. We also chose several β1-4 galactosyltransferases from *Streptococcus pneumoniae* (SpWchK), *Neisseria gonorrhoeae* (NgLgtB), *Helicobacter pylori* (Hpβ4GalT), and *Bos taurus* (Btβ4GalT1) to determine the optimal biosynthetic route to *N*-linked lactose. This was important because lactose is a known substrate of many GTs that modify milk oligosaccharides and the termini of human *N*-linked glycans^1,37–40^, making it a critical reaction node for further glycan diversification.

Once identified, we optimized CFPS conditions and confirmed the soluble, full-length expression of these 7 GTs (Fig. 2, Supplementary Table 2, and Supplementary Figs. 1–2), as well as SpWchJ from *S. pneumoniae*, which is known to enhance the activity of SpWchK^41^. We then assembled IVG reactions by mixing CFPS products containing these GTs with Im7-6 and ApNGT CFPS products along with UDP-Glc and other appropriate sugar donors according to previously characterized activities (Fig. 2). We observed Im7-6 intact mass shifts and tandem MS (MS/MS) fragmentation spectra of trypsinized glycopeptides consistent with the known activities of NmLgtB and NgLgtB (β1-4 galactosyltransferases), BfGalNAcT (a β1-3 *N*-acetylgalactosyltransferase), and Apα1-6 (a polymerizing α1-6 glucosyltransferase) (Fig. 2, Supplementary Fig. 3, and Supplementary Table 5). We did not observe modification by Hpβ4GalT, SpWchK (even with SpWchJ), or Btβ4GalT1 (even with α-lactalbumin and conditions conducive to disulfide bond formation) (Supplementary Fig. 4). By testing IVGs with decreasing amounts of NmLgtB and NgLgtB, we found that 2 µM of NmLgtB provided nearly complete conversion to *N*-linked lactose whereas the same amount of NgLgtB was less efficient (Supplementary Fig. 5). These results show that multienzyme glycosylation pathways can be rapidly synthesized, combinatorially assembled, and evaluated in vitro. Using this approach, we found that ApNGT and NmLgtB provide an efficient in vitro route to *N*-linked lactose and discovered that ApNGT and BfGalNAcT can site-specifically install a GalNAc-terminated glycan.

### Modular construction of diverse glycosylation pathways

**Fig. 3:**
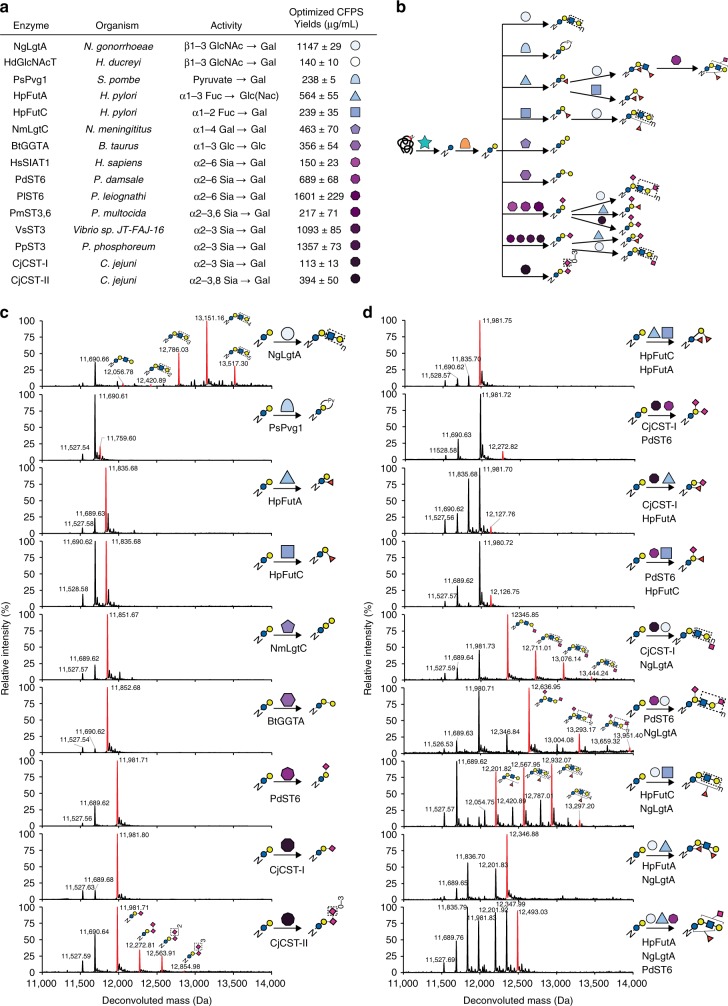
In vitro synthesis and assembly of complex glycosylation pathways. **a** Protein name, species, previously characterized specificity (Supplementary Table 4), and optimized CFPS soluble yields (Supplementary Table 2) for enzymes tested for elaboration of *N*-linked lactose. CFPS yields indicate mean and s.d. from *n* = 3 CFPS reactions quantified by [^14^C]-leucine incorporation. CjCST-I and HsSIAT1 yields were measured under oxidizing conditions (see Supplementary Fig. 9). (**b**) Intact deconvoluted MS spectra from Im7-6 protein purified from IVG reactions with 10 µM Im7-6, 0.4 µM ApNGT, 2 µM NmLgtB, and 2.5 mM appropriate nucleotide-activated sugar donors as well as 4.0 µM BtGGTA, 5.3 µM NmLgtC, 4.9 µM HpFutA, 2.6 µM HpFutC, 4.9 µM PdST6, 5.0 µM CjCST-II, 1.3 µM CjCST-I, 11.5 µM NgLgtA, or 2.2 µM SpPvg1. Mass shifts of intact Im7-6, fragmentation spectra of trypsinized Im7-6 glycopeptides (Supplementary Fig. 7), and exoglycosidase digestions (Supplementary Figs. 10-11) are consistent with modification of *N*-linked lactose with α1-3 Gal, α1-4 Gal, α1-3 Fuc, α2-6 Sia, α2-3 Sia, α2-8 Sia, β1-3 GlcNAc, or pyruvylation according to known activities of BtGGTA, NmLgtC, HpFutA, HpFutC, PdST6, CjCST-II, CjCST-I, NgLgtA, or SpPvg1. **d** Deconvoluted intact Im7-6 spectra of fucosylated and sialylated LacNAc structures produced by four- and five- enzyme combinations. IVG reactions contained 10 µM Im7-6, 0.4 µM ApNGT, 2 µM NmLgtB, appropriate sugar donors, and indicated GTs at half or one third the concentrations indicated in (**b**) for four- and five- enzyme pathways, respectively. Intact mass shifts and fragmentation spectra (Supplementary Fig. 12) are consistent with fucosylation and sialylation of the LacNAc core according to known activities. Intact protein and glycopeptide fragmentation spectra from other screened GTs and GT combinations not shown here are found in Supplementary Figs. 6–8 and 12–14. To provide maximum conversion, IVG reactions were incubated for 24 h at 30 °C, supplemented with an additional 2.5 mM sugar donors and incubated for another 24 h at 30 °C. Spectra were acquired from full elution areas of all detected glycosylated and aglycosylated Im7 species and are representative of *n* = 2 IVGs. Spectra from m/z 100–2000 were deconvoluted into 11,000–14,000 Da using Bruker Compass Data Analysis maximum entropy method. Source data is available in the Source Data file

To demonstrate the power of GlycoPRIME for modular pathway construction and screening, we next selected 15 GTs with known specificities that suggested their ability to elaborate the *N*-linked lactose installed by ApNGT and NmLgtB into a diverse repertoire of 3 to 5 saccharide motifs and longer repeating structures (Fig. 3 and Supplementary Table 4). Specifically, we sought to discover biosynthetic pathways that elaborate *N*-linked lactose to 9 oligosaccharides containing sialic acid (Sia), galactose (Gal), pyruvate, fucose (Fuc), and LacNAc. From there, we could obtain even greater diversity by recombining these GTs in various ways. We first describe our rationale for selecting these pathway classes, including their potential value for a variety of applications, and then present our experimental results.

Our first aim was to build glycans terminated in sialic acids because they provide many useful properties for applications in protein therapeutics^5,8,28,34,42^ (such as improved trafficking, stability, and pharmacodynamics); functional biomaterials^43^; binding interactions with bacterial receptors^44,45^, human galectins^46^, and siglecs^47^; as well as adjuvants^48^ and tumor-associated carbohydrate antigens (TACAs) for vaccines^49,50^. As the linkages of terminal sialic acids are important for these applications, we selected enzymes to install Sia with α2-3, α2-6, and α2-8 linkages onto the *N*-linked lactose. We began by building an *N*-linked 3′-sialyllactose (Siaα2-3Galβ1-4Glc-Asn) structure which could provide several useful properties including specific binding to pathogen receptors that adhere to human cells^44^, delivery of vaccines to macrophages for increased antigen presentation^48^, and mimicry of the human GM3 ganglioside (Siaα2-3Galβ1-4Glc-ceramide) for cancer vaccines^50^. The 3′-sialyllactose structure may also mimic the recently reported GlycoDelete structure (Siaα2-3Galβ1-4GlcNAc-Asn), a simplified *N*-glycan known to preserve glycoprotein therapeutic activity and pharmacokinetics^51^. To build 3′-sialyllactose, we chose four α2-3 sialyltransferases from *Pasteurella multocida* (PmST3,6), *Vibrio sp JT-FAJ-16* (VsST3), *Photobacterium phosphoreum* (PpST3), and *Campylobacter jejuni* (CjCST-I). Next, we aimed to discover biosynthetic routes to *N*-linked 6′-sialyllactose (Siaα2-6Galβ1-4Glc-Asn) because *N*-glycans bearing terminal α2-6Sia are common in secreted human proteins^5^, exhibit anti-inflammatory properties^8^, enable targeting of B cells for treatment of lymphoma^52^, and provide a distinct set of siglec, lectin, and receptor binding profiles^5,44,47^. To produce 6′-sialyllactose, we selected three α2-6 sialyltranferases from humans (HsSIAT1), *Photobacterium damselae* (PdST6), and *Photobacterium leiognathid* (PlST6). Finally, we investigated pathways to produce glycans with α2-8 Sia that may mimic the GD3 ganglioside (Siaα2-8Siaα2-3Galβ1-4Glc-ceramide), a TACA and possible vaccine epitope against melanoma^49,53^. Based on previous works^28,42^, we selected the CST-II bifunctional sialyltranferase from *C. jejuni* to install terminal α2-8 Sia. In addition to Sia-containing glycans, we explored the synthesis of pyruvalated galactose because this structure displays similar lectin-binding properties to Sia^54^. To build terminally pyruvylated lactose, we selected a pyruvyltransferase from *Schizosaccharomyces pombe* (SpPvg1)^54^.

Beyond structures terminated in Sia, we explored pathways to modify *N*-linked lactose with Gal, Fuc, and LacNAc. For example, we aimed to engineer a first-of-its-kind bacterial system for complete biosynthesis of proteins modified with *N*-linked αGal (Galα1-3Galβ1-4Glc-Asn) epitopes. αGal is an effective self:non-self discrimination epitope in humans and is bound by an estimated 1% of the human IgG pool^6,7,33^. Consequently, αGal confers adjuvant properties when associated with various peptide, protein, whole-cell, and nanoparticle-based immunogens^6,7,33,55^. To build αGal, we selected the α1,3 galactosyltransferase from *B. taurus* (BtGGTA). In addition, we sought to synthesize an *N*-linked globobiose structure (Galα1-4Galβ1-4Glc-Asn) because it may mimic the Gb3 ganglioside (Galα1-4Galβ1-4Glc-ceramide) which can bind and neutralize Shiga-like toxins secreted by pathogenic bacteria^56^. We selected the galactosyltransferase LgtC from *N. meningitis* (NmLgtC) to synthesize globobiose. We also aimed to build LacNAc because it provides useful properties for biomaterials^57^ as well as the inhibition and modulation of galectins to control cancer, inflammation, and fibrosis^58^. We selected two β1-3 *N*-acetylglucosamine (GlcNAc) transferases from *N. gonorrhoeae* (NgLgtA) and *Haemophilus ducreyi* (HdGlcNAcT) to make this structure. Finally, we aimed to build fucosylated lactose structures which may find applications in biomaterials for neuronal tissue^59^ as well as targeting or preventing the adherence of bacteria^60^. To synthesize fucosylated lactose, we screened α1,3 and α1,2 fucosyltransferases from *H. pylori* (HpFutA and HpFutC, respectively).

After designing pathways and selecting GTs, we used GlycoPRIME to synthesize and assemble three-enzyme biosynthetic pathways containing ApNGT, NmLgtB, and each of the 15 GTs described above. We first optimized and demonstrated full-length, soluble expression of each GT (Fig. 3a and Supplementary Table 2 and Supplementary Figs. 1–2). We then used the GlycoPRIME workflow to synthesize Im7-6, ApNGT, NmLgtB and GTs for glycan extension in separate CFPS reactions and then mixed these CFPS products and appropriate sugar donors to form IVG reactions. Remarkably, when IVG products were purified by Ni-NTA and analyzed by LC-MS(/MS), we observed intact Im7-6 mass shifts (Fig. 3 and Supplementary Fig. 6) and fragmentation spectra of trypsinized glycopeptides (Supplementary Fig. 7) consistent with the modification of the *N*-linked lactose installed by ApNGT and NmLgtB according to the hypothesized activities of all 15 GTs selected for elaboration of this structure except HdGlcNAcT (Supplementary Fig. 8). While we did detect some activity from all eight sialyltranferases by intact protein and/or glycopeptide analysis, we found that CjCST-I and PdST6 provided the highest conversion of all α2-3 and α2-6 sialyltranferases, respectively (Supplementary Fig. 6). This optimization demonstrates the ability of GlycoPRIME to quickly compare several biosynthetic pathways to determine the enzyme combinations that yield desired products. We also found that we could significantly increase the conversion of reactions containing CjCST-I and HsSIAT1 by conducting CFPS of those GTs in oxidizing conditions (Supplementary Fig. 9). This result demonstrates the advantages provided by the open reaction environment of CFPS reactions for improving enzyme synthesis, including the synthesis of a human enzyme with disulfide bonds (HsSIAT1). Notably, we found that NgLgtA not only installed GlcNAc, but also worked in turn with NmLgtB to form a LacNAc polymer with up to six repeat units (Fig. 3). In addition to intact protein and glycopeptide LC-MS(/MS), we performed digestions of Im7-6 modified by ApNGT, NmLgtB, and PdST6, HsSIAT1, CjCST-I, HpFutA, HpFutC, NgLgtA, and BtGGTA using commercially available exoglycosidases (Supplementary Figs. 10–11). Our findings support the previously established linkage specificities of these enzymes (Figs. 2 and 3 and Supplementary Table 4). Under these conditions, we found that PmST3,6 exhibited primarily α2-3 activity, which is consistent with previous reports^61^.

Having demonstrated the activity of diverse GTs using three-enzyme pathways, we pushed the GlycoPRIME system further to evaluate biosynthetic pathways containing four and five enzymes. Specifically, we aimed to synthesize sialylated and fucosylated lactose and LacNAc structures using combinations of HpFutA, HpFutC, CjCST-I, PdST6, and NgLgtA. Compared to the smaller glycans constructed above, these structures could provide greater specificity in a variety of applications including the targeting and inhibition of galectins, siglecs, and lectins on human and pathogenic cells^44,46,57,58^ as well as the adjuvanting of vaccines by installing Lewis-X glycan structures that bind DC-SIGN receptors on dendritic cells^62^. While some combinations of these GTs have been used to create free oligosaccharides or glycolipids^37–40,63–65^, the products resulting from interactions between their specificities have not been systematically studied in the context of a protein substrate. We used GlycoPRIME to test all pairwise combinations of these five GTs, expressing each of them in separate CFPS reactions and then mixing two of those crude lysates in equal volumes with CFPS reactions containing 10 µM Im7-6, 0.4 µM ApNGT, and 2 µM NmLgtB. In our analysis of these IVG products, we observed intact protein (Fig. 3d) and glycopeptide fragmentation products (Supplementary Fig. 12) indicating the synthesis of several interesting structures including difucosylated lactose, disialylated lactose, lactose variants with combinations of sialylation and fucosylation linkages, sialylated LacNAc structures with branching or only terminal Sia, and fucosylated LacNAc structures. Our analysis also revealed some possible specificity conflicts between the enzymes. For example, the combinations of CjCST-I with HpFutA and PdST6 with HpFutC yielded products which were both sialylated and fucosylated, but PdST6 with HpFutC and CjCST-I with HpFutC did not (Supplementary Fig. 13). Furthermore, we observed that when HpFutC and NgLgtA are used together, only one fucose is added to the LacNAc backbone regardless of its length (Fig. 3d and Supplementary Fig. 12). In contrast, when HpFutA and NgLgtA are combined, our observations suggest that both available Glc(NAc) residues may be modified; however, the shorter polymer length suggests that fucosylation with HpFutA may prohibit the continued growth of the LacNAc chain by NgLgtA (Fig. 3). While we focused here on testing reactions with all pathway enzymes acting simultaneously, sequential glycosylation reactions in vitro using a similar workflow could be used to further characterize these specificity conflicts and rigorously determine enzyme kinetics. To test the number of biosynthetic nodes GlycoPRIME can support, we constructed several five-enzyme glycosylation pathways using NgLgtA, one fucosyltransferase (HpFutA or HpFutC), and one sialyltransferase (CjCST-I or PdST6). While the complexity of these glycans did not allow us to unambiguously assign their structures, the intact protein mass shifts (Supplementary Fig. 13) and fragmentation spectra (Supplementary Fig. 12) from pathways containing NgLgtA, PdST6, and either HpFutA or HpFutC indicated the construction of LacNAc structures glycans which were both fucosylated and sialylated (Fig. 3d and Supplementary Figs. 12 and 14). Many glycans synthesized by these four- and five-enzyme combinations have not been previously described and further study will be required to understand the functional properties they provide.

### GlycoPRIME pathways function in bacterial production systems

**Fig. 4:**
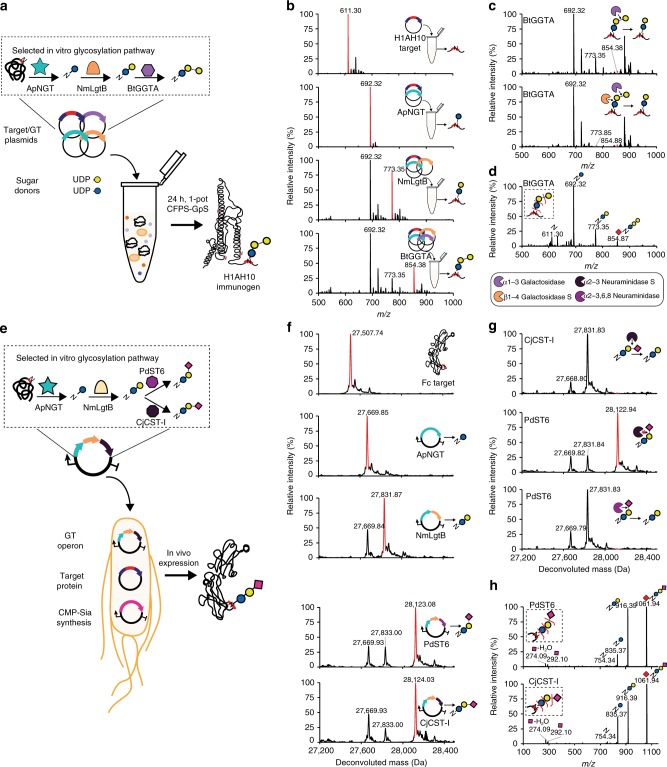
Design of biosynthetic pathways for cell-free and bacterial production platforms. **a** One-pot CFPS-GpS for synthesis of H1HA10 protein vaccine modified with αGal glycan. Plasmids encoding the target protein and biosynthetic pathway GTs discovered by GlycoPRIME screening were combined with appropriate activated sugar donors in a CFPS-GpS reaction. **b** Trypsinized glycopeptide MS spectra, **c** exoglycosidase digestions of glycopeptide, and **d** MS/MS glycopeptide fragmentation spectra from H1HA10 purified from IVG reactions containing equimolar amounts of each indicated plasmid encoding H1HA10, ApNGT, NmLgtB, and BtGGTA and 2.5 mM of UDP-Glc and UDP-Gal (see “Methods”). All reactions contained 10 nM total plasmid concentration and were incubated for 24 h at 30 °C. The glycopeptide contains one engineered acceptor sequence located at the *N*-terminus of H1HA10. Observed masses and mass shifts in (**b**–**d**) spectra are consistent with modification of the H1HA10 peptide with *N*-linked Glc by ApNGT, lactose (Galβ1-4Glc-Asn) by ApNGT and NmLgtB, or αGal epitope (Galα1-3Galβ1-4Glc-Asn) by ApNGT, NmLgtB, and BtGGTA. **e** Design of cytoplasmic glycosylation systems to produce sialylated IgG Fc in *E. coli*. Three plasmids containing NmNeuA (CMP-Sia synthesis), IgG Fc engineered with an optimized acceptor sequence (target protein), and biosynthetic pathways discovered using GlycoPRIME (GT operon). **f** Deconvoluted intact glycoprotein MS spectra, **g** exoglycosidase digestions of intact glycoprotein, and **h** MS/MS glycopeptide fragmentation spectra from Fc-6 purified from *E. coli* cultures supplemented with sialic acid, IPTG, and arabinose and incubated at 25 °C overnight (see “Methods”). The last GT in all glycosylation pathways is indicated. MS spectra were acquired from full elution areas of all detected glycosylated and aglycosylated protein or peptide species and are representative of *n* = 3 CFPS-GpS or *E. coli* cultures. MS/MS spectra acquired by pseudo multiple reaction monitoring (MRM) fragmentation at theoretical glycopeptide masses (red diamonds) corresponding to detected intact glycopeptide or protein MS peaks using 30 eV collisional energy. Deconvoluted spectra collected from *m/z* 100–2000 into 27,000–29,000 Da using Compass Data Analysis maximum entropy method. See Supplementary Tables 5–7 for theoretical masses

Having constructed and screened many new biosynthetic pathways using GlycoPRIME, we sought to demonstrate that the synthetic glycosylation pathways we discovered could be translated to new contexts within in vitro and in vivo bioproduction platforms to synthesize therapeutically relevant glycoproteins (Fig. 4).

First, we aimed to translate the glycosylation pathways discovered using our two-pot GlycoPRIME system to a one-pot, coordinated cell-free protein synthesis driven glycoprotein synthesis (CFPS-GpS) platform. In CFPS-GpS, the target protein is co-expressed with GTs in the presence of sugar donors to simultaneously synthesize and glycosylate the glycoprotein of interest. This strategy provides an alternative and complementary approach to our previously reported one-pot cell-free glycoprotein synthesis (CFGpS) platform^18^ by enabling expression of the glycosylation pathway enzymes in vitro rather than in vivo within the chassis strain before cell lysis. We validated our one-pot CFPS-GpS approach by mixing the Im7-6 target protein plasmid, sets of up to three GT plasmids based on 12 successful biosynthetic pathways developed in our two-pot GlycoPRIME screening, and appropriate sugar donors in one-pot CFPS-GpS reactions. In all reactions, we observed intact protein mass shifts consistent with the modification of Im7-6 with the same glycans observed in our two-pot system, albeit with lower efficiencies (Supplementary Fig. 15). These results show that co-activation of target protein and GT synthesis with protein glycosylation is possible in one-pot, in vitro reactions, further simplifying and shortening the time required to produce glycoproteins compared to the two-pot GlycoPRIME format. Overall, CFPS-GpS uses only plasmids, commercially available small molecules, and an unenriched crude *E. coli* lysate to yield glycoprotein, enabling the versatile production of different glycoprotein targets and/or glycan structures according to the need or desired application by simply adding different plasmids to a single crude lysate source.

Having developed the CFPS-GpS approach, we aimed to synthesize and glycosylate an influenza vaccine candidate, H1HA10^66^, with an αGal glycan motif using the biosynthetic pathway we discovered using GlycoPRIME (Fig. 4). We chose to demonstrate the αGal pathway on the H1HA10 model protein because H1HA10 is an effective immunogen that can be expressed in *E. coli* and the chemoenzymatic installation of αGal has been shown to act as an effective intramolecular adjuvant for other influenza vaccine candidates^7,67^. When we combined UDP-Glc, UDP-Gal, and plasmids encoding the H1HA10 protein ApNGT, NmLgtB, and BtGGTA in a one-pot CFPS-GpS reaction, we observed the installation of αGal on a tryptic peptide containing an engineered acceptor sequence at the *N*-terminus of H1HA10 (Fig. 4b). We further confirmed the linkages of this αGal glycan by exoglycosidase digestion and LC-MS/MS (Fig. 4c, d and Supplementary Table 6).

To demonstrate the transfer of pathways discovered using GlycoPRIME to living cells, we designed synthetic glycosylation systems to install *N*-linked 3′-sialyllactose and 6’-siallylactose onto the Fc region of human IgG1 in *E. coli* (Fig. 4). While glycoproteins with α2-8-linked polysialic acids have been produced in engineered *E. coli*^28^, these glycans with distinct terminal sialic acid linkages and simplified, more homogeneous structures can provide unique and desirable properties for some applications of glycoprotein therapeutics^5,8,34,51^. To this end, we constructed a three-plasmid system composed of a constitutively expressed cytidine-5′-monophospho-N-acetylneuraminic acid (CMP-Sia) synthesis plasmid encoding the *N. meningititus* CMP-Sia synthase (ConNeuA); an Isopropyl β-D-1-thiogalactopyranoside (IPTG)-inducible target protein plasmid; and a GT operon plasmid encoding ApNGT, NmLgtB, and either CjCST-I or PdST6. The CMP-Sia synthesis plasmid is necessary because laboratory *E. coli* strains do not endogenously produce CMP-Sia. Based on previous reports^28,40^, we selected a K-12 *E. coli* strain carrying the *nanT* sialic acid transporter gene for intake of Sia supplemented to the media and knocked out the CMP-Sia aldolase gene (*nanA*) to prevent digestion of intracellular Sia, yielding CLM24Δ*nanA*. As with CFPS-GpS, we validated the in vivo synthesis of our target glycans using the Im7-6 model protein. When we transformed and induced our three-plasmid system in CLM24Δ*nanA*, we observed intact protein spectra consistent with the modification of Im7-6 with *N*-linked Glc by ApNGT, elaboration to lactose by NmLgtB, and elaboration to 3′-sialyllactose or 6′-siallylactose by CjCST-I or PdST6, respectively (Supplementary Fig. 16). To synthesize Fc modified with these glycans, we replaced the Im7-6 target plasmid with a plasmid encoding Fc with an engineered acceptor sequence at the conserved human IgG1 glycosylation site at Asn297 (Fc-6)^22^. In this system, we observed intact protein MS, MS/MS peptide fragmentation, and exoglycosidase digestions consistent with the expected installation of Glc, lactose, and either 3′-sialyllactose or 6′-sialyllactose onto Fc-6 according to the GT operon supplied (Fig. 4f–h, Supplementary Fig. 17, and Supplementary Table 7). Further investigations will be required to assess the efficacy of the αGal epitope as an adjuvant for H1HA10 and the therapeutic effects of minimal sialic acid motifs on Fc. However, our findings clearly demonstrate that useful glycosylation pathways identified in the GlycoPRIME workflow can be quickly and easily translated to bacterial cell-free and cell-based expression platforms for the production of therapeutically relevant glycoproteins.

## Discussion

This work establishes and demonstrates the utility of the GlycoPRIME platform, a cell-free workflow for the modular synthesis, assembly, and discovery of multienzyme glycosylation pathways. GlycoPRIME has several key features. First, by removing the need for LLO production in living cells, GlycoPRIME is the first system to enable the biosynthesis of a glycosylation target, GTs, and glycoproteins entirely in vitro. This approach shifts the design-build test unit from a living cell line to a cell-free lysate. We demonstrated the utility of GlycoPRIME by rapidly exploring 37 putative protein glycosylation pathways, 23 of which yielded unique glycosylation motifs.

Second, the use of ApNGT (a soluble, bacterial enzyme) to efficiently install a priming *N*-linked glucose onto glycoproteins was key to facilitating pathway assembly. By elaborating this glucose residue, we generated a diverse library of therapeutically relevant glycosylation motifs from the bottom-up in vitro. Of the 23 unique glycosylation motifs for which biosynthetic pathways were discovered in this work, several have been synthesized as free^37–40,63,64^ or lipid-linked^37,38^ oligosaccharides or by remodeling existing glycoproteins^6,30,42^; however, to our knowledge, only glucose^16,22,28^, dextran^16^, lactose^28^, LacNAc^65^, and polysialyllactose^28^ have been previously produced as glycoprotein conjugates in bacterial systems. The 18 synthetic glycosylation pathways leading to novel glycan motifs on proteins discovered in this work represent the largest addition made by any single bacterial glycoengineering study to date. Specifically, we developed the first bacterial biosynthesis pathways that yield proteins bearing *N*-linked 3′-siallylactose, 6′-siallylactose, the αGal epitope, pyruvylated lactose, 2′-fucosyllactose (Fucα1-2Galβ1-4Glc-Asn), 3-fucosyllactose (Galβ1-4[Fucα1-3]Glc-Asn), as well as many other mono- or di- fucosylated and sialylated forms of lactose or LacNAc.

Third, biosynthetic pathways identified in GlycoPRIME can be implemented in new contexts and on new proteins for glycoprotein production in vitro and in *E. coli*. Specifically, we demonstrated the synthesis of a candidate vaccine protein, H1HA10, modified with an αGal adjuvant motif in a one-pot CFPS-GpS reaction and the production of IgG1 Fc modified with 3′-siallylactose and 6′-siallylactose in *E. coli* (Fig. 4). While large-scale production and purification methods were not investigated, our work shows feasibility for translating pathways discovered by GlycoPRIME into relevant biomanufacturing expression systems. Furthermore, the use of ApNGT rather than OSTs makes these pathways attractive because they do not require transport across cellular membranes or membrane-associated components. These findings demonstrate the potential of GlycoPRIME to accelerate glycoengineering efforts and enable new applications in biotechnology, including on-demand production of glycoprotein therapeutics in combination with recent developments in distributed biomanufacturing systems^21,68,69^ and *E. coli* strains with reduced endotoxin levels^21,70,71^.

While the glycosylation structures created in this work are less complex than natural human glycans, they still offer many promising applications. Potential applications include the development of imaging and other research reagents for fundamental studies of carbohydrate-binding proteins^44^; glycan-based bacterial targeting^60^, toxin neutralization^56^, and adhesion prevention^44,45,60^; improvement of glycoprotein therapeutic properties and trafficking^5,8,28,34,42,52^; new opportunities in functional biomaterials^43,57,59^; modulation and inhibition of human galectins^46^ and siglecs^46,47^; and the development of new antigens^49,50,53^ and adjuvants for immunization^6,7,33,48,55,62^. Although free oligosaccharides or small molecules can accomplish some of the functions above, the ability to build glycans site-specifically on glycoproteins as demonstrated in this work would enable a wide array of additional functionalities including targeting, antigen presentation, detection, imaging, and destruction^6,62^. Notably, further study will be required to assess the immunogenicity of the Glcβ-Asn linkage created by ApNGT whose presence has only once been reported in mammalian systems^72^. If this linkage is immunogenic, the glycoprotein structures described here could still have significant impact in research, acute therapeutic applications, or immunization. Additionally, recent works have aimed to discover or engineer NGTs with relaxed sugar donor specificities (such as GlcNAc)^32,73^ or combined these NGT variants with an acetyltransferase to produce *N*-linked GlcNAc^32^. We expect that these methods and future advancements will be compatible with most of the biosynthetic pathways described here because NmLgtB can modify Glc or GlcNAc acceptors^39^.

Looking forward, GlycoPRIME provides a new way to discover, study, and optimize glycosylation pathways. For example, future applications could leverage the open and flexible reaction environment of GlycoPRIME to optimize enzyme stoichiometry for more homogeneous biosynthesis and to better understand GT specificities and kinetics. By enabling the synthesis and rapid assembly of enzymes that yield desired glycoproteins, GlycoPRIME is also poised to further expand the glycoengineering toolkit towards the production of glycoproteins on demand and by design. For example, recently reported methods to supplement lipid-associated glycans into cell-free synthesis reactions^18–20^ or produce GalNAcTs^22^ and OSTs^19^ in vitro present new opportunities to discover biosynthetic pathways yielding diverse glycans (*N*- and *O*-linked) with small modifications to the GlycoPRIME workflow. Finally, the diverse, yet simple set of glycans accessible by GlycoPRIME pathways could help elucidate the minimal motifs that provide desired glycoprotein properties. In sum, we expect that GlycoPRIME and biosynthetic pathways described in this work will accelerate the engineering of glycoproteins in bacterial systems, helping to merge the glycoscience and synthetic biology communities.

## Methods

### Plasmid construction and molecular cloning

Details and sources of plasmids used in this study are shown in Supplementary Table 1 with applicable database accession numbers. Full coding sequence regions with plasmid context are shown in Supplementary Note 1. Codon-optimized DNA sequences encoding glycosylation targets and GTs in CFPS were synthesized as gene fragments or intact plasmids by Twist Bioscience, Integrated DNA Technologies, or Life Technologies. Gene fragments were inserted between NdeI and SalI restriction sites in the Kanamycin-resistant pJL1^22^ in vitro expression vector using polymerase chain reaction (PCR) amplification and Gibson assembly according to standard molecular biology techniques^74^. Some GTs were produced with an *N*-terminal CAT-Strep-Linker (CSL) fusion sequence that has been shown to increase in vitro expression^22^ (see Supplementary Note 1). Plasmids for expression of Im7-6 and Fc-6 glycosylation targets in the CLM24∆*nanA E. coli* strain were generated by PCR amplification of engineered forms of Im7 (Im7-6) and Fc (Fc-6) carrying optimized ApNGT glycosylation acceptor sequences and His-tags from pJL1.Im7-6 and pJL1.Fc-6^22^. These gene fragments were then placed into a pBR322 (ptrc99) backbone^75^ with Carbenicillin resistance and IPTG inducible expression between NcoI and HindIII restriction sites using Gibson assembly. Plasmids for expression of GT operons in *E. coli* were constructed by PCR amplification of ApNGT, NmLgtB, and CjCST-I or PdST6 from their pJL1 plasmid forms followed by Gibson assembly into a pMAF10 backbone^22^ with Trimethoprim resistance, a pBBR1 origin of replication, and arabinose inducible expression between NcoI and HindIII restriction sites. Strep-II tags, FLAG-tags, and ribosome binding sites designed using the RBS Calculator v2.0^76^ for maximum translation initiation rate were inserted into these plasmids as shown in Supplementary Table 1 and Supplementary Note 1. The pCon.NeuA plasmid for production of CMP-Sia in *E. coli* was generated by PCR amplification of NeuA from pTF^77^ followed by Gibson assembly into a pConYCG backbone with Kanamycin resistance and modified with a P_32100_ promoter for constitutive expression between the NsiI and SalI restriction sites.

### Preparation of cell extracts for CFPS

CFPS of glycosylation enzymes and target proteins was performed using crude *E. coli* lysate from a recently described, high-yielding MG1655-derived *E. coli* strain C321.∆A.759^26^ prepared using well-established methods^22,26^. Briefly, 1-liter cultures of *E. coli* cells were grown from a starting OD_600_ = 0.08 in 2xYTPG media (yeast extract 10 g/l, tryptone 16 g/l, NaCl 5 g/l, K_2_HPO_4_ 7 g/l, KH_2_PO_4_ 3 g/l, and glucose 18 g/l, pH 7.2) in 2.5-liter Tunair flasks at 34 °C with shaking at 250 r.p.m. Cells were harvested on ice at OD_600_ = 3.0 and pelleted by centrifugation at 5000 × *g* at 4 °C for 15 min. Cell pellets were washed three times with cold S30 buffer (10 mM Tris-acetate pH 8.2, 14 mM magnesium acetate, 60 mM potassium acetate, 2 mM dithiothreitol [DTT]) before being frozen on liquid nitrogen and then stored at −80 °C. Cell pellets were thawed on ice and resuspended in 0.8 ml of S30 buffer per gram of wet cell weight and lysed in 1.4 ml aliquots on ice using a Q125 Sonicator (Qsonica) using three pulses (50% amplitude, 45 s on and 59 s off). After sonication, 4 µl of 1 M DTT was added to each aliquot. Each aliquot was centrifuged at 12,000 × *g* and 4 °C for 10 min. The supernatant was incubated at 37 °C at 250 r.p.m. for 1 h and centrifuged at 10,000 × *g* at 4 °C for 10 min. The clarified S12 lysate supernatant was then frozen on liquid nitrogen and stored at −80 °C.

### Cell-free protein synthesis

CFPS of glycosylation targets and GTs was performed using a well-established PANOx-SP crude lysate system^26^. Briefly, CFPS reactions contained 0.85 mM each of GTP, UTP, and CTP; 1.2 mM ATP; 170 µg/ml of *E. coli* tRNA mixture; 34 µg/ml folinic acid; 16 µg/ml purified T7 RNA polymerase; 2 mM of each of the 20 standard amino acids; 0.27 mM coenzyme-A (CoA); 0.33 mM nicotinamide adenine dinucleotide (NAD); 1.5 mM spermidine; 1 mM putrescine; 4 mM sodium oxalate; 130 mM potassium glutamate; 12 mM magnesium glutamate; 10 mM ammonium glutamate; 57 mM HEPES at pH = 7.2; 33 mM phosphoenolpyruvate (PEP); 13.3 µg/ml DNA plasmid template encoding the desired protein in the pJL1 vector; and 27% v/v of *E. coli* crude lysate. *E. coli* total tRNA mixture (from strain MRE600) and PEP were purchased from Roche Applied Science. ATP, GTP, CTP, UTP, the 20 amino acids, and other materials were purchased from Sigma-Aldrich. Plasmid DNA for CFPS was purified from DH5-α *E. coli* strain (NEB) using ZymoPURE Midi Kit (Zymo Research). CFPS reactions under oxidizing conditions conducive to disulfide bond formation were performed similarly to standard CFPS reactions except for the use of a 30 min preincubation of the lysate with 14.3 µM IAM and the addition of 4 mM oxidized L-glutathione GSSG, 1 mM reduced L-glutathione, and 3 µM of purified *E. coli* DsbC to the CFPS reaction^78^. All proteins were expressed in 15 µl batch CFPS reactions in 2.0 ml centrifuge tubes. For GlycoPRIME, CFPS reactions were incubated for 20 h at optimized temperatures for each protein (Supplementary Table 2).

### Cell-free protein synthesis driven glycoprotein synthesis

One-pot, CFPS-GpS was performed similarly to CFPS, except that CFPS-GpS reactions had a total volume of 50 µl and were supplemented with 2.5 mM of each appropriate activated sugar donor as well as multiple plasmid templates from the desired target protein and up to three GTs. CFPS-GpS reactions contained a total plasmid concentration of 10 nM, divided equally between each of the unique plasmids in the reaction. CFPS-GpS reactions were incubated for 24 h at 23 °C before purification by Ni-NTA magnetic beads for glycopeptide or intact protein analysis by LC-MS.

### Quantification of CFPS yields

CFPS yields of glycosylation targets and GTs for GlycoPRIME were determined by supplementation of standard CFPS reactions with 10 µM [^14^C]-leucine using established protocols^22,26^. Briefly, proteins produced in CFPS were precipitated and washed three times using 5% trichloroacetic acid (TCA) followed by quantification of incorporated radioactivity by a Microbeta2 liquid scintillation counter. Soluble yields were determined from fractions isolated after centrifugation at 12,000 × *g* for 15 min at 4 °C. Low levels of background radioactivity were measured in CFPS reactions containing no plasmid template and subtracted before calculation of protein yields.

### Autoradiograms of CFPS reaction products

Autoradiograms of the soluble fractions of Im7-6 target and enzymes used in GlycoPRIME according to established methods^22^. Briefly, 2 µl CFPS reactions supplemented with 10 µM [^14^C]-leucine prior to the CFPS reaction and centrifuged at 12,000 × *g* for 15 min at 4 °C after the CFPS reaction were separated using a 4–12% Bolt Bis-Tris Plus SDS-PAGE gel (Invitrogen) using MOPS buffer. The gels were stained using InstantBlue (Expedeon), imaged, and then dried overnight between cellophane films before a 72 h exposure to a Storage Phosphor Screen (GE Healthcare). The Phosphor Screen was imaged using a Typhoon FLA7000 imager (GE Healthcare) and the dried gels were imaged using a GelDoc XR + Imager (Bio-Rad) to assist with alignment to molecular weight standard ladder. SDS-PAGE and autoradiogram gel images were acquired using Image Lab Software version 6.0.0 and Typhoon FLA 7000 Control Software Version 1.2 Build 1.2.1.93, respectively.

### IVG reactions

IVG reactions for GlycoPRIME were assembled in standard 0.2 ml tubes from the supernatant of completed CFPS reactions containing the Im7-6 target protein and indicated GTs centrifuged at 12,000 × *g* for 10 min at 4 °C. Target and enzyme yields were quantified and optimized by [^14^C]-leucine incorporation (Supplementary Table 2). Standard IVG reactions contained 10 µM Im7-6 target, indicated amounts of up to five GTs forming a putative biosynthetic pathway, 10 mM MnCl_2_ (to provide the preferred metal cofactor for NmLgtB and other GTs), 23 mM HEPES buffer at pH = 7.5, and 2.5 mM of each required nucleotide-activated sugar donor (according to previously characterized activities shown in Supplementary Table 4). Each reaction contained a total volume of 32 µl with 25 µl of completed CFPS reactions (when necessary, the remaining CFPS reaction volume was filled by a completed CFPS reaction which had synthesized sfGFP). After assembly, IVG reactions containing up to two GTs were incubated for 24 h at 30 °C. To increase conversion, IVG reactions containing more than two GTs were incubated for 24 h at 30 °C, supplemented with an additional 2.5 mM of each activated sugar donor, and then incubated for an additional 24 h. When desired, both CFPS reactions and IVGs could be flash-frozen after their respective incubation steps. After incubation, Im7-6 was purified from IVG reactions using magnetic His-tag Dynabeads (Thermo Fisher Scientific). The IVG reactions were diluted in 90 µl of Buffer 1 (50 mM NaH_2_PO_4_ and 300 mM NaCl, pH 8.0) and centrifuged at 12,000 × *g* for 10 min at 4 °C. This supernatant was incubated at room temperature for 10 min on a roller with 20 µl of beads which had been equilibrated with 120 µl of Buffer 1. The beads were then washed three times with 120 µl of Buffer 1 and then eluted using 70 µl of Buffer 1 with 500 mM imidazole. The samples were dialyzed against Buffer 2 (12.5 mM NaH_2_PO_4_ and 75 mM NaCl, pH 7.5) overnight using 3.5 kDa MWCO microdialysis cassettes (Pierce). Purification of one-pot CFPS-GpS reactions was completed similarly to IVG reactions.

### Production of glycoproteins from living *E. coli*

The *E. coli* strain CLM24∆*nanA* (genotype W3110 *ΔwecA ΔnanA ΔwaaL*::kan) was constructed to enable the intake and survival of sialic acid in the cytoplasm for the production of sialylated glycoproteins in vivo. CLM24*∆nanA* was generated from W3110 using P1 transduction of the *wecA*::*kan*, *nanA*::*kan*, and *waaL*::*kan* alleles in that order, derived from the Keio collection^79^. Between successive transductions, the kanamycin marker was removed using pE-FLP^80^. As indicated, CLM24∆*nanA* was sequentially transformed with the CMP-Sia production plasmid pCon.NeuA; a target protein plasmid pBR322.Im7-6 or pBR322.Fc-6; and a GT operon plasmid pMAF10.NGT, pMAF10.ApNGT.NmLgtB, pMAF10.CjCST-I.NmLgtB.ApNGT, or pMAF10.PdST6.NmLgtB.ApNGT by isolating individual clones with appropriate antibotics at each step. The completed strain was then used to inoculate a 5 ml overnight culture in LB media containing appropriate antibiotics which was then subcultured at OD_600_ = 0.08 into 5 ml of fresh LB media supplemented with 5 mM *N*-Acetylneuraminic acid (sialic acid) purchased from Carbosynth and adjusted to pH = 6.0 using NaOH and HCl. This culture was then grown at 37 °C with shaking at 250 r.p.m. GT operon expression was induced by supplementing the culture with 0.2% arabinose at OD_600_ = 0.4 and then target protein expression was induced at OD_600_ = 1.0 with 1 mM IPTG. After IPTG induction, the culture was grown overnight at 28 °C and 250 r.p.m. The cells were pelleted by centrifugation at 4 °C for 10 min at 4000 × *g*, frozen on liquid nitrogen, and stored at −80 °C. Cell pellets were thawed and resuspended in 630 μl of Buffer 1 with 5 mM imidazole and supplemented with 70 μl of 10 mg/ml lysozyme (Sigma), 1 μl (250 U) Benzonase (Millipore), and 7 µl of 100X Halt protease inhibitor (Thermo Fisher Scientific). After 15 min of thawing and resuspension, the cells were incubated for 15–60 min on ice, sonicated for 45 s at 50% amplitude, and then centrifuged at 12,000 × *g* for 15 min. The supernatant was then incubated on a roller for 10 min at RT with 50 µl of His-tag Dynabeads which had been pre-equilibrated with 5 mM imidazole in Buffer 1. The beads were then washed three times with 1 ml of Buffer 1 containing 5 mM imidazole and then eluted with 70 μl of Buffer 1 with 500 mM imidazole by a 10 min incubation on a roller at RT. Samples were then dialyzed with 3.5 kDa MWCO microdialysis cassettes overnight against Buffer 2 before glycopeptide or glycoprotein processing and analysis by LC-MS(/MS).

### LC–MS analysis of glycoprotein modification

Modification of intact glycoprotein targets was determined by LC-MS by injection of 5 µl (or about 5 pmol) of His-tag purified, dialyzed glycoprotein into a Bruker Elute UPLC equipped with an ACQUITY UPLC Peptide BEH C4 Column, 300 Å, 1.7 µm, 2.1 mm × 50 mm (186004495 Waters Corp.) with a 10 mm guard column of identical packing (186004495 Waters Corp.) coupled to an Impact-II UHR TOF Mass Spectrometer (Bruker Daltonics, Inc.). Before injection, Fc samples were reduced with 50 mM DTT. Liquid chromatography was performed using 100% H_2_O and 0.1% formic acid as Solvent A and 100% acetonitrile and 0.1% formic acid as Solvent B at a flow rate of 0.5 mL/min and a 50 °C column temperature. An initial condition of 20% B was held for 1 min before elution of the proteins of interest during a 4 min gradient from 20 to 50% B. The column was washed and equilibrated by 0.5 min at 71.4% B, 0.1 min gradient to 100% B, 2 min wash at 100% B, 0.1 min gradient to 20% B, and then a 2.2 min hold at 20% B, giving a total 10 min run time. An MS scan range of 100–3000 *m/z* with a spectral rate of 2 Hz was used. External calibration was performed prior to data collection.

### LC–MS(/MS) analysis of glycopeptide modification

Glycopeptides for LC-MS(/MS) analysis were prepared by digesting His-tag purified, dialyzed glycosylation targets with 0.0044 µg/µl MS Grade Trypsin (Thermo Fisher Scientific) at 37 °C overnight. Before injection, H1HA10 samples were reduced by incubation with 10 mM DTT for 2 h. LC-MS(/MS) was performed by injection of 2 µl (or about 2 pmol) of digested glycopeptides into a Bruker Elute UPLC equipped with an ACQUITY UPLC Peptide BEH C18 Column, 300 Å, 1.7 µm, 2.1 mm × 100 mm (186003686 Waters Corp.) with a 10 mm guard column of identical packing (186004629 Waters Corp.) coupled to an Impact-II UHR TOF Mass Spectrometer. Liquid chromatography was performed using 100% H_2_O and 0.1% formic acid as Solvent A and 100% acetonitrile and 0.1% formic acid as Solvent B at a flow rate of 0.5 mL/min and a 40 °C column temperature. An initial condition of 0% B was held for 1 min before elution of the peptides of interest during a 4 min gradient to 50% B. The column was washed and equilibrated by a 0.1 min gradient to 100% B, a 2 min wash at 100% B, a 0.1 min gradient to 0% B, and then a 1.8 min hold at 0% B, giving a total 9 min run time. LC-MS/MS of glycopeptides was performed to confirm that GT modifications were in accordance with previously characterized specificities. Pseudo multiple reaction monitoring (MRM) MS/MS fragmentation was targeted to theoretical glycopeptide masses corresponding to detected intact protein MS peaks. All glycopeptides were fragmented using a collisional energy of 30 eV with a window of ±2 *m/z* from targeted *m/z* values. Theoretical protein, peptide, and sugar ion masses derived from expected glycosylation structures are shown in Supplementary Tables 3 and 5–7. For LC-MS and LC-MS/MS of glycopeptides, a scan range of 100–3000 *m/z* with a spectral rate of 8 Hz was used. External calibration was performed prior to data collection.

### Exoglycosidase digestions

When possible, sugar linkages installed by various GTs and biosynthetic pathways were confirmed by exoglycosidase digestion using commercially available enzymes from New England Biolabs with well-characterized activities. As indicated in figures and figure legends, glycoproteins or glycopeptides were incubated with exoglycosidases for at least 4 h at 37 °C using buffers and digestion conditions suggested by the manufacturer. The exoglycosidases and associated product numbers used in this study are: β1-4 Galactosidase S (P0745S); α1-3,6 Galactosidase (P0731S); α1-3,4 Fucosidase (P0769S); and α1-2 Fucosidase (P0724S); α1-3,4,6 Galactosidase (P0747S); β-*N*-Acetylglucosaminidase S (P0744S); α2-3 Neuraminidase S (P0743S); and α2-3,6,8 Neuraminidase (P0720S).

### LC-MS(/MS) data analysis

LC-MS(/MS) data was collected using Bruker Compass Hystar v4.1 and analyzed using Bruker Compass Data Analysis v4.1 (Bruker Daltonics, Inc.). Glycopeptide MS and intact glycoprotein MS spectra were averaged across the full elution times of the glycosylated and aglycosylated glycoforms (as determined by extracted ion chromatograms of theoretical glycopeptide and glycoprotein charge states). MS spectra for intact glycoproteins was then analyzed by Data Analysis maximum entropy deconvolution from the full m/z scan range of 100–2000 into a mass range of 10,000–14,000 Da for Im7-6 samples or 27,000–29,000 Da for Fc-6 samples. Representative LC-MS/MS spectra from MRM fragmentation were selected and annotated manually. Observed glycopeptide *m/z* and intact protein deconvoluted masses are annotated in figures and theoretical values are shown in Supplementary Tables 3 and 5–7. LC-MS(/MS) data was exported from Bruker Compass Data Analysis and plotted in Microsoft Excel 365.

### Statistical information

Figure legends indicate exact sample numbers for means, standard deviations (error bars), and representative data for each experiment. No tests for statistical significance or animal subjects were used in this study.

### Reporting summary

Further information on research design is available in the Nature Research Reporting Summary linked to this article.

## Supplementary information

Supplementary Information

Reporting Summary

## Data Availability

All data generated or analyzed during this study are included in this article and its supplementary materials or are available from the corresponding authors upon reasonable request. The source data underlying the averages reported in Supplementary Table 2 are provided as a Source Data file.

## Source data

Source Data
