# Supplementary material for: A cell-free biosynthesis platform for modular construction of protein glycosylation pathways
Source: Nat Commun. 2019 Nov 27;10:5404. doi: 10.1038/s41467-019-12024-9 (PMC6881289; doi:10.1038/s41467-019-12024-9)
Supplement: Supplementary file 1 — Supplementary Information [file 41467_2019_12024_MOESM1_ESM.pdf]

Supplementary Information

A cell-free biosynthesis platform for modular construction of protein glycosylation pathways

Kightlinger et al.

Supplementary Tables

Supplementary Table 1: Summary of all strains and plasmids used in this study[1-6]. Plasmid backbone characteristics are listed followed by Uniprot or NCBI identifiers of protein-coding sequences and any modifications or fusion sequences. Annotated protein-coding sequences of all plasmids developed in this study are shown with flanking plasmid sequence contexts in **Supplementary Note 1**.

Plasmid and Strain	Relevant Characteristics	Source
**Strains**		
DH5-α	*fhuA2 Δ(argF-lacZ)U169 phoA glnV44 Φ80 Δ(lacZ)M15 gyrA96 recA1 relA1 endA1 thi-1 hsdR17*	New England Biolabs
C321.ΔA.759	MG1655 C321 Derivative	1
CLM24Δ*nanA*	W3110 ΔwecA Δ*nanA* Δ waaL::Kan	This Study
**Plasmids**		
pJL1.sfGFP	pJL1 plasmid = Kan^R, P_{T7}, pBR322 ori; Insert = super folder green fluorescent protein (sfGFP), C-term strep-tag	2
pJL1.NGT	pJL1 plasmid; Insert = *A. pleuroneumoniae* NGT, (NGT_ACTP2)	3
pJL1.AGT	pJL1 plasmid; Insert = *A. pleuroneumoniae* α 1-6 GlcT, (GTF_ACTP7)	3
pJL1.Im7-6	pJL1 plasmid; Insert = *E. coli* Im7, (IMM7_ECOLX), 26_31=ATTGGNWTTAGG, C-term 6xHis-tag	3
PJL1.BfGalNAcT	pJL1 plasmid; Insert = Q5LJ88_BACFN	This Study
PJL1.Hpβ4GalT	pJL1 plasmid; Insert = D0IS16_HELP1	This Study
PJL1.NmLgtB	pJL1 plasmid; Insert = LGTB_NEIMB	This Study
PJL1.Btβ4GalT	pJL1 plasmid; Insert = B4GT1_BOVIN, N-term Strep-tag	This Study
PJL1.NgLgtB	pJL1 plasmid; Insert = Q5F4Y6_NEIG1	This Study
PJL1.SpwchJ	pJL1 plasmid; Insert = Q4K235_STREE	This Study
PJL1.SpwchK	pJL1 plasmid; Insert = Q4K234_STREE	This Study
PJL1.NmLgtC	pJL1 plasmid; Insert = Q93EK7_NEIME	This Study
PJL1.HsSIAT1	pJL1 plasmid; Insert = SIAT1_HUMAN	This Study
PJL1.PmST3,6	pJL1 plasmid; Insert = Q15KI8_PASMD, N-Term CAT_Strep-tag_Linker =CSL	This Study
PJL1.CjCST-I	pJL1 plasmid; Insert = Q9RGF1_CAMJU, N-term CSL	This Study
PJL1.CjCST-II	pJL1 plasmid; Insert = Q9LAK3_CAMJU, N-term CSL	This Study
PJL1.SpPvg1	pJL1 plasmid; Insert = PVG1_SCHPO, N-term CSL	This Study
PJL1.VsST3	pJL1 plasmid; Insert = A8R0Y0_9VIBR, N-term CSL	This Study
PJL1.HpFutA	pJL1 plasmid; Insert = FUCT_HELPX, Δ363_478, C-term Strep-tag	This Study
PJL1.HpFutC	pJL1 plasmid; Insert = A0A1V0EFK2_HELPX, N-term CSL	This Study
PJL1.BtGGTA	pJL1 plasmid; Insert = GGTA1_BOVIN, Δ1_80	This Study
PJL1.HdGlcNAcT	pJL1 plasmid; Insert = Q8GNC0_HAEDC	This Study
PJL1.NgLgtA	pJL1 plasmid; Insert = LgtA_NP274923.1	This Study
PJL1.PdST6	pJL1 plasmid; Insert = O66375_9GAMM, N-term CSL	This Study
PJL1.PlST6	pJL1 plasmid; Insert = D0VYB7_PHOLE, N-term CSL	This Study
PJL1.PpST3	pJL1 plasmid; Insert = A5LHX0_PHOPO	This Study
PJL1.H1HA10	pJL1 plasmid; Insert = H1HA10 Synthetic Immunogen, N-term His-tag_ATTGGNWTTAGG	This Study
pMAF10.NGT	pMAF10 plasmid = $Trimethylprim^R$, P_{BAD}, MOB ori; Insert = NGT_ACTP2, C-term FLAG-tag	3
pMAF10.ApNGT.NmLgtB	pMAF10 plasmid; Insert = NGT_ACTP2, C-term FLAG-tag; LGTB_NEIMB, N-term CSL	This Study
pMAF10.CjCST-I.NmLgtB.ApNGT	pMAF10 plasmid; Insert = Q9RGF1_CAMJU, N-term CSL; LGTB_NEIMB, N-term CSL; NGT_ACTP2, C-term FLAG-tag	This Study
pMAF10.PdST6.NmLgtB.ApNGT	pMAF10 plasmid; Insert = O66375_9GAMM, N-term CSL; LGTB_NEIMB, CSL; NGT_ACTP2, C-term FLAG-tag	This Study
pBR322.Im7	pBR322 plasmid = $Carb^R$, P_{trc}, pBR322 ori; Insert = IMM7_ECOLX, C-terminal 6xHis-tag	4
pBR322.Im7-6	pBR322 plasmid; Insert = *E. coli* Im7, (IMM7_ECOLX), 26_31=ATTGGNWTTAGG, C-term 6xHis-tag	This Study
pBR322.Fc-6	pBR322 plasmid; Insert = *H. sapiens* Fc, IGHG1_HUMAN, Q178_Y183=ATTGGNWTTAGG, Δ1_98, C-term 6xHis-tag	This Study
pConYCG	pConYCG plasmid = CM^R, P_{23109}, rrnB Terminator, p15A ori	5
pTF	pMW07 plasmid = Yeast Recombineering plasmid, Cm^R, P_{BAD}, p15A ori; Insert = galE, pglB, pglA, wbnJ, EcNeuDBAC	6
pConNeuA	pConYCG plasmid, P_{23109}::P_{23100} (Constitutive); Insert = B1LEC0_ECOSM (EcNeuA)	This Study

Supplementary Table 2: Optimization of cell-free protein synthesis of Im7 target and glycosylation enzymes. (a) CFPS yields of Im7-6 target and enzymes for *in vitro* glycosylation pathways tested by GlycoPRIME. CFPS yields and errors indicate mean and s.d. from n=3 CFPS reactions quantified by [^{14}C]-leucine incorporation. All CFPS reactions were incubated for 20 h at the indicated temperatures and conditions. Solubility was calculated from quantification of yields in fractions isolated after centrifugation at 12,000xg for 15 mins. Asterisk (*) indicates yields when CFPS was conducted under oxidizing conditions. Dotted lines indicate if enzymes were used in biosynthetic pathways with one, two, or more than three GTs. Yields under optimized conditions also shown in **Figs. 2 and 3**. Source data underlying listed average and s.d. values are provided in the **Source Data** file.

**Protein Name**	**Total Protein Yield in CFPS (μg/mL)**			**Protein Solubility in CFPS (%)**			**Optimum Temperature (°C)**
	16°C	23°C	30°C	16°C	23°C	30°C	
Im7-6	107 ± 13	538 ± 26	488 ± 51	129 ± 30	67 ± 9	60 ± 15	23
ApNGT	375 ± 41	1420 ± 164	1347 ± 208	86 ± 11	66 ± 10	44 ± 15	23
Apα1-6	648 ± 34	1548 ± 155	2065 ± 275	65 ± 8	57 ± 8	32 ± 5	23
NmLgtB	240 ± 70	703 ± 50	1136 ± 109	91 ± 28	89 ± 6	59 ± 8	23
Hpβ4GalT	339 ± 19	880 ± 129	1443 ± 157	32 ± 6	3 ± 2	4 ± 3	16
Btβ4GalT1	222 ± 13	671 ± 39	937 ± 120	61 ± 6	41 ± 5	15 ± 3	23
Btβ4GalT1*	187 ± 12	492 ± 76	1001 ± 127	61 ± 8	31 ± 9	15 ± 4	23
NgLgtB	447 ± 35	913 ± 36	1224 ± 158	81 ± 11	97 ± 4	89 ± 15	30
SpWchK	111 ± 4	365 ± 11	625 ± 57	68 ± 10	26 ± 3	6 ± 1	23
SpWchJ	622 ± 24	1814 ± 188	1774 ± 140	24 ± 2	7 ± 1	7 ± 1	23
BfGalNAcT	169 ± 15	285 ± 20	460 ± 77	79 ± 12	68 ± 8	55 ± 18	23
BtGGTA	247 ± 8	790 ± 16	1179 ± 100	66 ± 7	20 ± 3	30 ± 5	30
NmLgtC	125 ± 39	270 ± 67	458 ± 16	105 ± 44	81 ± 25	101 ± 16	30
HdGlcNAcT	126 ± 24	377 ± 25	772 ± 131	87 ± 21	37 ± 4	15 ± 4	23
PsPvg1	170 ± 9	662 ± 8	1600 ± 55	89 ± 8	36 ± 1	11 ± 2	23
HpFutA	575 ± 10	1670 ± 133	1852 ± 168	39 ± 4	34 ± 4	33 ± 8	23
NgLgtA	487 ± 13	1199 ± 72	2076 ± 36	84 ± 9	62 ± 5	55 ± 2	30
PdST6	242 ± 31	804 ± 48	1274 ± 136	98 ± 15	86 ± 10	68 ± 12	23
HsSIAT1	175 ± 21	770 ± 92	928 ± 129	34 ± 18	3 ± 3	5 ± 3	16
HsSIAT1*	506 ± 49	674 ± 41	637 ± 113	30 ± 5	18 ± 3	13 ± 3	16
PlST6	480 ± 58	1063 ± 160	1366 ± 31	113 ± 26	102 ± 18	117 ± 17	30
CjCST-I	343 ± 44	649 ± 63	1248 ± 93	40 ± 6	37 ± 6	44 ± 8	16
CjCST-I*	147 ± 16	712 ± 54	920 ± 61	77 ± 12	17 ± 2	12 ± 4	16
VsST3	247 ± 55	601 ± 80	1044 ± 56	102 ± 31	86 ± 18	105 ± 10	30
PpST3	323 ± 50	738 ± 70	1146 ± 143	135 ± 33	103 ± 22	118 ± 16	30
CjCST-II	203 ± 33	674 ± 16	1285 ± 143	103 ± 20	58 ± 8	66 ± 11	23
PmST3,6	476 ± 65	1252 ± 187	2048 ± 201	34 ± 7	17 ± 6	6 ± 1	23
HpFutC	598 ± 48	1557 ± 69	1714 ± 60	40 ± 7	18 ± 7	1 ± 2	16

Supplementary Table 3: Theoretical glycoprotein and glycopeptide masses for Im7-6 glycoforms produced during GlycoPRIME biosynthetic pathway engineering. Predicted glycosylation structures are based on previously established GT activities shown in **Figs. 2 and 3 and Supplementary Table 4**. Theoretical, neutral, and average masses of expected glycoprotein products as well as theoretical, triply charged, monoisotopic mass-to-charge ratios (m/z) of glycopeptides are shown below. Glycopeptide masses correspond to the only ApNGT glycosylation site within Im7-6 which is contained within the tryptic peptide EATTGGNWTTAGGDVLDVLLEHFVK. Experimentally observed masses are annotated in deconvoluted intact protein MS and glycopeptide MS/MS spectra.

Target Protein	Predicted Glycan Structure	Biosynthetic Pathway	Glycoprotein	Glycopeptide	
			Theoretical Mass	Charge	Theoretical m/z
Im7-6	None	None	11366.43	3	877.44
Im7-6	Glcβ-Asn	ApNGT	11528.48	3	931.46
Im7-6	Galβ4Glcβ-Asn	ApNGT+NmLgtB or NgLgtB	11690.53	3	985.47
Im7-6	GalNAcβ3Glcβ-Asn	ApNGT+BfGalNAcT	11731.67	3	999.19
Im7-6	Glcα6Glcβ-Asn	ApNGT+Apα1-6	11690.53	3	985.47
Im7-6	(Glcα6)^{2}Glcβ-Asn	ApNGT+Apα1-6	11852.58	3	1039.49
Im7-6	(Glcα6)^{3}Glcβ-Asn	ApNGT+Apα1-6	12014.63	3	1093.51
Im7-6	(Glcα6)^{4}Glcβ-Asn	ApNGT+Apα1-6	12176.68	3	1147.52
Im7-6	(Glcα6)^{5}Glcβ-Asn	ApNGT+Apα1-6	12338.73	3	1201.54
Im7-6	(Glcα6)^{6}Glcβ-Asn	ApNGT+Apα1-6	12500.78	3	1255.56
Im7-6	(Glcα6)^{7}Glcβ-Asn	ApNGT+Apα1-6	12662.83	3	1309.57
Im7-6	(Glcα6)^{8}Glcβ-Asn	ApNGT+Apα1-6	12824.88	3	1363.59
Im7-6	(Glcα6)^{9}Glcβ-Asn	ApNGT+Apα1-6	12986.93	3	1417.61
Im7-6	(Glcα6)^{10}Glcβ-Asn	ApNGT+Apα1-6	13148.98	3	1471.62
Im7-6	(Glcα6)^{11}Glcβ-Asn	ApNGT+Apα1-6	13311.03	3	1525.64
Im7-6	(Glcα6)^{12}Glcβ-Asn	ApNGT+Apα1-6	13473.08	3	1579.66
Im7-6	PyrGalβ4Glcβ-Asn	ApNGT+NmLgtB+SpPvg1	11760.53	3	1008.81
Im7-6	Galβ4(Fucα3)Glcβ-Asn	ApNGT+NmLgtB+HpFutA	11836.59	3	1034.16
Im7-6	Fucα2Galβ4Glcβ-Asn	ApNGT+NmLgtB+HpFutC	11836.59	3	1034.16
Im7-6	Galα3Galβ4Glcβ-Asn	ApNGT+NmLgtB+BtGGTA	11852.58	3	1039.49
Im7-6	Galα4Galβ4Glcβ-Asn	ApNGT+NmLgtB+NmLgtC	11852.58	3	1039.49
Im7-6	GlcNAcβ3Galβ4Glcβ-Asn	ApNGT+NmLgtB+NmLgtA	11893.72	3	1053.20
Im7-6	Siaα3Galβ4Glcβ-Asn	ApNGT+NmLgtB+α2,3SiaT	11981.63	3	1082.50
Im7-6	Siaα6Galβ4Glcβ-Asn	ApNGT+NmLgtB+α2,6SiaT	11981.63	3	1082.50
Im7-6	Galβ4GlcNAcβ3Galβ4Glcβ-Asn	ApNGT+NmLgtB+NmLgtA	12055.77	3	1107.22
Im7-6	(Galβ4GlcNAcβ3)^{2}Galβ4Glcβ-Asn	ApNGT+NmLgtB+NmLgtA	12421.01	3	1228.97
Im7-6	(Galβ4GlcNAcβ3)^{3}Galβ4Glcβ-Asn	ApNGT+NmLgtB+NmLgtA	12786.25	3	1350.71
Im7-6	(Galβ4GlcNAcβ3)^{4}Galβ4Glcβ-Asn	ApNGT+NmLgtB+NmLgtA	13151.49	3	1472.46
Im7-6	(Galβ4GlcNAcβ3)^{5}Galβ4Glcβ-Asn	ApNGT+NmLgtB+NmLgtA	13516.73	3	1594.21
Im7-6	Fucα2Galβ4(Fucα3)Glcβ-Asn	ApNGT+NmLgtB+FutA+FutC	11982.64	3	1082.85
Im7-6	Siaα3Galβ4(Fucα3)Glcβ-Asn	ApNGT+NmLgtB+HpFutA+CjCST-I	12127.69	3	1131.19
Im7-6	Siaα6(α2Fuc)Galβ4Glcβ-Asn	ApNGT+NmLgtB+HpFutC+PdST6	12127.69	3	1131.19
Im7-6	Siaα6(α3Sia)Galβ4Glcβ-Asn	ApNGT+NmLgtB+CjCST-I+PdST6	12272.72	3	1179.54
Im7-6	(Fucα2)(Galβ4GlcNAcβ3)Galβ4Glcβ-Asn	ApNGT+NmLgtB+HpFutC+NmLgtA	12201.83	3	1155.91
Im7-6	(Fucα2)(Galβ4GlcNAcβ3)^{2}Galβ4Glcβ-Asn	ApNGT+NmLgtB+HpFutC+NmLgtA	12567.07	3	1277.65
Im7-6	(Fucα2)(Galβ4GlcNAcβ3)^{3}Galβ4Glcβ-Asn	ApNGT+NmLgtB+HpFutC+NmLgtA	12932.31	3	1399.40
Im7-6	(Fucα2)(Galβ4GlcNAcβ3)^{4}Galβ4Glcβ-Asn	ApNGT+NmLgtB+HpFutC+NmLgtA	13297.55	3	1521.15
Im7-6	Galβ4(Fucα3)GlcNAcβ3Galβ4(Fucα3)Glcβ-Asn	ApNGT+NmLgtB+HpFutA+NmLgtA	12347.89	3	1204.59
Im7-6	Siaα6Galβ4GlcNAcβ3(Siaα6)Galβ4Glcβ-Asn	ApNGT+NmLgtB+PdST6+NmLgtA	12637.96	3	1301.28
Im7-6	(Siaα6Galβ4GlcNAcβ3)^{2}(Siaα6)Galβ4Glcβ-Asn	ApNGT+NmLgtB+PdST6+NmLgtA	13294.30	3	1520.06
Im7-6	(Siaα6Galβ4GlcNAcβ3)^{3}(Siaα6)Galβ4Glcβ-Asn	ApNGT+NmLgtB+PdST6+NmLgtA	13950.63	3	1738.84
Im7-6	Siaα3(Galβ4GlcNAcβ3)Galβ4Glcβ-Asn	ApNGT+NmLgtB+CjCST-I+NmLgtA	12346.87	3	1204.25
Im7-6	Siaα3(Galβ4GlcNAcβ3)^{2}Galβ4Glcβ-Asn	ApNGT+NmLgtB+CjCST-I+NmLgtA	12712.11	3	1326.00
Im7-6	Siaα3(Galβ4GlcNAcβ3)^{3}Galβ4Glcβ-Asn	ApNGT+NmLgtB+CjCST-I+NmLgtA	13077.35	3	1447.75
Im7-6	Siaα3(Galβ4GlcNAcβ3)^{4}Galβ4Glcβ-Asn	ApNGT+NmLgtB+CjCST-I+NmLgtA	13442.59	3	1569.49
Im7-6	Galβ4(Fucα3)GlcNAcβ3(Siaα6)Galβ4Glcβ-Asn	ApNGT+NmLgtB+FutA+PdST6+NmLgtA	12492.92	3	1252.94
Im7-6	(Siaα6)(Fucα2)(Galβ4GlcNAcβ3Galβ4))Glcβ-Asn	ApNGT+NmLgtB+FutC+PdST6+NmLgtA	12492.92	3	1252.94
Im7-6	Siaα6(Fucα2)(Galβ4GlcNAcβ3(Siaα6)Galβ4))Glcβ-Asn	ApNGT+NmLgtB+FutC+PdST6+NmLgtA	12784.02	3	1349.97
Im7-6	(Siaα6)^{2}(Fucα2)(Galβ4GlcNAcβ3)^{2}Galβ4Glcβ-Asn	ApNGT+NmLgtB+FutC+PdST6+NmLgtA	13149.26	3	1471.72

Supplementary Table 4: Previously characterized activities of glycosyltransferases used this study[7-23]. GTs listed below were selected for testing in the GlycoPRIME system based on their previously established activities. Many have also been previously used for biosynthesis of glycolipids or free oligosaccharides, laying the foundation for their testing in the new context of elaborating the *N*-linked glucose installed by ApNGT in this study.

Enzyme	Organism	Previously Characterized Activity	Reference
ApNGT	*A. pleuropneumoniae*	βGlc → Asn	7
Apα1-6	*A. pleuropneumoniae*	(α1-6 Glc)^{n} → Glc	8
NgLgtB	*N. gonorrhoeae*	β1-4 Gal → Glc(Nac)	9
Hpβ4GalT	*H. pylori*	β1-4 Gal → Glc(Nac)	10
Btβ4GalT1	*B. taurus*	β1-4 Gal → Glc(Nac)	11
NmLgtB	*N. meningititus*	β1-4 Gal → Glc(Nac)	9
SpWchK	*S. pleuropnemoniae*	β1-4 Gal → Glc	12
SpWchJ	*S. pleuropnemoniae*	WchK enhancer	12
BfGalNAcT	*B. fragilis*	β1-3 GalNAc → Glc	13
NgLgtA	*N. gonorrhoeae*	β1-3 GlcNAc → Gal	14
PsPvg1	*S. pombe*	Pyruvate → Gal	16
HpFutA	*H. pylori*	α1-3 Fuc → Glc(Nac)	15
HpFutC	*H. pylori*	α1-2 Fuc → Gal	17
NmLgtC	*N. meningititus*	α1-4 Gal → Gal	17
BtGGTA	*B. taurus*	α1-3 Glc → Glc	18
HsSIAT1	*H. sapiens*	α2-6 Sia → Gal	19
PdST6	*B. taurus*	α2-6 Sia → Gal	20
PlST6	*P. leiognathi*	α2-6 Sia → Gal	21
PmST3,6	*P. multocida*	α2-3,6 Sia → Gal	21
VsST3	*V. sp. JT-FAJ-16*	α2-3 Sia → Gal	21
PpST3	*P. phosphoreum*	α2-3 Sia → Gal	21
CjCST-I	*C. jejuni*	α2-3 Sia → Gal	21
CjCST-II	*C. jejuni*	α2-3,8 Sia → Gal	22
HdGlcNAcT	*H. ducreyi*	β1-3 GlcNAc → Gal	23

Supplementary Table 5: Theoretical masses of sugar fragment ions detected in glycopeptide MS/MS spectra. During MS/MS fragmentation of glycopeptides, diagnostic sugar ions were detected. Theoretical mass to charge ratios of these sugar ions are shown below. All calculations of theoretical m/z assume singly charged ions. All mentions of sialic acid (Sia) in this article refer to *N*-Acetylneuraminic acid (NeuAc).

Sugar Structure	Theoretical m/z
HexNAc	204.20
Sia-H_2O	274.09
Sia	292.10
Hex^2	325.11
HexNAc+Hex	366.25
Hex+Sia	454.15
Hex^3	487.16
HexNAc+Hex+Fuc	512.31
Sia^2	583.20
Hex^4	649.21
HexNAc+Hex+Sia	657.34
$(HexNAc+Hex)^2$	731.49
HexNAc+Hex+Fuc+Sia	803.40
$(HexNAc+Hex)^3$	1096.73
$(HexNAc+Hex)^4$	1461.97

Supplementary Table 6: Theoretical glycopeptide masses for H1AH10 synthesized and glycosylated *in vitro*. Theoretical, doubly charged, monoisotopic mass-to-charge ratios (m/z) of the tryptic peptide containing the *N*-terminal, engineered glycosylation site within H1AH10 which was synthesized and glycosylated a one-pot *in vitro* reaction. Predicted glycosylation structures are based on previously established GT activities shown in **Figs. 2 and 3 and Supplementary Table 4**. Experimentally observed masses are annotated on deconvoluted MS and MS/MS spectra in **Fig. 4 and Supplementary Fig. 14**.

Target Protein	Glycopeptide Sequence	Predicted Glycan Structure	Biosynthetic Pathway	Glycopeptide	
				Charge	Theoretical m/z
H1HA10	ATTGGNWTTAGGK	None	None	2	611.29
H1HA10	ATTGGNWTTAGGK	Glcβ-Asn	ApNGT	2	692.32
H1HA10	ATTGGNWTTAGGK	Galβ4Glcβ-Asn	ApNGT+NmLgtB	2	773.34
H1HA10	ATTGGNWTTAGGK	Galα3Galβ4Glcβ-Asn	ApNGT+NmLgtB+BtGGTA	2	854.37

Supplementary Table 7: Theoretical glycoprotein and glycopeptide masses for Fc-6 synthesized and glycosylated in the *E. coli* cytoplasm. Predicted glycosylation structures are based on previously established GT activities shown in **Figs. 2 and 3 and Supplementary Table 4**. Theoretical, neutral, average masses of expected glycoprotein products and theoretical, triply charged, monoisotopic mass-to-charge ratios (m/z) of glycopeptides are shown below. Glycopeptide masses correspond to the only ApNGT glycosylation site within Fc-6 which is contained within the tryptic peptide EEATTGGNWTTAGGR. Experimentally observed masses are annotated on deconvoluted MS and MS/MS spectra in **Fig. 4 and Supplementary Fig. 15.**

Target Protein	Predicted Glycan Structure	Biosynthetic Pathway	Glycoprotein	Glycopeptide	
			Theoretical Mass	Charge	Theoretical m/z
Fc-6	None	None	27509.09	2	754.34
Fc-6	Glcβ-Asn	ApNGT	27671.14	2	835.36
Fc-6	Galβ4Glcβ-Asn	ApNGT+NmLgtB	27833.19	2	916.39
Fc-6	Siaα3Galβ4Glcβ-Asn	ApNGT+NmLgtB+CjCST-I	28124.29	2	1061.94
Fc-6	Siaα6Galβ4Glcβ-Asn	ApNGT+NmLgtB+PdST6	28124.29	2	1061.94

Supplementary Figures

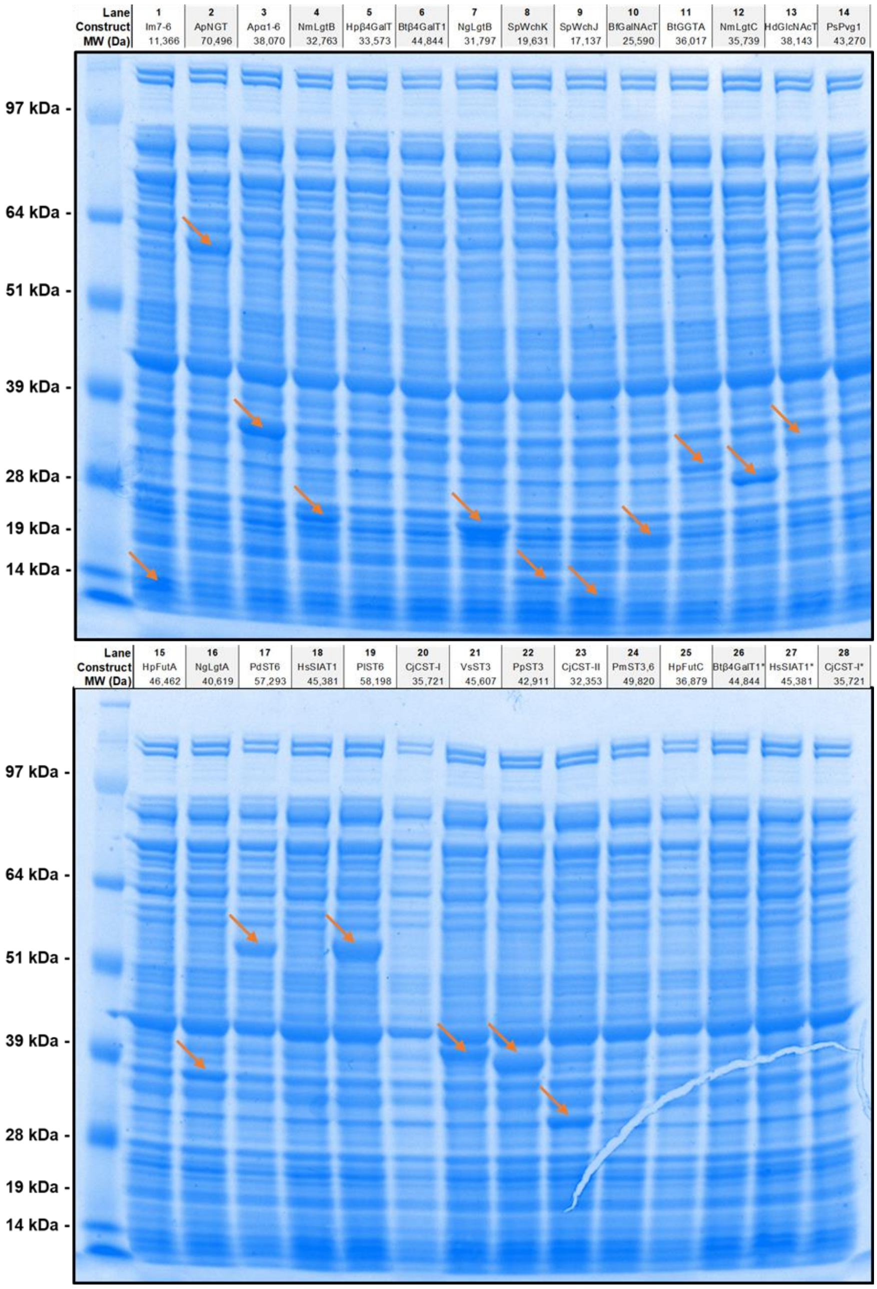

Supplementary Figure 1: Coomassie-stained protein gels showing CFPS expression of GlycoPRIME target and enzymes. Coomassie-stained protein gels of the soluble fractions of *E. coli* crude lysate based CFPS reactions following *in vitro* synthesis of Im7-6 target and indicated GlycoPRIME enzymes. Highly enriched proteins are evident from increased band thicknesses near expected molecular weights (arrows), other products can be seen in **Supplementary Fig. 2**. Products from CFPS reactions run under oxidizing conditions indicated by (*). Soluble samples were isolated by centrifugation at 12,000xg for 15 min at 4°C. Representative of n=2 gels. The same gels were exposed as autoradiograms to determine bands containing [^{14}C]-leucine protein (**Supplementary Fig. 2**).

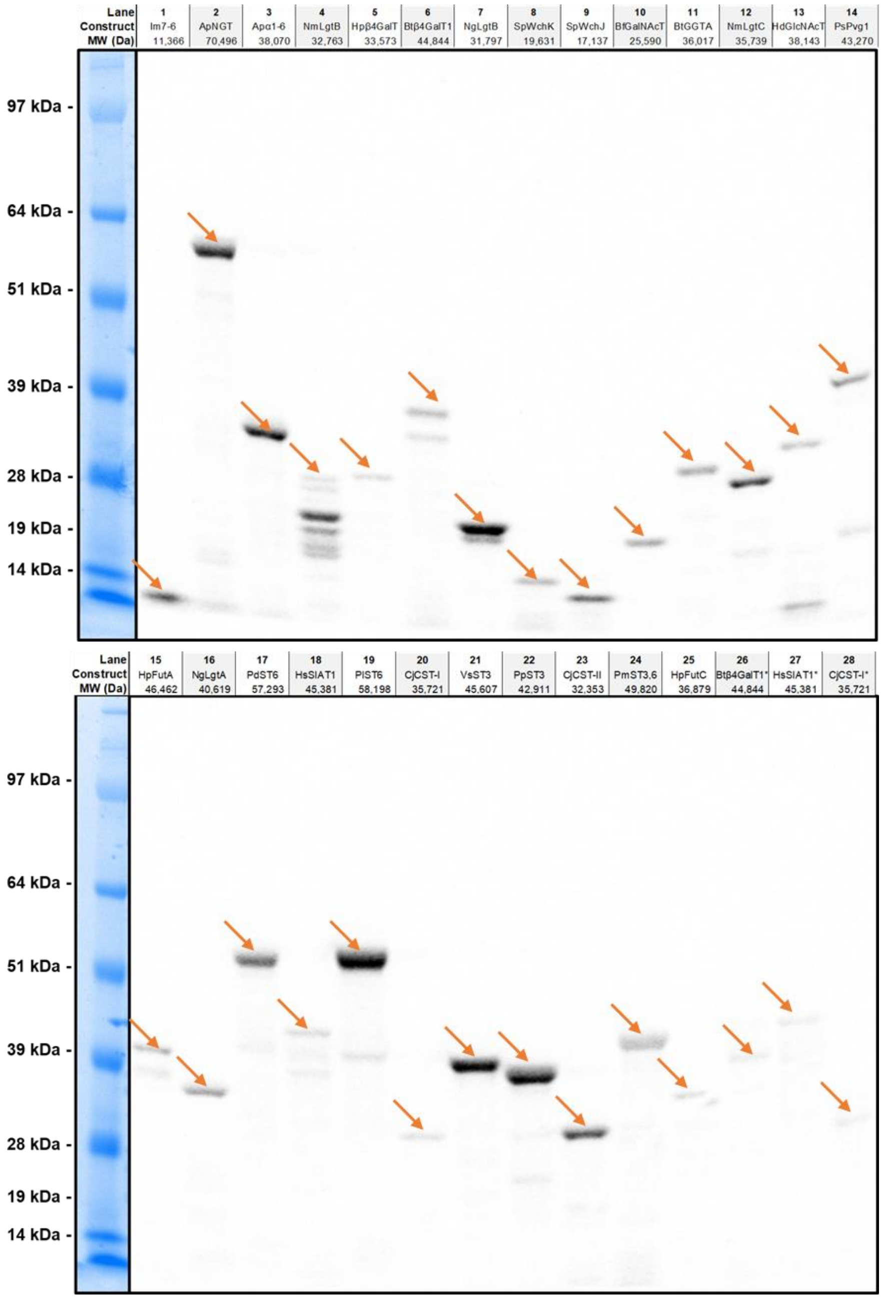

Supplementary Figure 2: Autoradiograms of protein gels showing CFPS expression of GlycoPRIME target and enzymes in CFPS. Autoradiograms of protein gels of the soluble fractions of *E. coli* crude lysate based CFPS reactions containing [^{14}C]-leucine following *in vitro* synthesis of Im7-6 target and indicated GlycoPRIME enzymes. The presence of bands containing [^{14}C]-leucine near expected molecular weights indicate full-length expression of proteins without large truncations (arrows indicate expected full-length product). Products from CFPS reactions run under oxidizing conditions indicated by (*). Soluble samples were isolated by centrifugation at 12,000xg for 15 min at 4°C. The autoradiograms were generated by exposing a 4-12% SDS-PAGE gel run in MOPS to a phosphoscreen for a 72-h. The autoradiogram is representative of n=2 gels and exposures. The same gels were Coomassie stained (**Supplementary Fig. 1**) and aligned with autoradiogram images for molecular weight standard reference.

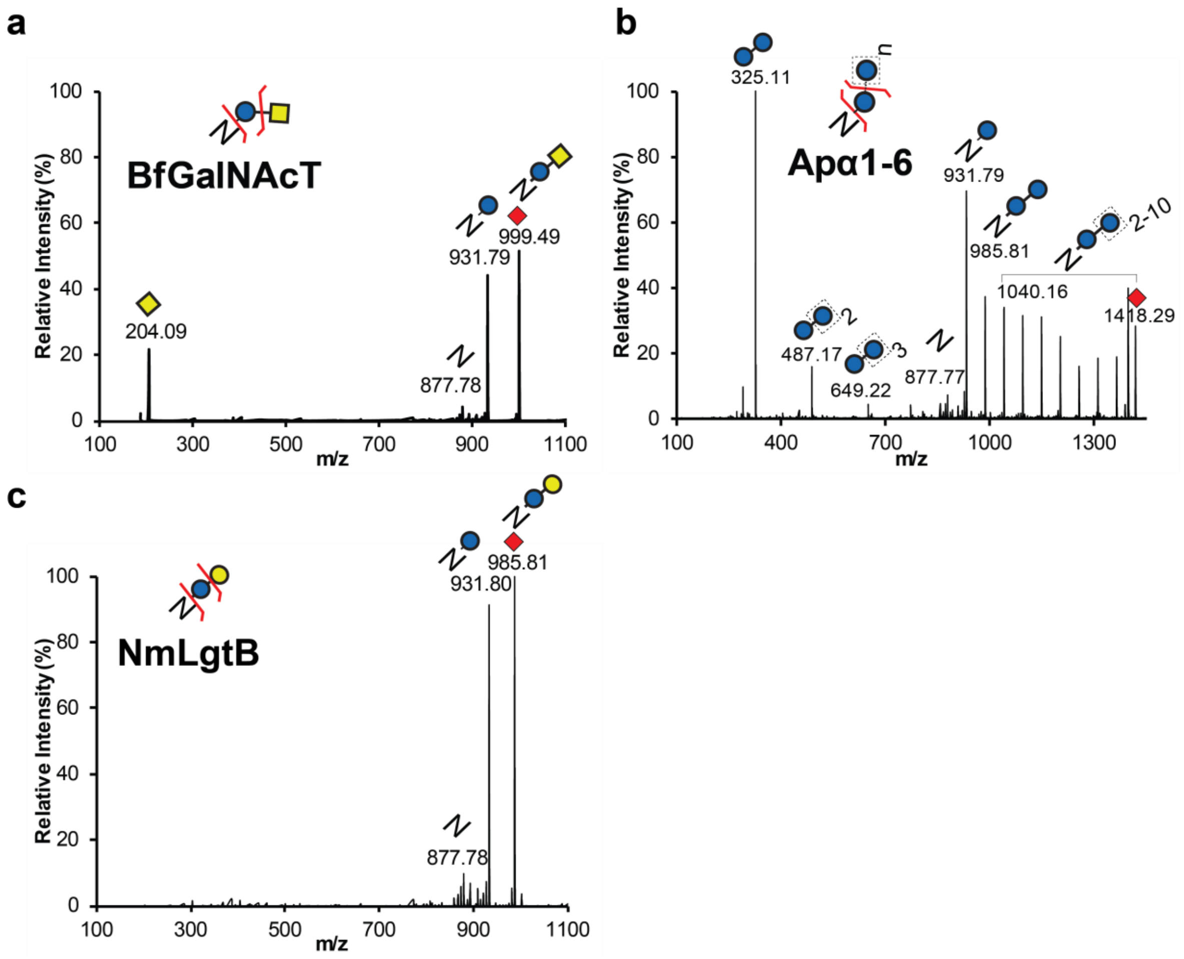

Supplementary Figure 3: **Glycopeptide MS/MS spectra of GlycoPRIME reaction products from two enzyme biosynthetic pathways elaborating *N*-linked glucose.** Products from IVG reactions containing two enzyme pathways modifying Im7-6 shown in **Fig. 2** were purified, trypsinized, and analyzed by pseudo Multiple Reaction Monitoring (MRM) MS/MS fragmentation at theoretical glycopeptide masses (red diamonds) corresponding to detected protein MS peaks using a collisional energy of 30 eV (see **Methods**). Spectra representative of many MS/MS acquisitions from n=1 IVG reaction. Theoretical protein, peptide, and sugar ion masses derived from expected glycosylation structures are shown in **Supplementary Tables 3 and 5**. All indicated sugar ions are singly charged and glycopeptide fragmentation products are triply charged ions consistent with modification of Im7-6 tryptic peptide EATTGGNWTTAGGDVLDVLLEHFVK with indicated sugar structures. **(a)** MS/MS spectra of 999.49 ± 2 m/z corresponding to the peptide modified with *N*-linked GalNAcβ1-3Glc installed by ApNGT and BfGalNAcT. **(b)** MS/MS spectra of 1418.29 ± 2 m/z corresponding to the peptide modified with an *N*-linked dextran polymer installed by ApNGT and Apα1-6. **(c)** MS/MS spectra of 985.81 ± 2 m/z corresponding to the peptide modified with *N*-linked lactose installed by ApNGT and NmLgtB. All IVG reactions contained Im7-6, ApNGT, and appropriate sugar donors according to established enzyme activities (**Supplementary Table 4**).

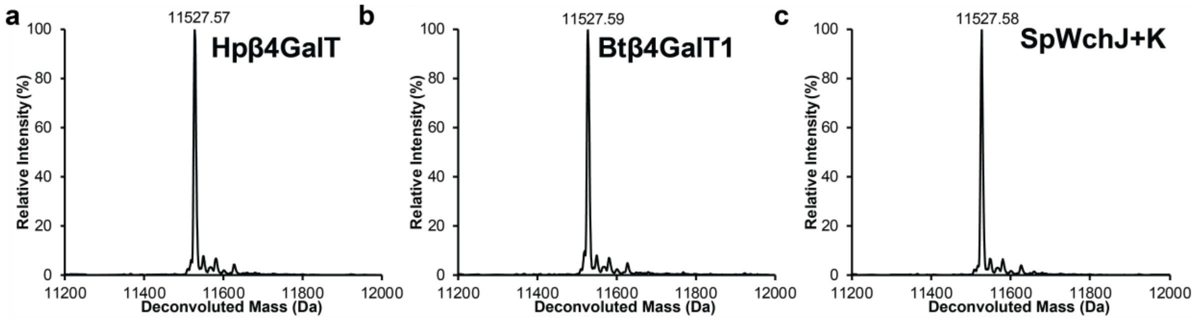

Supplementary Figure 4: **Deconvoluted intact protein MS spectra of IVG reaction products showing no modification of *N*-linked glucose installed by ApNGT.** Products of IVG reactions containing 10 μM Im7-6, 0.4 μM ApNGT, 2.5 mM of appropriate sugar donors, and one elaborating GT were purified and analyzed by intact protein MS (see **Methods**). **(a)** Deconvoluted intact protein MS spectra of IVG containing 1.3 μM of Hpβ4GalT. **(b)** Deconvoluted intact protein MS spectra of IVG containing 1.4 μM of Btβ4GalT1 supplemented with 10 μM α-lactalbumin and performed under oxidizing conditions (see **Methods**). **(c)** Deconvoluted intact protein MS spectra of IVG containing 1.5 μM of SpWchJ and 1.0 μM of SpWchK. No peaks were detected that indicated the modification of Im7-6 with *N*-linked glucose installed by ApNGT (theoretical mass values shown in **Supplementary Table 3**). Spectra from m/z 100-2000 were deconvoluted into 11,000-14,000 Da using Bruker Compass Data Analysis maximum entropy method. Deconvoluted spectra shown here are representative of n=2 IVG reactions.

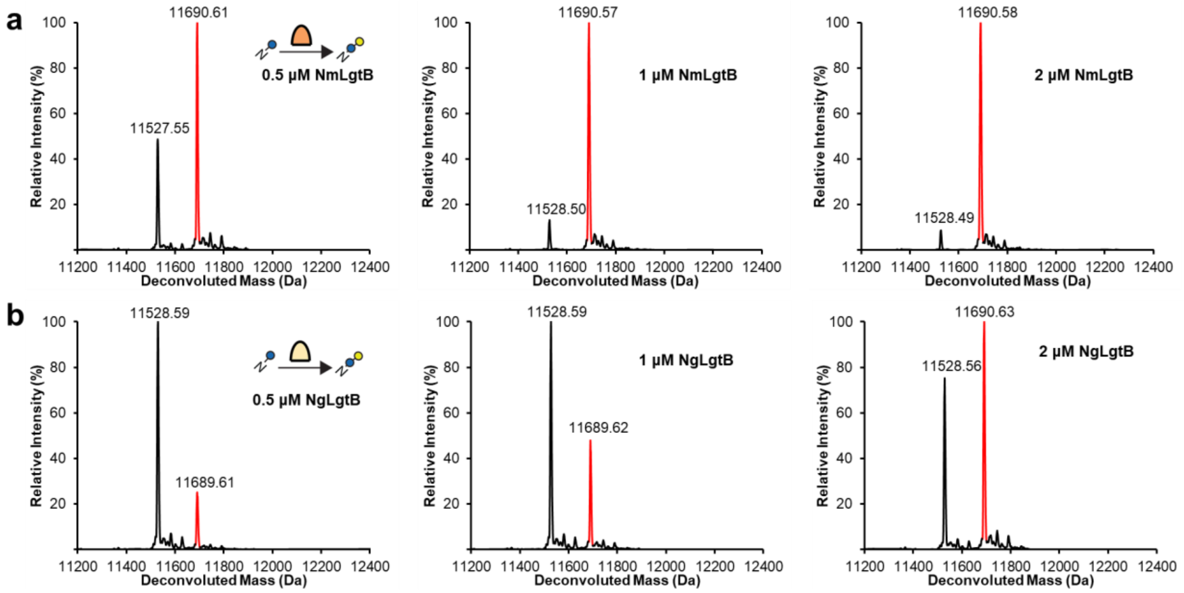

Supplementary Figure 5: **Optimization of LgtB homolog and concentration**. Products of IVG reactions containing 10 µM Im7-6, 0.4 µM ApNGT, 2.5 mM of appropriate sugar donors, and indicated concentrations of NmLgtB or NgLgtB were purified and analyzed by intact protein MS (see **Methods**). **(a)** Deconvoluted intact protein MS spectra from IVG reactions containing indicated concentrations of NmLgtB. **(b)** Deconvoluted intact protein MS spectra from IVG reactions containing indicated concentrations of NgLgtB. Results representative of n=2 IVG reactions conducted for 24 h at 30°C indicate that NmLgtB produced in CFPS has greater specific activity and that nearly homogeneous *N*-linked lactose can be obtained with 2 µM NmLgtB. Theoretical mass values shown in **Supplementary Table 3**. All spectra were acquired from full elution peak areas of all detected glycosylated and aglycosylated Im7-6 species and were deconvoluted from m/z 100-2000 into 11,000-14,000 Da using Bruker Bruker Compass Data Analysis maximum entropy method.

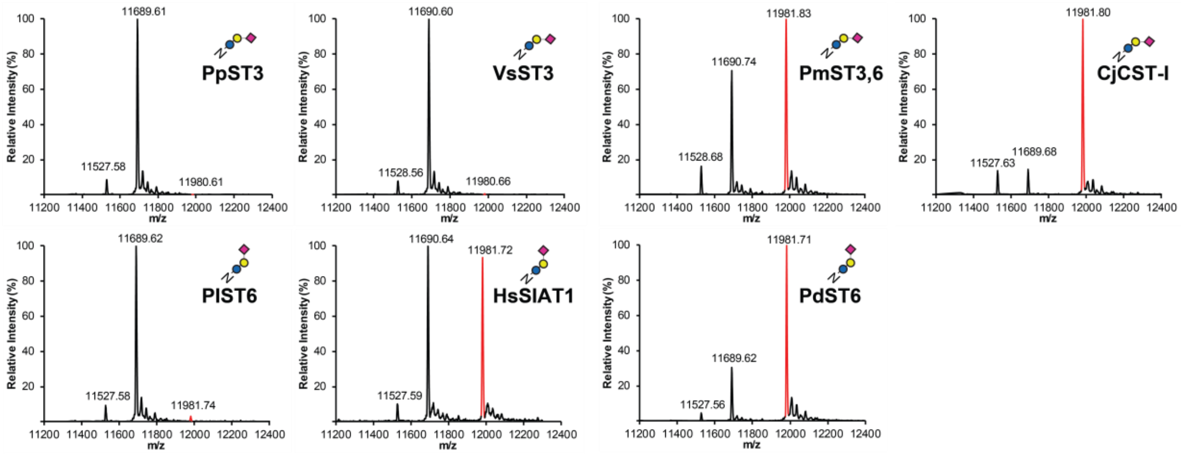

Supplementary Figure 6: **Optimization of sialyltranferase homologs**. Deconvoluted intact protein MS spectra representative of n=2 IVG reactions containing 0.4 μM ApNGT, 2 μM NmLgtB, each sialyltranferase shown in **Fig. 3**, and 2.5 mM each of UDP-Glc, UDP-Gal, and CMP-Sia. Lysates enriched with sialyltransferases by CFPS were added with equal volumes to each IVG reaction such that each 32 μl-IVG reaction contained a total of 25 μl of CFPS lysates. These reactions contained 12.9 μM PpST3; 9.8 μM VsST3; 1.8 μM PmST3,6; 1.3 μM CjCST-I; 5.6 μM PlST6; 0.7 μM of HsSIAT1; and 4.9 μM PdST6, based on CFPS yields shown in **Supplementary Table 2**. CjCST-I and HsSIAT1 were synthesized in CFPS with oxidizing conditions because they were found to be more active when produced in this way (**Supplementary Fig. 9**). Under the conditions above, the reaction containing PdST6 provided the most efficient conversion to 6'-siallylactose and the reaction containing CjCST-I provided the most efficient conversion to 3'-siallylactose (exoglycosidase digestions to confirm linkages are shown in **Supplementary Fig. 10**). Although only trace amounts appear in PpST6 and VsST3, MS/MS detection and identification shows that these enzymes are functional (**Supplementary Fig. 7**). All spectra were acquired from full elution peak areas of all detected glycosylated and aglycosylated Im7-6 species and were deconvoluted from m/z 100-2000 into 11,000-14,000 Da using Bruker Compass Data Analysis maximum entropy method.

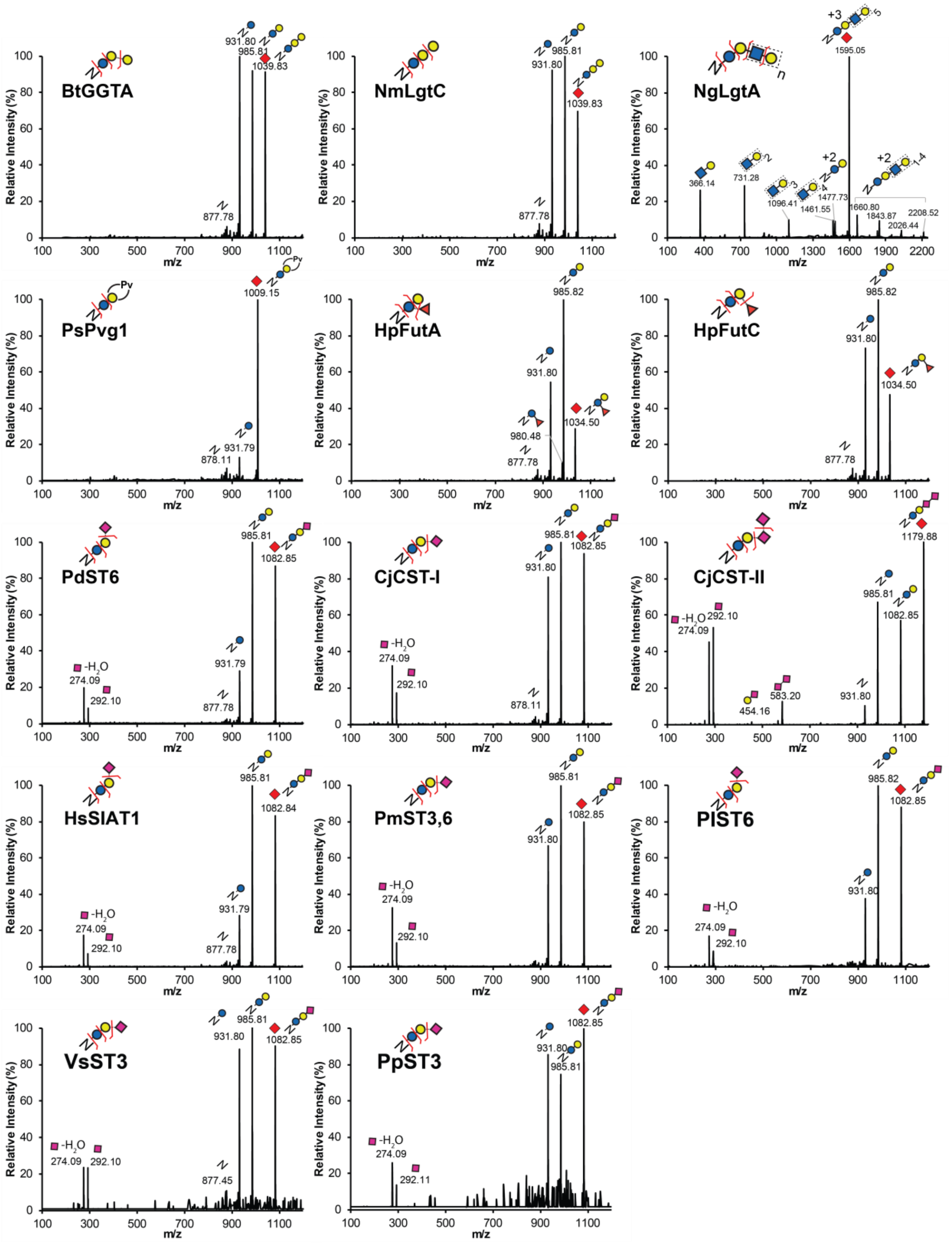

Supplementary Figure 7: Glycopeptide MS/MS spectra of GlycoPRIME reaction products from three enzyme biosynthetic pathways elaborating *N*-linked lactose. Products from IVG reactions containing three enzyme pathways modifying Im7-6 shown in **Fig. 3** were purified, trypsinized, and analyzed by pseudo MRM MS/MS fragmentation at theoretical glycopeptide masses (indicated by red diamonds) corresponding to detected

protein MS peaks in **Fig. 3 and Supplementary Fig. 6**. All glycopeptides were fragmented using a collisional energy of 30 eV with a window of ± 2 m/z from targeted m/z values (see **Methods**). Spectra are representative of many MS/MS acquisitions from n=1 IVG reaction. Theoretical protein, peptide, and sugar ion masses derived from expected glycosylation structures are shown in **Supplementary Tables 3 and 5**. All indicated sugar ions are singly charged and glycopeptide fragmentation products are triply charged ions consistent with modification of Im7-6 tryptic peptide EATTGGNWTTAGGDVLDVLLEHFVK with indicated sugar structures. Predicted sugar linkages based on previously established GT activities (**Supplementary Table 4**) and exoglycosidase sequencing (**Supplementary Figs. 10 and 11**). All IVG reactions contained Im7-6, ApNGT, NmLgtB, indicated GTs, and appropriate sugar donors according to established GT activities.

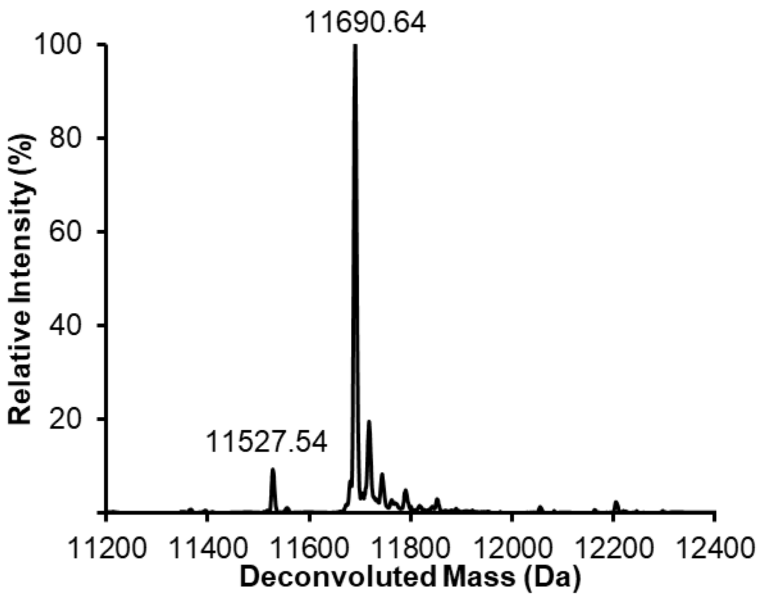

Supplementary Figure 8: **HdGlcNAcT does not modify the *N*-linked lactose substrate installed by ApNGT and NmLgtB.** Deconvoluted intact protein MS spectra of IVG reaction product containing 10 µM Im7-6, 0.4 µM ApNGT, 2 µM NmLgtB, 1.5 µM HdGlcNAcT, and 2.5 mM of UDP-Glc, UDP-Gal, and UDP-GlcNAc. No peaks were detected that indicated the modification of Im7-6 with *N*-linked lactose installed by ApNGT and NmLgtB (see **Supplementary Table 3** for theoretical mass values). Deconvoluted spectra representative of n=2 IVG reactions.

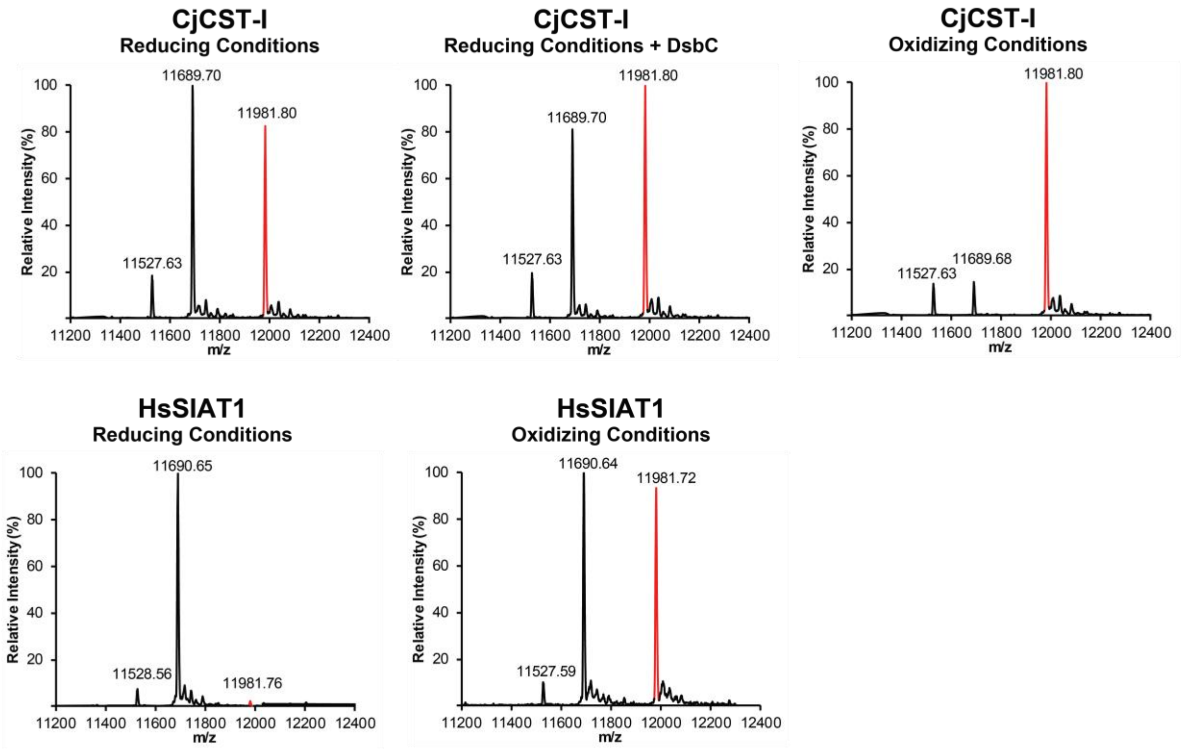

Supplementary Figure 9: **CjCST-I and HsSIAT1 exhibit greater activity when produced in oxidizing conditions**. Deconvoluted intact protein MS spectra representative of of n=2 IVG reaction products containing 10 µM Im7-6, 0.4 µM ApNGT, 2 µM NmLgtB, 2.5 mM of UDP-Glc, UDP-Gal, and CMP-Sia as well as CjCST-I or HsSIAT1 made in CFPS conducted under oxidizing conditions, reducing conditions with supplemented the *E. coli* disulfide bond isomerase (DsbC), or standard reducing conditions (see **Methods**). CFPS conditions are known to create a protein synthesis environment conducive to disulfide bond formation as previously described[24]. Lysates enriched with sialyltranferases by CFPS were added in equal volumes. Therefore, reducing reaction conditions contained 1.9 µM of CjCST-I or 3.8 µM of HsSIAT1 while oxidizing reaction conditions reactions contained 1.3 µM of CjCST-I and 0.7 µM of HsSIAT1 (detailed CFPS yield information shown in **Supplementary Fig. 4**). Aside from CFPS synthesis conditions for the CjCST-I and HsSIAT1, IVG reactions were performed identically without ensuring an oxidizing environment for glycosylation. Im7-6, ApNGT, and NmLgtB were produced with standard CFPS reaction conditions. Relative glycosylation efficiencies indicate that the oxidizing CFPS environment of CFPS allows for greater enzyme activities per unit of CFPS reaction volume and per µM of enzyme. This observation makes sense for HsSIAT1 which is normally active in the oxidizing environment of the human golgi and is known to contain disulfide bonds. Interestingly, an oxidizing synthesis environment also seems to benefit the activity of CjCST-I which does not contain disulfide bonds. However, the increased activity of CjCST-I cannot be explained by the general chaperone activity of DsbC.

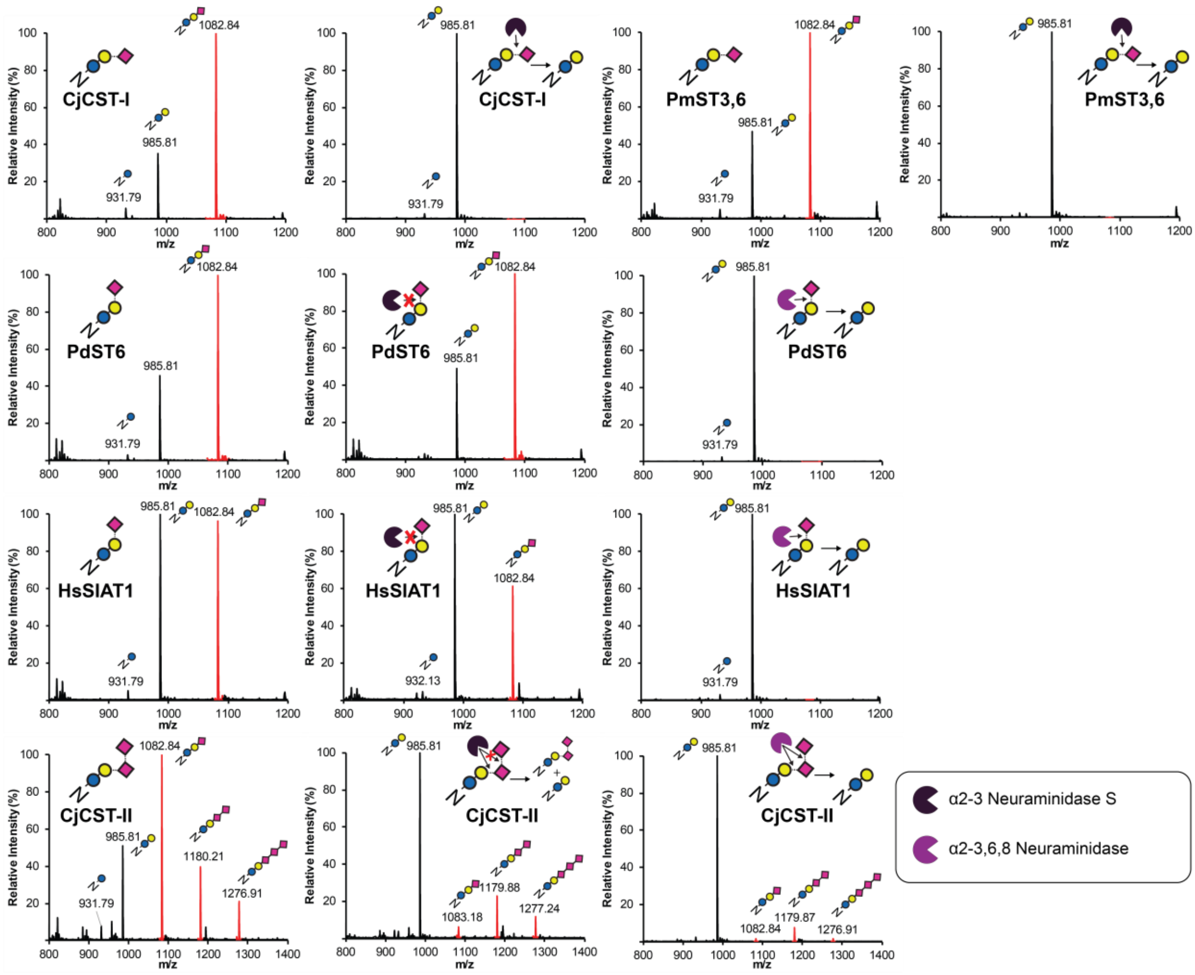

Supplementary Figure 10: Exoglycosidase sequencing of Im7-6 modified by GlycoPRIME biosynthetic pathways containing sialic acids. Completed IVG reactions from the GlycoPRIME workflow where purified using Ni-NTA magnetic beads, incubated at 37°C for at least 4 h with and without indicated commercially available exoglycosidases, trypsinized overnight, and then analyzed by glycopeptide LC-MS. The α2-3 Neuraminidase S was able to remove the sialic acids installed by CjCST-I; PmST3,6; and the first sialic acid installed by CjCST-II, indicating that these enzymes were installed sialic acids with α2-3 linkages. Sialic acids installed by PdST6, HsSIAT1, as well as the second and third sialic acids installed by CjCST-II were resistant to digestion by α2-3 Neuraminidase S but were susceptible to cleavage by an α2-3,6,8 Neuraminidase which is consistent with the established α2-6 activity of PdST6 and HsSIAT1 and the α2,8 linkages installed by CjCST-II in subsequent sialic acid additions. See **Methods** section for exoglycosidase details. All spectra were acquired from full elution peak areas of all detected glycosylated and aglycosylated species of the Im7-6 tryptic peptide EATTGGNWTTAGGDVLDVLLEHFVK containing an ApNGT glycosylation acceptor sequence. All indicated glycopeptide products are triply charged ions consistent with this Im7-6 tryptic peptide modified with indicated sugar structures.

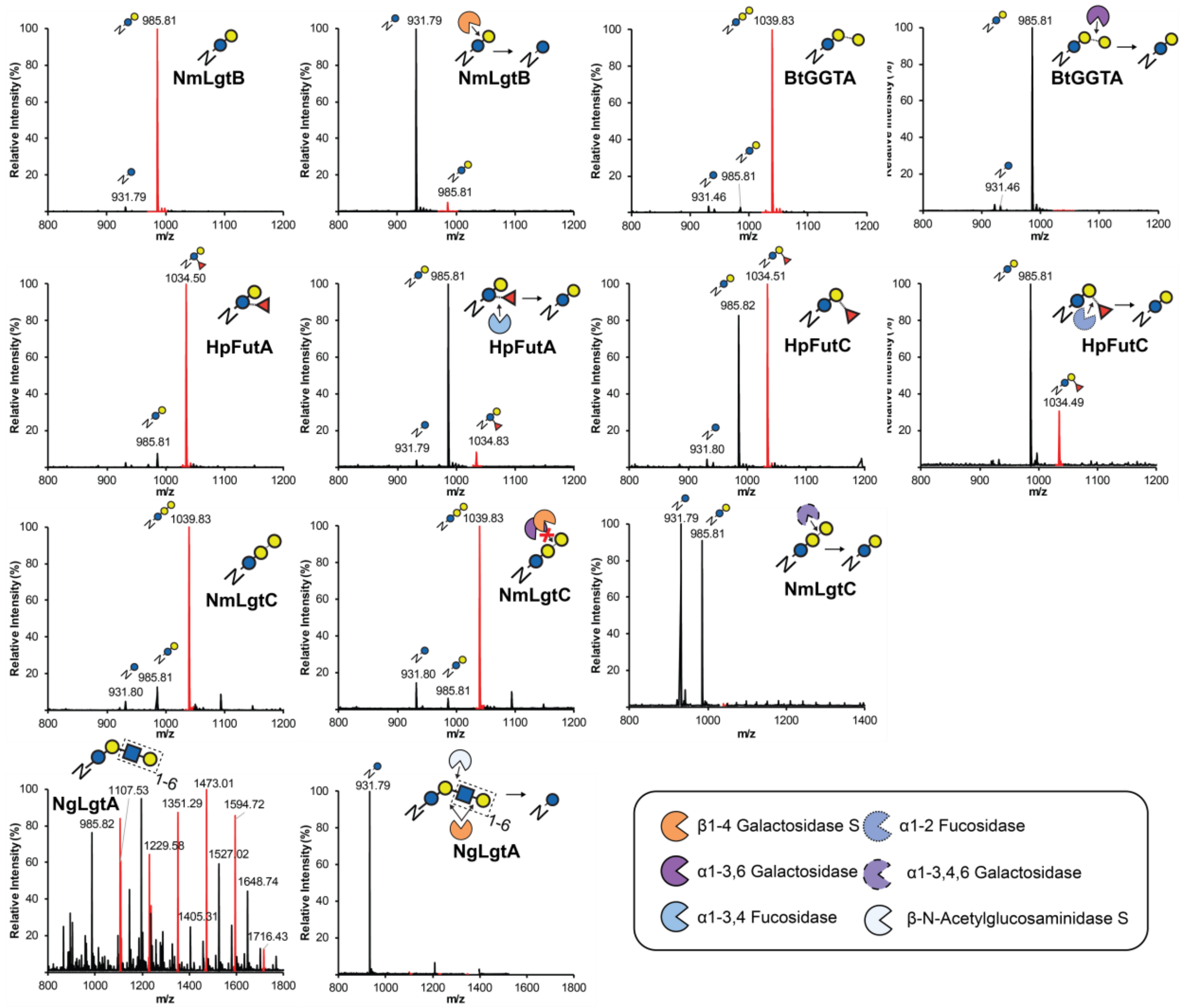

Supplementary Figure 11: Exoglycosidase sequencing of Im7-6 modified by GlycoPRIME biosynthetic pathways not containing sialic acids. Completed IVG reactions from the GlycoPRIME workflow where purified using Ni-NTA magnetic beads, incubated at 37°C for at least 4 h with and without indicated commercially available exoglycosidases, trypsinized overnight, and then analyzed by glycopeptide LC-MS. The sugars installed by NmLgtB, BtGGTA, HpFutA, and HpFutC were susceptible to cleavage by commercially available β1-4 Galactosidase S; α1-3,6 Galactosidase; α1-3,4 Fucosidase; and α1-2 Fucosidase, respectfully. The galactose installed by NmLgtC was resistant to cleavage by β1-4 Galactosidase S and α1-3,6 Galactosidase, but susceptible to cleavage by α1-3,4,6 Galactosidase. The LacNAc polymer installed by alternating activities by NmLgtB and NgLgtA was susceptible to cleavage by a mixture of β1-4 Galactosidase S and the β-*N*-Acetylglucosaminidase S. All spectra were acquired from full elution peak areas of all detected glycosylated and aglycosylated species of the Im7-6 tryptic peptide EATTGGNWTTAGGDVLDVLLEHFVK containing an ApNGT glycosylation acceptor sequence. All indicated glycopeptide products are triply charged ions consistent with this Im7-6 tryptic peptide modified with indicated sugar structures. Cleavage observations are consistent with previously established GT activities (**Figs. 2-3 and Supplementary Table 4**). See **Methods** section for exoglycosidase details.

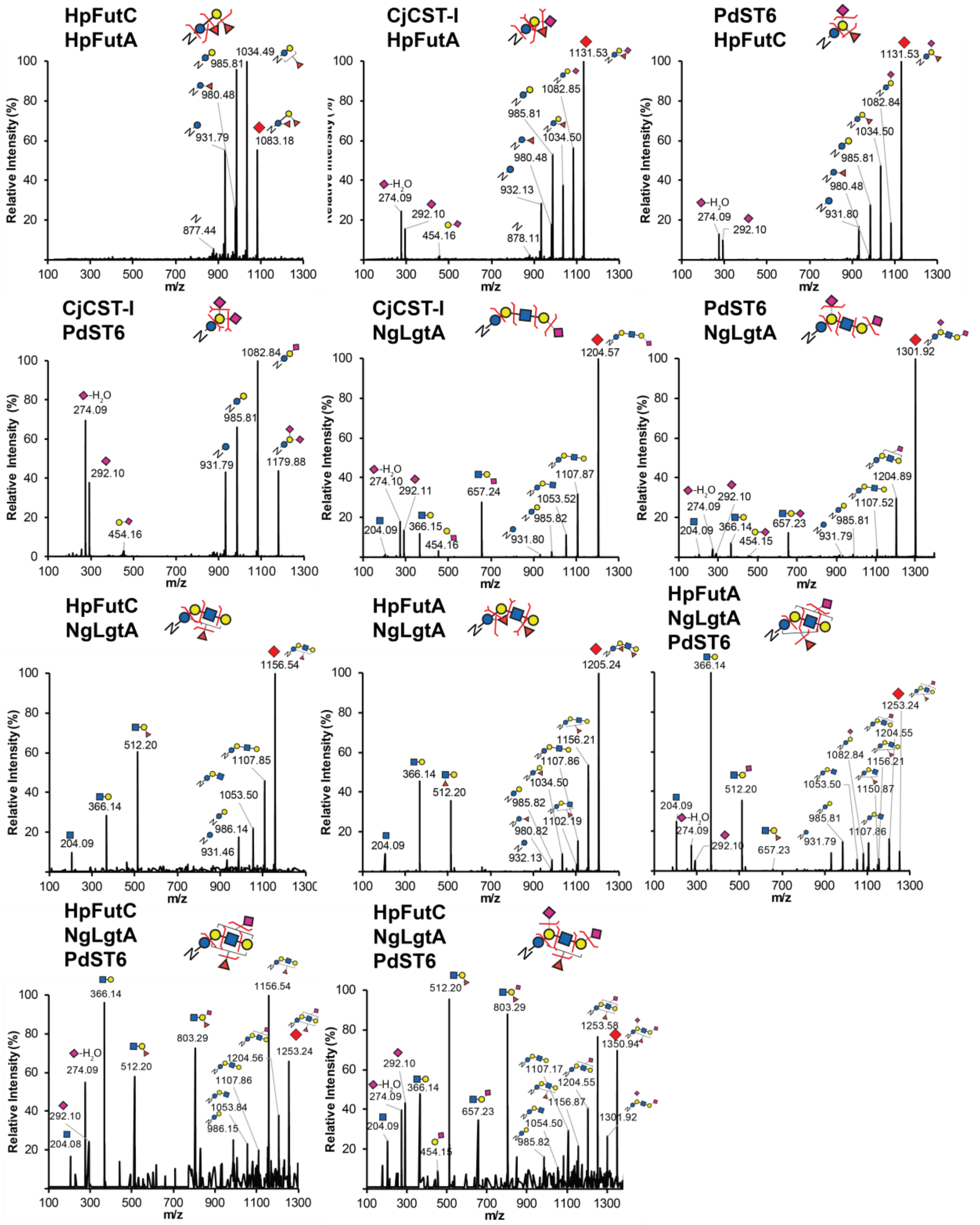

Supplementary Figure 12: Glycopeptide MS/MS spectra of GlycoPRIME reaction products from four and five enzyme biosynthetic pathways elaborating *N*-linked lactose. Products from IVG reactions containing four and five enzyme pathways modifying Im7-6 shown in **Fig. 3d and Supplementary Fig. 14** were purified, trypsinized, and analyzed by pseudo MRM MS/MS fragmentation at theoretical glycopeptide masses (indicated by red

diamonds) corresponding to detected protein MS peaks in **Fig. 3d and Supplementary Fig. 14**. All glycopeptides were fragmented using a collisional energy of 30 eV with a window of ± 2 m/z from targeted m/z values (see **Methods**). Spectra representative of many MS/MS acquisitions from n=1 IVG reaction. Theoretical protein, peptide, and sugar ion masses derived from expected glycosylation structures are shown in **Supplementary Tables 3 and 5**. All indicated sugar ions are singly charged and glycopeptide fragmentation products are triply charged ions consistent with modification of Im7-6 tryptic peptide EATTGGNWTTAGGDVLDVLLEHFVK with indicated sugar structures. Predicted sugar linkages based on previously established GT activities (**Supplementary Table 4**). Although products from five-enzyme biosynthetic pathway product could not be unambiguous defined, sugar and glycopeptide fragments do suggest modification with both fucose and sialic acids. All IVG reactions contained Im7-6, ApNGT, NmLgtB, indicated enzymes, and appropriate sugar donors according to established GT activities.

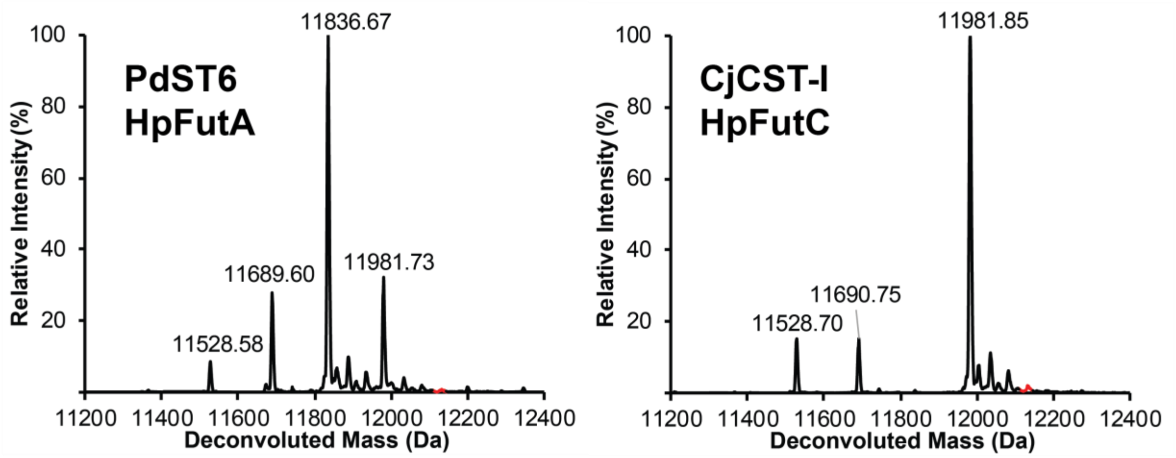

Supplementary Figure 13: Deconvoluted intact protein MS spectra of IVG reaction products showing no production fucosylated and sialylated species. Products of IVG reactions containing 10 μM Im7-6, 0.4 μM ApNGT, 2 μM NmLgtB, indicated enzymes, and 2.5 mM of appropriate sugar donors (UDP-Glc, UDP-Gal, CMP-Sia, and GDP-Fuc) were purified and analyzed by intact protein MS. Reactions contained 2.4 μM HpFutA and 2.4 μM PdST6 or 1.3 μM HpFutC and 0.65 μM CjCST-I as indicated. Deconvoluted spectra representative of n=2 IVGs. No peaks were detected that indicated the presence of Im7-6 modified with both a sialic acid and a fucose (the region of the spectra annotated in red line shows expected range of sialylated and fucosylated species) (see **Supplementary Table 4** for theoretical mass values).

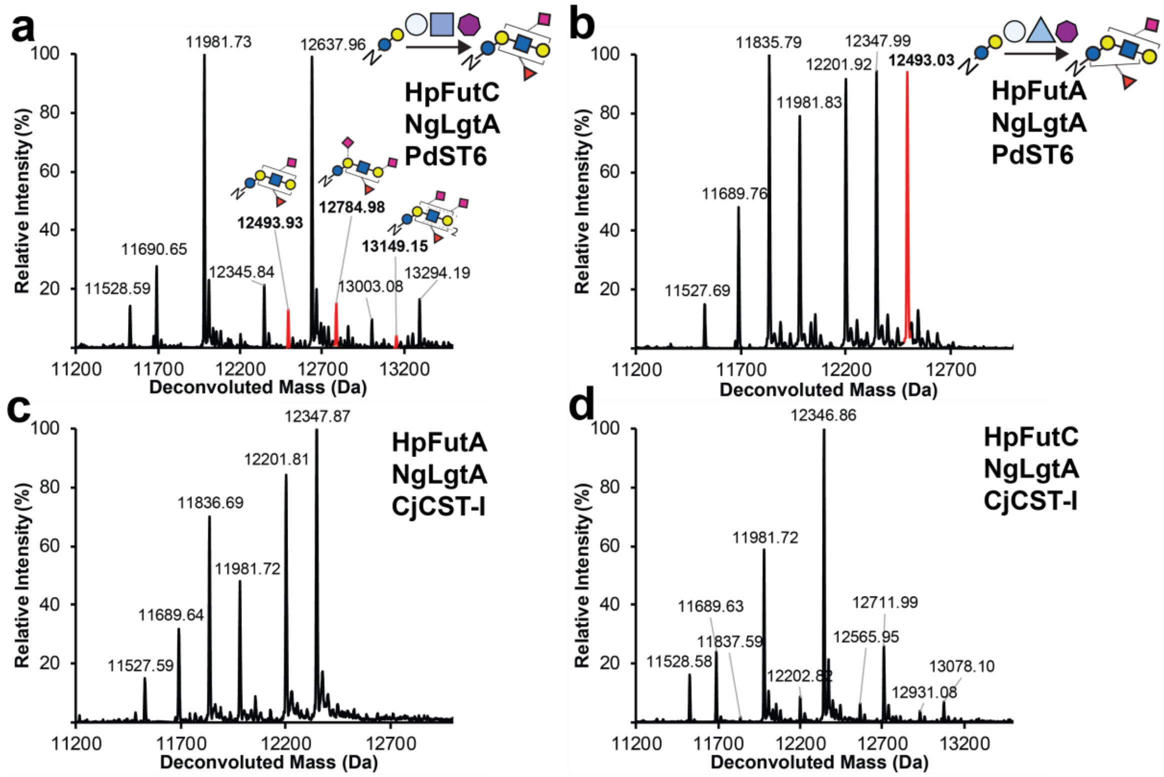

Supplementary Figure 14: GlycoPRIME screening of biosynthetic pathways containing five enzymes. Products of IVG reactions containing 10 μM Im7-6, 0.4 μM ApNGT, 2 μM NmLgtB, indicated GTs, and 2.5 mM of appropriate sugar donors (UDP-Glc, UDP-Gal, CMP-Sia, and GDP-Fuc) were purified from and analyzed by intact protein MS. Deconvoluted spectra representative of n=2 IVGs. **(a)** Deconvoluted intact protein MS of IVG reactions containing 0.87 μM HpFutC, 3.83 μM NgLgtA, and 1.63 μM PdST6. **(b)** Deconvoluted intact protein MS of IVG reactions containing 1.63 μM HpFutA, 3.83 μM NgLgtA, and 1.63 μM PdST6 (also shown in **Fig. 3d**) **(c)** Deconvoluted intact protein MS of IVG reactions containing 1.63 μM HpFutA, 3.83 μM NgLgtA, and 0.43 μM CjCST-I. **(d)** Deconvoluted intact protein MS of IVG reactions containing 0.87 μM HpFutC, 3.83 μM NgLgtA, and 0.43 μM CjCST-I. Spectra in **a** and **b** as well as fragmentation spectra in **Supplementary Fig. 12** indicated three and one species, respectively, which contained both sialic acid and fucose. Predicted glycosylation structures based on previously established GT activities (**Supplementary Table 4**) and fragmentation spectra (**Supplementary Fig. 12**). Although structures cannot be unambiguously identified, the previously observed incompatibility of HpFutA and PdST6 as well as the presence of a 1083 m/z peak (Siaα6Galβ4Glc-Peptide) and the absence of a 1034 m/z (Galβ4(Fucα3)Glc-Peptide) peak in fragmentation spectra in **Supplementary Fig. 12** suggests that in **b** the proximal galactose is modified with a sialic acid while the GlcNAc is modified with the fucose. No peaks in **c** or **d** were detected that indicated the presence of Im7-6 modified with both a sialic acid and a fucose (see **Supplementary Table 3** for theoretical mass values).

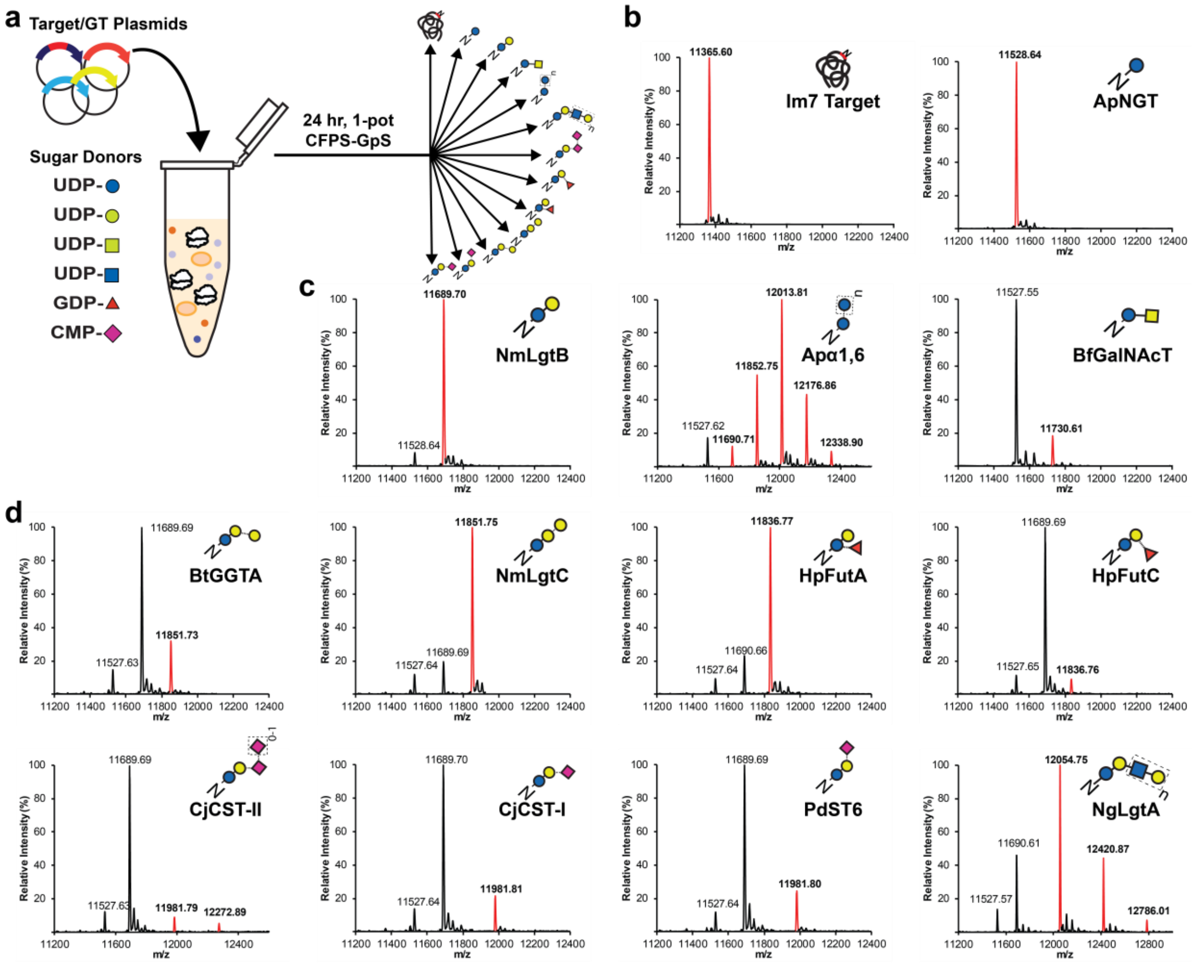

Supplementary Figure 15: Intact protein MS spectra of Im7-6 synthesized and glycosylated by CFPS-GpS reactions. (**a**) Plasmids encoding the Im7-6 target protein and sets of up to three GTs based on 12 successful biosynthetic pathways developed by two-pot GlycoPRIME screening were combined with appropriate sugar donors in one-pot CFPS-GpS reactions and incubated for 24 h at 30°C. (**b**) Deconvoluted intact protein spectra from Im7-6 synthesized and glycosylated in CFPS-GpS reactions with and without ApNGT plasmid. (**c**) Deconvoluted intact protein spectra from Im7-6 synthesized and glycosylated in CFPS-GpS reactions with ApNGT plasmid and indicated GT plasmids. (**d**) Deconvoluted intact protein spectra from Im7-6 synthesized and glycosylated in CFPS-GpS reactions with ApNGT, NmLgtB, and indicated GT plasmids. All reactions contained equimolar amounts of each plasmid and a total plasmid concentration of 10 nM. All Im7-6 proteins were purified using Ni-NTA magnetic beads before intact protein analysis (see **Methods**). All reactions showed intact protein mass shifts consistent with the modification of Im7-6 with the same glycans observed in our two-pot system (**Figs. 2-3**), although at lower efficiency. MS spectra were acquired from full elution areas of all detected glycosylated and aglycosylated protein or peptide species and are representative of n=2 CFPS-GpS reactions. Deconvoluted spectra collected from m/z 100-2000 into 11,000-14,000 Da using Bruker Compass Data Analysis maximum entropy method. See **Supplementary Fig. 5** for theoretical mass values.

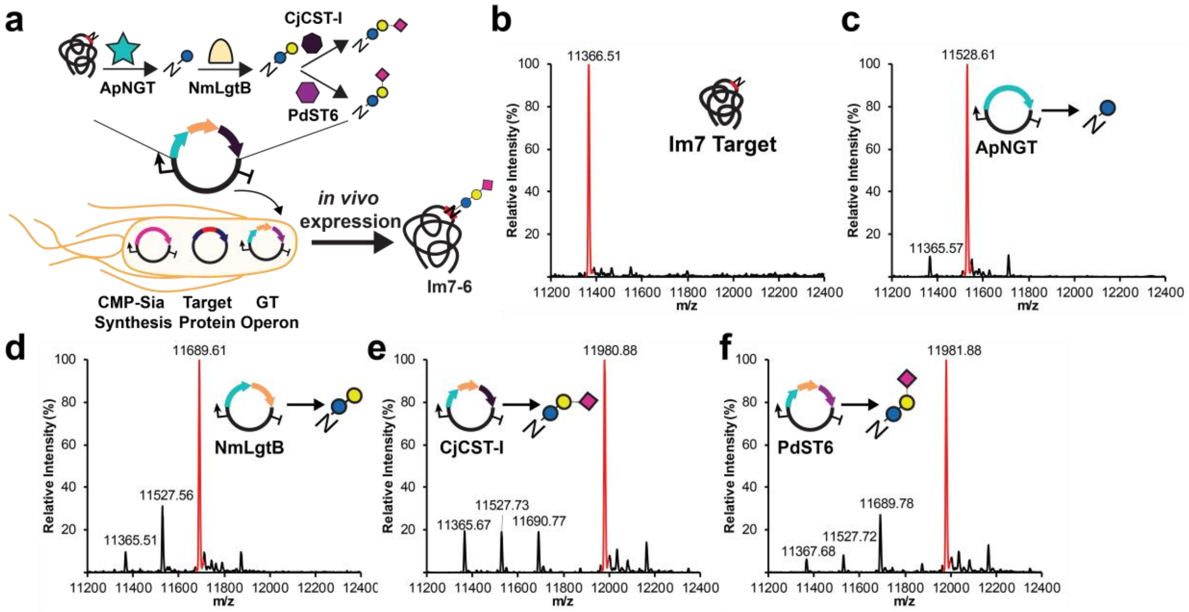

Supplementary Figure 16: Production of sialylated Im7-6 in the *E. coli* cytoplasm. (**a**) Design of cytoplasmic glycosylation system to produce sialylated glycoproteins in *E. coli*. Three plasmids containing NmNeuA (CMP-Sia synthesis), target protein containing ApNGT glycosylation acceptor sequence, and biosynthetic pathways discovered using GlycoPRIME (GT operon). (**b-f**) Deconvoluted intact protein spectra from Im7-6 purified from CLM24Δ*nanA* *E. coli* strain containing CMP-Sia synthesis plasmid and Im7-6 target protein plasmid as well as no GT operon **b**; GT operon containing ApNGT **c**; GT operon containing ApNGT and LgtB **d**; GT operon containing ApNGT, NmLgtB, and CjCST-I **e**; or GT operon containing ApNGT, NmLgtB, and PdST6 **f**. The last GT in all glycosylation pathways is indicated. Mass shifts in intact protein spectra are consistent with established activities of each GT and the installation of *N*-linked Glc, lactose, 3'-sialyllactose, and 6'-sialyllactose onto Im7-6 in **b, c, d, e, and f**, respectively. All *E. coli* cultures were supplemented with 5 mM sialic acid and grown to OD600 = 0.6 at 37°C, induced with 1 mM IPTG and 0.2% arabinose, and then incubated overnight at 25°C. MS spectra were acquired from full elution areas of all detected glycosylated and aglycosylated protein species and were deconvoluted from m/z 100-2000 into 11,000-14,000 Da using Bruker Compass Data Analysis maximum entropy method. See **Supplementary Table 3** for theoretical masses. Spectra representative of n=2 bacterial cultures.

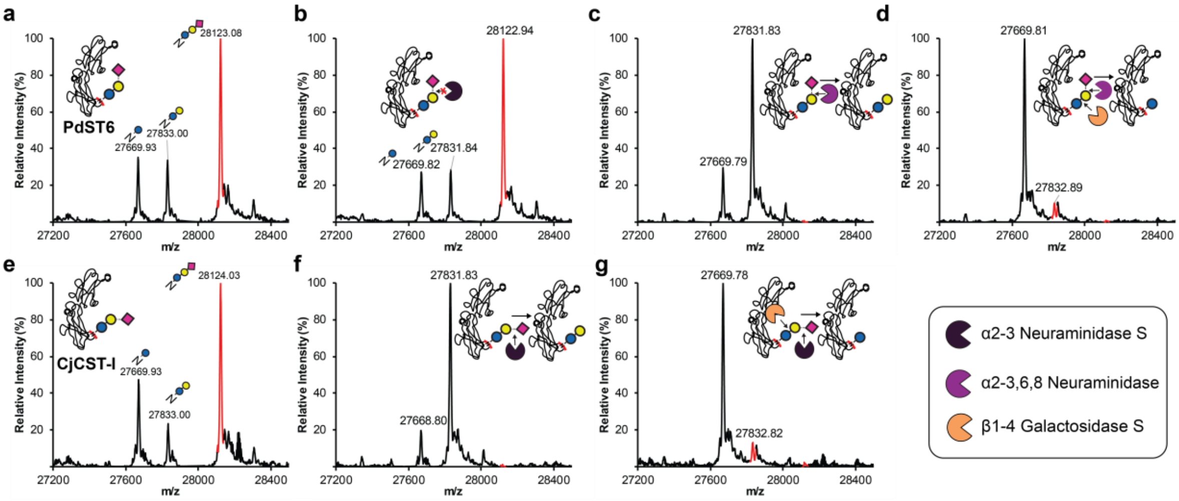

Supplementary Figure 17: Exoglycosidase sequencing of Fc glycosylated in the *E. coli* cytoplasm. (**a**) Deconvoluted intact protein spectra from Fc-6 purified from CLM24Δ*nanA E. coli* strain containing CMP-Sia synthesis plasmid, Fc-6 target protein plasmid, and a GT operon plasmid containing ApNGT, NmLgtB, and PdST6. (**b-d**) Purified Fc-6 from **a** was incubated at 37°C for at least 4 h with commercially available α2-3 Neuraminidase S **b**, α2-3,6,8 Neuraminidase **c**, or β1-4 Galactosidase S and α2-3,6,8 Neuraminidase **d**. Resistance of terminal sialic acid to α2-3 Neuraminidase S and susceptibility to α2-3,6,8 Neuraminidase indicates an α2-6 linkage, which is consistent with previously established activity of PdST6 (**Supplementary Table 4**). (**e**) Deconvoluted intact protein spectra from Fc-6 purified from CLM24Δ*nanA E. coli* strain containing CMP-Sia synthesis plasmid, Fc-6 target protein plasmid, and a GT operon plasmid containing ApNGT, NmLgtB, and CjCST-I. (**f-g**) Purified Fc-6 from **e** was incubated at 37°C for at least 4 h with commercially available α2-3 Neuraminidase S **b**, or β1-4 Galactosidase S and α2-3 Neuraminidase S. Susceptibility of terminal sialic acid to α2-3 Neuraminidase confirms the previously established activity of CjCST-I (**Supplementary Table 4**). Removal of middle galactose with addition β1-4 Galactosidase S in **d** and **g** confirms the previously established activity of NmLgtB (**Supplementary Table 4**). **a-c** and **e-f** are also shown in **Fig. 4.** See **Methods** for exoglycosidase details and **Supplementary Table 7** for theoretical glycoprotein masses. All *E. coli* cultures were supplemented with 5 mM sialic acid and grown to OD600 = 0.6 at 37°C then induced with 1 mM IPTG and 0.2% arabinose then incubated overnight at 25°C. MS spectra were acquired from full elution areas of all detected glycosylated and aglycosylated protein species and were deconvoluted from m/z 100-2000 into 27,000-29,000 Da using Bruker Compass Data Analysis maximum entropy method.

Supplementary Note 1: DNA sequences encoding engineered glycosylation targets, *in vitro* expressed glycosyltranferases, *in vivo* glycosyltranferases operons, and *in vivo* CMP-Sia production plasmid.

Key:
TRANSLATED REGION
Engineered glycosylation acceptor sequence
Flanking regions adjacent to glycosylation acceptor sequence
untranslated region
promoter
terminator
AFFINITY TAG OR CSL LEADING SEQUENCE

DNA sequence for Im7-6 Variant in pJL1 plasmid context:
gaaattaatacgactcactatagggagaccacaacggtttccctctagaaataattttgtttaactttaagaaggagatatacat
ATGGAACTGGAAAATAGTATTAGTGATTACACAGAGGCTGAGTTTGTTCAACTTCTTAAGG
AAATTGAAAAAGAGGCGACTACC**GGAGGTAACTGGACAACA**GCGGGAGGAGATGTGTT
AGATGTGTTACTCGAACACTTTGTAAAAATTACTGAGCATCCAGATGGAACGGATCTGAT
CTATTATCCTAGTGATAATAGAGACGATAGCCCCGAAGGGATTGTCAAGGAAATTAAAGAA
TGGCGAGCTGCTAACGGTAAGCCAGGATTTAAACAGGGCGGATCCCATCACCATCATCA
CCATTAAgtcgaccggctgctaacaaagcccgaaaggaagctgagttggctgctgccaccgctgagcaataactagcata
accccttggggcctctaaacgggtcttgaggggttttttgctgaaag

DNA Sequence of ApNGT in pJL1 Context:
gaaattaatacgactcactatagggagaccacaacggtttccctctagaaataattttgtttaactttaagaaggagatatacatAT
GGAAAACGAGAATAAACCGAACGTGGCAAATTTTGAAGCAGCAGTTGCAGCCAAAGATT
ATGAAAAAGCATGTAGCGAGCTGCTGCTGATTCTGAGCCAGCTGGATAGCAATTTTGGT
GGCATTCATGAAATCGAGTTCGAGTACCCAGCTCAGCTGCAGGATCTGGAGCAGGAAAA
AATTGTGTACTTTTGCACCCGTATGGCGACTGCCATCACCACCCTGTTCTCTGACCCGG
TTCTGGAAATCTCCGACCTGGGTGTGCAGCGTTTCCTGGTTTATCAGCGTTGGCTGGCG
CTGATCTTCGCTAGCAGCCCGTTCGTGAACGCAGACCACATCCTGCAGACTTACAACCG
TGAACCGAACCGCAAAAACTCCCTGGAAATTCACCTGGACTCTAGCAAGTCCTCTCTGA
TTAAATTTTGCATCCTGTACCTGCCGGAATCTAATGTGAACCTGAATCTGGATGTGATGTG
GAACATTTCCCCGGAGCTGTGTGCCTCTCTGTGCTTTGCGCTGCAAAGCCCGCGTTTT
GTTGGCACCAGCACCGCCTTTAACAAACGCGCGACCATTCTGCAGTGGTTCCCGCGTC
ATCTGGACCAGCTGAAAAACCTGAACAACATCCCGTCCGCTATCTCTCATGACGTGTATA
TGCACTGTTCTTACGACACCAGCGTTAACAAGCACGATGTTAAGCGCGCGCTGAATCAC
GTGATTCGTCGCCACATCGAATCCGAATACGGTTGGAAAGATCGTGATGTGGCTCACAT
CGGTTATCGCAACAACAAACCGGTTATGGTCGTTCTGCTGGAACATTTTCATAGCGCGCA
CTCTATCTACCGTACTCACTCTACCAGCATGATCGCCGCGCGCGAACACTTCTATCTGAT
CGGCCTGGGTTCCCCGAGCGTTGACCAGGCCGGTCAGGAGGTTTTCGATGAATTCCAC
CTGGTAGCGGGTGACAACATGAAGCAAAAACTGGAATTCATTCGTTCTGTGTGCGAAAG
CAATGGTGCGGCAATTTTCTACATGCCGAGCATCGGTATGGATATGACCACCATCTTCGC

GTCCAATACCCGTCTGGCGCCGATTCAGGCAATCGCCCTGGGCCACCCGGCGACTACT
CACTCCGACTTCATTGAATACGTTATCGTGGAAGACGACTACGTCGGCTCTGAGGAATG
CTTCTCTGAAACCCTGCTGCGTCTGCCGAAAGACGCTCTGCCGTATGTTCCGTCCGCC
CTGGCTCCGGAGAAAGTTGATTACCTGCTGCGTGAAAACCCTGAAGTTGTCAACATCGG
TATTGCCTCTACCACTATGAAGCTGAACCCGTACTTCCTGGAAGCACTGAAGGCCATTCG
TGACCGTGCGAAGGTGAAAGTGCACTTCCACTTCGCACTGGGCCAGTCCAATGGTATC
ACTCACCCTTACGTTGAACGCTTTATCAAATCTTACCTGGGCGACAGCGCTACCGCGCA
CCCGCACTCTCCGTACCACCAGTACCTGCGTATTCTGCACAACTGCGATATGATGGTAAA
CCCTTTTCCGTTTGGTAATACCAATGGTATTATTGACATGGTAACCCTGGGTCTGGTAGGT
GTTTGCAAAACCGGTGCGGAAGTCCACGAACATATCGATGAAGGCCTGTTCAAACGTCT
GGGCCTGCCGGAATGGCTGATTGCAAACACCGTGGACGAATACGTGGAACGTGCAGTG
CGCCTGGCCGAGAACCATCAGGAACGTCTGGAACTGCGTCGTTACATTATTGAAAACAA
TGGCCTGAACACCCTGTTCACCGGCGACCCACGCCCGATGGGTCAGGTGTTCCTGGAA
AAACTGAACGCATTCCTGAAGGAAAACTAAgtcgaccggctgctaacaaagcccgaaaggaagctgagtt
ggctgctgccaccgctgagcaataactagcataaccccttggggcctctaaacgggtcttgaggggttttttgctgaaa

DNA Sequence of Apα1-6 in pJL1 Context:
gaaattaatacgactcactatagggagaccacaacggtttccctctagaaacgacactatactacttaaggaggctaattATGG
AAAACAACATCGACCTGAACGTTTATTTCTGCTTCGTCAACCGTCCATGCACTGGCGGC
GATTTCGTTAACCTGGATCACGTCCGTACCCTGCGCAAACTGGGCATCAACGCTAGCAT
TCTGCTGGCTGGCAACCAGTCCGAAGAAATCGTTAACAGCTTCGGCTCTCTGCCAGTTG
TGATTCTGAACGAAGAGATTGAGTTTAGCTCCCAGGATATCTTCATTGTGCCGGAAGTTA
TGCAGGTTCTGTACGATCTGGCTTCCAAGATGACCGTCTTCCCGCGTATGATTATGCACA
ACCAAAACCCATTTTACACTGGCTATGGTTTCCTGTCCGCGCAGCACATTAACGAACACC
GTCTGGAACGCATTATCGTCCCGTCCAGCTACACCAAATACAAACTGCAGGAAATCGGC
GTAACCAAACCGATCGATATCATTCATCCGTATATTCCAGATTATTTCAAGCCGGCGGAAA
AACAGCGTGAGGTCATTCAAATCGCCTTCTCCCGTCGTAAACGTTCTGCGGAATTCGAC
ATCTTCAAGTTCTACTTCCTGTCCCTGTACTCCCACAAACACTCTGTAAACTTTGTTAACA
TCCAGGGTCTGACCCGCGAGGAAGTGGCGAAGGTTATGTCTGAAGCGGCCATCTTTATT
TCCTTTGCTGAACGTGAATCTCTGGGTCTGATGACGCTGGAAGCTATGGCATCCGGTTG
TCACGTTATCGGCTTCTCCGGTTATACTGACATCTACAATAACGAAGTTATTGACGATTCT
GTTGGTGACTGGATCGGTGAAGGCGAGTACACCCTGTTCGCACAGAAAGTTTGTCAGG
CGATCGATGACTTCGTGAACGGCAAAATGAATCCGAAAATTGAAAACGGTCTGCGTCTG
ATCGAACAGCGTTTCCGTATTCGTCACTTCGAACAGGAAGTAAAACGTGTATACGGTAAC
ATTTTCGATTATGATCTGGAAAACTCTCGCTCCTAAgtcgaccggctgctaacaaagcccgaaaggaag
ctgagttggctgctgccaccgctgagcaataactagcataaccccttggggcctctaaacgggtcttgaggggttttttgctgaaa

DNA Sequence of BfGalNAcT1 in pJL1 Context:
gaaattaatacgactcactatagggagaccacaacggtttccctctagaaaaggagactagacttattaatttaaggaggctata
ATGGGTACCAACAACTCTGACTTCTACCTGCCGGTCTATGTTATTAATCTGAAAGAACGTA
CTGAACGCCGTCAGCACATTGAGGAGCAGTTCCAAGGTAAAGTTGAATTCGCTCTGCAC
TGGATCGAGGCGATCGAGCACAGCATCGGTGCGGTGGGTCTGTGGCAGTCTATGCTGA
AAGCCGTTCAGACTGCTATCGACAAACGCGACGATATCATGATCATTTGTGAGGATGACC

ACATTTTCACTCCGGCATACAACAAGGACTACCTGTTCGCGAATATCATCGGTGCGAACG
CACAGGGTAGCGAACTGCTGTCCGGTGGTGTTGGCGGTTTCGGTACCGCGGTGCCAG
TGGATACCAACCGTTATTGGATGGACTGGTTCTGGTCTACCCAATTCATCATCATCTTCAA
GCCGCTGTTCCAGAAAATCCTGGACTATGATTTCAAAGACACCGACACGGCTGACGGC
GTCCTGAGCGTTCTGGCGAAAGACAAGATGACCATCTATCCGTTCATTAGCGTTCAAAAA
GATTTCGGTTACAGCGACGTGACCGTTTACAACGGTACTCCTGGTATGATCTCTAACTAC
TTTTCCCAGGCTAACTACCGCCTGCGTATGATCCACCATGTAAGCCACAAATTCAAAGAG
CAGGCAAAACGCTAAgtcgaccggctgctaacaaagcccgaaaggaagctgagttggctgctgccaccgctgagca
ataactagcataaccccttggggcctctaaacgggtcttgaggggttttttgctgaaag

DNA Sequence of Hpβ4GalT in pJL1 Context:
gaaattaatacgactcactatagggagaccacaacggtttccctctagaaataattttgtttaactttaagaaggagatatacatAT
GGAGAAAAAAATCTGGAGCCATCCGCAGTTCGAAAAAGGCTCCCGCGTGTTTATTATCA
GCCTGAATCAGAAAGTTTGCGATCAGTTTGGTCTGGTTTTTCGTGATACCACCACCCTG
CTGCATAACATTAATGCAACCCATCATAAAGCCCAGATCTTCGATGCAATTTACAGCAAAA
CCTTTGAAAGCGAACTGCATCCGCTGGTTAAAAAACATCTGCATCCGTATTTTATCACCC
AGAACATTAAAGATATGGGCATTACCACCAATCTGATTAGCCGTGTTAGCAAATTCTATTAT
GCCCTGAAATACCATGCCAAATTTATGAGCTTTGGTGAACTGGGTTGTTATGCAAGCCAT
TATAGCCTGTGGGAAAAATGCATTGAACTGAATGAACCGATTTGCATCCTGGAAGATGAT
ATCACCCTGAAAGAAGATTTTAAAGAGGGCCTGGATTTCCTGGAAAAACATATTCAAGAA
CTGGGCTATGCACGTCTGATGTATCTGCTGTATGATGCCAATGTTAAAAGCGAACCGCTG
AGCCATAAAAACCATGAAATCCAAGAACGTGTGGGCATCATTAAAGCATATAGCCATGGT
GTTGGCACCCAGGGTTATGTTATTACCCCGAAAATTGCCAAAGTGTTCAAAAAATGTAGC
CGCAAATGGGTTGTTCCGGTTGATACCATTATGGATGCAACCTTTATTCACGGCGTTAAA
AATCTGGTTCTGCAGCCGTTTGTTATTGCAGATGATGAGCAGATTAGCACCATTGCACGT
AAAGAAGAACCGTATAGCAGCAAAATTGCACTGATGCGTAAACTGCACTTCAAATATCTG
AAATACTGGCAGTTCGTGTAAgtcgaccggctgctaacaaagcccgaaaggaagctgagttggctgctgccacc
gctgagcaataactagcataaccccttggggcctctaaacgggtcttgaggggttttttgctgaaag

DNA Sequence of NmLgtB in pJL1 Context:
gaaattaatacgactcactatagggagaccacaacggtttccctctagaaataattttgtttaactttaagaaggagatatacatAT
GCAGAACCATGTTATTAGCCTGGCAAGCGCAGCAGAACGTCGTGCACATATTGCAGATA
CCTTTGGTCGTCATGGTATTCCGTTTCAGTTTTTTGATGCACTGATGCCGAGCGAACGTC
TGGAACAGGCAATGGCAGAACTGGTTCCGGGTCTGAGCGCACATCCGTATCTGAGCGG
TGTTGAAAAAGCATGTTTTATGAGCCATGCAGTTCTGTGGAAACAGGCACTGGATGAAG
GTCTGCCGTATATTACCGTTTTTGAAGATGATGTTCTGCTGGGTGAAGGTGCAGAAAAAT
TTCTGGCAGAAGATGCCTGGCTGCAAGAACGTTTTGATCCGGATACCGCATTTATTGTTC
GTCTGGAAACCATGTTTATGCATGTTCTGACCAGCCCGAGCGGTGTGGCAGATTATTGT
GGTCGTGCATTTCCGCTGCTGGAAAGCGAACATTGGGGCACCGCAGGTTATATCATTAG
CCGTAAAGCAATGCGCTTTTTTCTGGATCGTTTTGCAGCACTGCCTCCGGAAGGCCTGC
ATCCGGTTGATCTGATGATGTTTAGCGATTTTTTTGATCGTGAAGGTATGCCGGTTTGTCA
GCTGAATCCGGCACTGTGTGCACAAGAACTGCACTATGCAAAATTTCATGATCAGAATAG
CGCACTGGGTAGCCTGATTGAACATGATCGTCTGCTGAATCGTAAACAGCAGCGTCGTG

ATAGTCCGGCAAATACCTTTAAACATCGTCTGATTCGTGCCCTGACCAAAATTAGCCGTG
AACGTGAAAAACGTCGTCAGCGTCGCGAACAGTTTATTGTGCCGTTTCAGGGATCCTGG
AGCCATCCGCAGTTCGAAAAATAAgtcgaccggctgctaacaaagcccgaaaggaagctgagttggctgctgc
caccgctgagcaataactagcataaccccttggggcctctaaacgggtcttgaggggttttttgctgaaag

DNA Sequence of Btβ4GalT1 in pJL1 Context:
gaaattaatacgactcactataggggagaccacaacggtttccctctagaacgatatcgtcacactagttaaggaggttaagaAT
GAAATTCCGTGAGCCGCTGCTGGGCGGCTCTGCTGCAATGCCGGGCGCCTCTCTGCAA
CGTGCATGCCGTCTGCTGGTCGCAGTTTGCGCGCTGCACCTGGGTGTTACCCTGGTCT
ACTATCTGGCTGGCCGCGATCTGCGTCGCCTGCCGCAACTGGTTGGTGTGCACCCGCC
TCTGCAGGGTAGCAGCCATGGTGCGGCAGCTATCGGCCAACCGAGCGGTGAACTGCG
CCTGCGTGGTGTTGCACCGCCGCCTCCGCTGCAGAACAGCAGCAAACCGCGTTCTCG
CGCGCCGAGCAACCTGGACGCGTACTCCCACCCTGGCCCGGGCCCAGGTCCGGGCA
GCAATCTGACTTCTGCTCCTGTACCGTCTACCACCACCCGCAGCCTGACCGCATGCCC
GGAAGAATCTCCGCTGCTGGTTGGTCCGATGCTGATCGAATTCAACATCCCAGTTGACC
TGAAGCTGGTGGAACAGCAAAACCCTAAGGTGAAGCTGGGTGGTCGTTACACTCCAAT
GGATTGCATTTCTCCGCACAAAGTCGCAATTATTATCCCTTTCCGTAACCGTCAGGAACA
CCTGAAATACTGGCTGTACTACCTGCACCCGATCCTGCAGCGCCAGCAGCTGGATTACG
GTATCTACGTGATTAATCAGGCGGGTGAGAGCATGTTCAATCGCGCGAAGCTGCTGAAC
GTTGGTTTCAAGGAGGCTCTGAAAGACTACGACTACAACTGTTTCGTATTCTCTGATGTG
GACCTGATCCCGATGAACGACCACAACACCTACCGCTGCTTCTCCCAGCCGCGCCATAT
TTCTGTCGCAATGGATAAATTCGGTTTTAGCCTGCCATACGTCCAGTACTTCGGCGGCGT
TTCCGCTCTGAGCAAACAACAGTTCCTGTCTATCAACGGTTTTCCTAACAACTATTGGGG
CTGGGGTGGTGAAGATGACGATATTTACAACCGCCTGGCGTTTCGTGGTATGTCCGTTA
GCCGTCCGAACGCGGTTATCGGTAAATGCCGCATGATCCGCCATTCTCGTGATAAGAAG
AACGAGCCGAATCCGCAGCGCTTCGACCGTATCGCCCACACCAAAGAAACTATGCTGTC
CGACGGTCTGAATTCCCTGACTTACATGGTACTGGAAGTACAGCGTTATCCGCTGTATAC
CAAAATCACCGTTGATATCGGCACTCCGTCTTAAgtcgaccggctgctaacaaagcccgaaaggaagct
gagttggctgctgccaccgctgagcaataactagcataaccccttggggcctctaaacgggtcttgaggggttttttgctgaaag

DNA Sequence of NgLgtB in pJL1 Context:
gaaattaatacgactcactataggggagaccacaacggtttccctctagaaataattttgtttaactttaagaaggagatatacat
ATGCAGAACCACGTGATTTCCCTGGCTTCAGCGGCCGAGCGCCGTGCTCATATTGCTGC
CACCTTTGGTAGTCGTGGAATCCCTTTCCAGTTCTTCGATGCCCTGATGCCTTCAGAAC
GTCTGGAGCAGGCAATGGCGGAGCTGGTCCCTGGTCTGTCAGCCCATCCTTATCTGTC
TGGCGTTGAAAAAGCGTGTTTCATGTCCCATGCTGTCCTGTGGGAACAAGCCCTGGATG
AGGGTCTGCCGTATATCGCCGTGTTTGAGGACGATGTGCTGCTGGGTGAAGGTGCTGA
ACAGTTTCTGGCCGAGGACACTTGGCTGGAAGAGCGTTTCGATAAAGACTCAGCGTTCA
TTGTCCGTCTGGAGACAATGTTTATGCACGTGCTGACTTCTCCATCTGGTGTAGCCGATT
ATGGCGGTCGTGCCTTTCCTCTGCTGGAGTCCGAACACTGTGGTACAGCCGGGTATATT
ATCAGCCGTAAAGCCATGCGTTTCTTTCTGGATCGTTTTGCTGTGCTGCCTCCGGAGCG
CCTGCATCCTGTTGATCTGATGATGTTTGGCAATCCTGATGACCGTGAGGGTATGCCAGT
TTGTCAGCTGAATCCGGCACTGTGTGCTCAGGAACTGCATTATGCCAAATTTCACGACC

AGAATAGCGCTCTGGGAAGTCTGATTGAACATGATCGTCGCCTGAACCGTAAACAACAG
TGGCGTGATAGTCCGGCTAACACGTTTAAACACCGCCTGATTCGTGCTCTGACCAAAATT
GGCCGTGAGCGTGAAAAACGTCGTAAACGCCGTGAACAGACGATTGGGAAAATCATTG
TGCCATTCCAGTGAgtcgaccggctgctaacaaagcccgaaaggaagctgagttggctgctgccaccgctgagcaat
aactagcataaccccttggggcctctaaacgggtcttgaggggttttttgctgaaag

DNA Sequence of SpWchJ in pJL1 Context:
gaaattaatacgactcactatagggagaccacaacggtttccctctagaaataattttgtttaactttaagaaggagatatacatAT
GAAAATCTGCCTGGTTGGTAGCAGCGGTGGTCATCTGACCCATCTGTATCTGCTGAAAC
CGTTTTGGAAAGATAAAGAACGTTTTTGGGTGACCTTCGATAAAGAAGATACCCGTAGCA
TTCTGGGCAACGAAACCTTTTATCCGTGTCATTATCCGACCAATCGCAATCTGAAAAACC
TGATTAAAAACACCGTTCTGGCCTTTAACATTCTGCGCAAAGAACGTCCGGATGTGATTA
TTAGCAGTGGTGCAGCAGTTGCAGTTCCGTTTTTCTATCTGGGTAAACTGTTTGGTGCCA
AAACCGTGTATATCGAAGTGTTTGATCGTATTGATGCACCGACCCTGACCGGTAAAATTG
TTTATCCGGTTACCGATAAATTCATCGTGCAGTGGGAAGAGATGAAAAAAGTTTATCCGA
AAGCCATTAATCTGGGTGGCATCTTTTAAgtcgaccggctgctaacaaagcccgaaaggaagctgagttggc
tgctgccaccgctgagcaataactagcataaccccttggggcctctaaacgggtcttgaggggttttttgctgaaag

DNA Sequence of SpWchK in pJL1 Context:
gaaattaatacgactcactatagggagaccacaacggtttccctctagaaataattttgtttaactttaagaaggagatatacatAT
GATCTTCGTTACCGTTGGCACCCATGAACAGCAGTTTAATCGTCTGATTAAAGAAGTGGA
TCGCCTGAAAGGTGAAGGCTTTATTCAGGATGATGTGTTTATTCAGACCGGCTATAGCAA
TTATGTGCCGAAATTTTGCAAATGGGAGAAACTGATCAGCTATGAAAAAATGAACCAGCT
GATCAAAGAGAGCGATATTATCATTACACATGGTGGTCCGGCAACCTTTATGGCAGTTATT
GCAAAAGGTAAAAACCCGATTATTGTGCCACGCCTGAAAAAATTCGGTGAACATGTTAAT
GATCATCAGATGCAGTTCGTGAAAATCACCAAAGAAATCTACAACCTGATCGTGATCGAT
GATATTAGCGATCTGCACCTGATTCTGCACAACTTCAAAGATAAACACTTCGAAACCTACC
TGAACAACGAACGTTTTAATGTGCGCTTTAACGTGGAAATCAGCAACCTGTTTAAAGGCA
ACAAAATCAATGAAAATTAAgtcgaccggctgctaacaaagcccgaaaggaagctgagttggctgctgccaccgct
gagcaataactagcataaccccttggggcctctaaacgggtcttgaggggttttttgctgaaag

DNA Sequence of NmLgtC in pJL1 Context:
gaaattaatacgactcactatagggagaccacaacggtttccctctagaagcacatagtacagacacagtataaggaggttcaa
tATGGATATCGTTTTCGCGGCAGACGATAACTACGCTGCCTACCTGTGCGTGGCAGCAAA
ATCCGTGGAAGCTGCCCACCCGGATACCGAAATCCGCTTCCACGTCCTGGACGCAGGT
ATTAGCGAAGCGAACCGTGCCGCAGTAGCGGCGAACCTGCGTGGCGGCGGCGGTAAC
ATCCGCTTCATCGATGTGAACCCGGAAGATTTTGCGGGCTTTCCACTGAACATCCGCCA
TATTTCCATCACTACTTATGCGCGTCTGAAACTGGGCGAATACATCGCGGATTGCGACAA
AGTTCTGTACCTGGATATCGATGTGCTGGTACGTGACTCTCTGACCCCGCTGTGGGACA
CTGACCTGGGCGATAACTGGCTGGGTGCCTGTATTGACCTGTTCGTGGAACGTCAGGA
AGGTTACAAACAAAAAATCGGTATGGCCGATGGCGAATACTACTTCAACGCTGGCGTGC
TGCTGATCAACCTGAAAAAATGGCGTCGTCATGATATTTTCAAAATGTCCTGCGAGTGGG
TAGAACAGTATAAGGACGTTATGCAGTACCAGGACCAGGATATCCTGAACGGCCTGTTTA

AAGGTGGTGTCTGTTACGCCAACAGCCGCTTCAACTTCATGCCGACCAACTACGCGTTC
ATGGCGAATCGCTTCGCGTCTCGTCACACCGACCCTCTGTACCGTGATCGCACCAACAC
CGTGATGCCGGTCGCGGTGTCCCACTACTGTGGTCCTGCGAAGCCATGGCACCGTGAC
TGTACCGCTTGGGGTGCTGAGCGTTTCACGGAACTGGCTGGTAGCCTGACCACGGTCC
CTGAAGAATGGCGCGGTAAACTGGCTGTTCCGCACCGTATGTTCTCCACCAAACGCATG
CTGCAGCGTTGGCGCCGCAAACTGAGCGCTCGTTTCCTGCGCAAAATCTATTAAgtcgacc
ggctgctaacaaagcccgaaaggaagctgagttggctgctgccaccgctgagcaataactagcataaccccttggggcctcta
aacgggtcttgaggggttttttgctgaaag

DNA Sequence of HsSIAT1 in pJL1 Context:
gaaattaatacgactcactatagggagaccacaacggtttccctctagaaataattttgtttaactttaagaaggagatatacatAT
GGAGAAAAAAATCTGGAGCCATCCGCAGTTCGAAAAAGGCTCCAAAGAGAAAAAAAAAG
GCAGCTACTACGATAGCTTCAAACTGCAGACCAAAGAATTTCAGGTTCTGAAAAGCCTG
GGTAAACTGGCAATGGGTAGCGATAGCCAGAGCGTTAGCAGCAGCAGTACCCAGGATC
CGCATCGTGGTCGTCAGACCCTGGGTAGCCTGCGTGGTCTGGCAAAAGCAAAACCGGA
AGCAAGCTTTCAGGTTTGGAATAAAGATTCCAGCAGCAAAAATCTGATTCCGCGTCTGCA
GAAAATCTGGAAAAACTATCTGAGCATGAACAAATACAAAGTGAGCTATAAAGGTCCGGG
TCCGGGTATCAAATTTTCAGCAGAAGCACTGCGTTGTCATCTGCGTGATCATGTTAATGT
TAGCATGGTTGAAGTTACCGATTTTCCGTTTAATACCAGCGAATGGGAAGGTTATCTGCC
GAAAGAAAGCATTCGTACCAAAGCAGGTCCGTGGGGTCGTTGTGCAGTTGTGAGCAGC
GCAGGTAGCCTGAAAAGCAGCCAGCTGGGTCGTGAAATTGATGATCATGATGCAGTTCT
GCGTTTTAATGGTGCACCGACCGCCAACTTTCAGCAGGATGTTGGCACCAAAACCACCA
TTCGTCTGATGAATAGTCAGCTGGTTACCACCGAAAAACGCTTTCTGAAAGATAGCCTGT
ATAACGAAGGTATTCTGATTGTTTGGGATCCGAGCGTTTATCATAGCGATATTCCGAAATG
GTATCAGAACCCGGATTACAACTTCTTCAACAACTATAAAACCTATCGCAAACTGCACCC
GAATCAGCCGTTTTATATCCTGAAACCGCAGATGCCGTGGGAACTGTGGGATATTCTGCA
AGAAATTAGTCCGGAAGAAATTCAGCCGAATCCGCCTAGCAGCGGTATGCTGGGTATTAT
CATTATGATGACCCTGTGTGATCAGGTGGATATCTATGAATTTCTGCCGAGCAAACGTAAA
ACCGATGTGTGTTATTACTATCAGAAATTCTTCGATAGCGCCTGTACCATGGGTGCATATC
ATCCGCTGCTGTATGAAAAAAATCTGGTGAAACACCTGAATCAGGGCACCGATGAAGATA
TTTATCTGCTGGGTAAAGCAACCCTGCCTGGTTTTCGTACCATTCATTGTTAAgtcgaccggct
gctaacaaagcccgaaaggaagctgagttggctgctgccaccgctgagcaataactagcataaccccttggggcctctaaacg
ggtcttgaggggttttttgctgaaag

DNA Sequence of PmST3,6 in pJL1 Context:
gaaattaatacgactcactatagggagaccacaacggtttccctctagaaataattttgtttaactttaagaaggagatatacatAT
GGAGAAAAAAATCTGGAGCCATCCGCAGTTCGAAAAAGGCTCCAAAAATCGTCGCCTGA
ACTTCAAACTGTTCTTCCTGATTATCTTTAGCCTGTTTAGCACCCTGAGCTGGTCAAAAAC
CATTACCCTGTATCTGGATCCGGCAAGCCTGCCTGCACTGAACCAGCTGATGGATTTTAC
CCAGAATAACGAGGATAAAACCCATCCGCGTATCTTTGGTCTGAGCCGCTTTAAAATCCC
GGATAACATTATTACCCAGTACCAGAACATCCACTTCGTGGAACTGAAAGATAACCGTCC
GACCGAAGCACTGTTTACCATTCTGGATCAGTATCCGGGTAATATCGAACTGAACATCCA
TCTGAATATTGCCCATAGCGTTCAGCTGATTCGTCCGATTCTGGCATATCGTTTTAAACAT

CTGGATCGTGTTAGCATTCAGCAGCTGAACCTGTATGATGATGGTAGCATGGAATATGTG
GATCTGGAAAAAGAAGAGAACAAAGATATCAGCGCAGAAATCAAACAGGCAGAAAAACA
GCTGAGCCATTATCTGCTGACCGGCAAAATCAAATTCGATAATCCGACCATTGCACGTTA
TGTTTGGCAGAGCGCATTTCCGGTTAAATATCATTTTCTGAGCACCGATTATTTTGAAAAA
GCCGAATTTCTGCAGCCGCTGAAAGAATATCTGGCAGAAAATTACCAGAAAATGGATTGG
ACCGCATATCAGCAACTGACACCGGAACAGCAGGCATTTTATCTGACCCTGGTTGGTTTT
AACGATGAAGTTAAACAGAGCCTGGAAGTTCAGCAGGCCAAATTTATCTTTACCGGCAC
CACCACCTGGGAAGGTAATACCGATGTTCGTGAATATTATGCACAGCAACAGCTGAATCT
GCTGAATCATTTTACACAGGCCGAAGGTGACCTGTTTATTGGTGATCATTACAAAATCTAT
TTCAAAGGTCATCCGCGTGGTGGCGAAATTAATGATTATATTCTGAACAACGCCAAAAAC
ATCACCAACATTCCGGCAAACATTAGCTTTGAAGTGCTGATGATGACCGGTCTGCTGCC
GGATAAAGTTGGTGGTGTTGCAAGCAGCCTGTATTTTAGCCTGCCGAAAGAAAAAATCA
GCCACATCATTTTCACCAGCAACAAACAGGTGAAAAGCAAAGAAGATGCACTGAATAAC
CCGTACGTTAAAGTTATGCGTCGTCTGGGTATTATTGATGAAAGCCAGGTGATCTTTTGG
GATAGCCTGAAACAGCTGTAAgtcgaccggctgctaacaaagcccgaaaggaagctgagttggctgctgccacc
gctgagcaataactagcataaccccttggggcctctaaacgggtcttgaggggttttttgctgaaag

DNA Sequence of CjCST-I in pJL1 Context:

gaaattaatacgactcactatagggagaccacaacggtttccctctagaaataattttgtttaactttaagaaggagatatacatAT
GGAGAAAAAAATCTGGAGCCATCCGCAGTTCGAAAAAGGCGGATCCGGAGGCAGCCAC
ATGACCCGTACCCGTATGGAAAATGAACTGATTGTGAGCAAAAACATGCAGAACATTATC
ATTGCAGGTAATGGTCCGAGCCTGAAAAACATTAACTATAAACGTCTGCCTCGCGAGTAT
GATGTTTTTCGTTGTAACCAGTTCTATTTCGAGGATAAATACTATCTGGGCAAAAAAATCA
AAGCCGTGTTTTTCAATCCGGGTGTTTTTCTGCAGCAGTATCATACCGCAAAACAGCTGA
TTCTGAAAAACGAGTACGAGATCAAAAACATCTTTTGCAGCACCTTTAACCTGCCGTTTAT
TGAAAGCAACGATTTCCTGCACCAGTTTTACAACTTTTTTCCGGATGCAAAACTGGGCTA
TGAAGTGATTGAAAACCTGAAAGAGTTCTACGCCTATATCAAATACAACGAGATCTATTTC
AACAAACGCATTACCAGCGGTGTTTATATGTGTGCAATTGCCATTGCCCTGGGCTATAAA
ACCATTTATCTGTGCGGTATCGATTTCTATGAAGGCGACGTTATTTATCCGTTTGAAGCAA
TGAGCACCAACATTAAAACAATCTTCCCTGGCATCAAAGACTTCAAACCGAGCAATTGTC
ACAGCAAAGAATATGATATTGAGGCCCTGAAACTGCTGAAAAGCATCTATAAAGTGAACAT
CTATGCCCTGTGTGATGATAGCATTCTGGCAAATCATTTTCCGCTGAGCATTAACATCAAC
AACAACTTTACCCTGGAAAACAAACACAACAACAGCATCAATGATATCCTGCTGACCGAT
AATACACCGGGTGTTAGCTTTTACAAAAATCAGCTGAAAGCCGATAACAAAATTATGCTGA
ACTTTTATTAAgtcgaccggctgctaacaaagcccgaaaggaagctgagttggctgctgccaccgctgagcaataacta
gcataaccccttggggcctctaaacgggtcttgaggggttttttgctgaaag

DNA Sequence of CjCST-II in pJL1 Context:

gaaattaatacgactcactatagggagaccacaacggtttccctctagaaataattttgtttaactttaagaaggagatatacatAT
GGAGAAAAAAATCTGGAGCCATCCGCAGTTCGAAAAAGGCGGATCCGGAAAAAAAGTG
ATCATTGCAGGTAATGGTCCGAGCCTGAAAGAAATTGATTATAGCCGTCTGCCGAACGAT
TTTGATGTTTTTCGTTGCAACCAGTTCTATTTCGAGGACAAATACTACCTGGGCAAAAAAT
GTAAAGCCGTGTTTTATAACCCGAGCCTGTTTTTCGAACAGTACTATACCCTGAAACATCT

GATCCAGAATCAAGAGTATGAAACCGAACTGATTATGTGCAGCAATTATAACCAGGCCCA
TCTGGAAAATGAGAACTTTGTGAAAACCTTCTATGACTATTTTCCGGATGCACATCTGGG
CTACGATTTTTTCAAACAGCTGAAAGATTTCAACGCCTACTTCAAATTTCACGAGATCTAT
TTTAACCAGCGCATTACCAGCGGTGTTTATATGTGTGCAGTTGCAATTGCCCTGGGCTAT
AAAGAAATTTATCTGAGCGGCATCGATTTCTATCAGAATGGTAGCAGCTATGCCTTTGACA
CCAAACAGAAAAATCTGCTGAAACTGGCACCGAACTTCAAAAATGATAACAGCCATTATAT
CGGCCATAGCAAAAACACCGATATTAAAGCACTGGAATTTCTGGAAAAAACCTATAAAATC
AAACTGTACTGCCTGTGTCCGAATAGCCTGCTGGCAAACTTTATTGAGCTGGCTCCGAAT
CTGAATAGCAACTTTATCATCCAAGAGAAAAACAACTATACCAAAGACATTCTGATTCCGA
GCAGCGAAGCCTATGGTAAATTTAGCAAAAATATCAACTAAgtcgaccggctgctaacaaagcccgaa
aggaagctgagttggctgctgccaccgctgagcaataactagcataaccccttggggcctctaaacgggtcttgaggggttttttg
ctgaaag

DNA Sequence of SpPvg1 in pJL1 Context:

gaaattaatacgactcactataggggagaccacaacggtttccctctagaaataattttgtttaactttaagaaggagatatacatAT
GGAGAAAAAAATCTGGAGCCATCCGCAGTTCGAAAAAGGCGGATCCGGAGACTTGCAA
ACTTTGAAGAACCCTAGTAGTCTAACTTCTCCTTCCTCGTCTACTTCGGTTGACAAGAAA
AAGCCCCTTTTCACCAAATCACCCAGAAATAGTGCTTCCTGTGAATCCACCATCACTCTG
CAATCCAACTTACTCTTCACTTATTACAAGCATTACTTTGCAGGCATCAAGAAGGTTGCGC
TCATTGGGTTTCCTGACCACCCCAACAAGGGTGATAGTGCAATCTATGTTGCTGAGAAAA
AGCTTTTGGATGCTTTGAATATTGAGGTTGTCTACATTACTGCTCAAGAGGCTGACTACTC
TGCTTCTGAGTTGAAGTCGATCATCTCTGATATCCCCAGAGATGAGTTCGCACTTGCTTT
CCACGGCGGTGGTAACTTTGGCGATTTATATCCTGACCATCAGCATTTACGTGAACTTGT
CGTGCGAGATTTCCCTTCCTTCACCACCATATCCTTCCCTCAATCTGTCTGGTATAATGAA
CAACAACTTCTCGAACAGGCCTCTATTTTGTATGCCGAAAATCCTAATATCACTTTGGTCA
CTCGTGATAGGCAAAGCTATGGTTTTGCCGTTGATGCTTTTGGCAAGCATAATGAAGTTC
TTCTTACCCCCGATATCGTCTTCTTCATGGGCCCCATCCCTGAGATTCGCGAGGCTACTC
CCATCACTCATGATGTGTTGATTCTTGCTCGTCTCGATCACGAGGGTGGTCAGCAACAT
GGTGCTGAAGACTATTATCGCGATACTTTGAATGCCGCTAACTTGACCTACAGCGTTGAG
GATTGGCTCTTGTGGGATCCTCCTGTTGCTCAAAATCCCGATTCTTCCTTTGATGATAGA
GGCCAAGCTCGTTACGAAGCTGGTGCGGAATTCCTTGCCTCTGCTCGCGTCGTCATTAC
TGATCGTCTCCATGCTCACATCCTTAGCACTTTAATGGGTATTCCTCATATCGTCGTTGAA
AACTCCCAAATGGGAAAAATTACTAACTATCATAATACCTGGCTACATGGTTGCACATTGG
ATGGTGTCAGTGTAGTCGTTGATTCCGTTGACAAGGCTTTGTCTTTGCTTCTCGAGTGG
AATGAGGCCGGCTACTTTTAAgtcgaccggctgctaacaaagcccgaaaggaagctgagttggctgctgccacc
gctgagcaataactagcataaccccttggggcctctaaacgggtcttgaggggttttttgctgaaag

DNA Sequence of VsST3 in pJL1 Context:

gaaattaatacgactcactataggggagaccacaacggtttccctctagaaataattttgtttaactttaagaaggagatatacatAT
GGAGAAAAAAATCTGGAGCCATCCGCAGTTCGAAAAAGGCGGATCCGGAGGAAATGATA
ATAGCACCACCACCAATAATAACGCCATCGAAATTTATGTTGATCGTGCAACCCTGCCGA
CCATTCAGCAGATGACCAAAATTGTTAGCCAGAAAACCAGCAACAAAAAACTGATTAGCT
GGTCACGTTATCCGATCACCGATAAAAGCCTGCTGAAAAAAATCAACGCCGAGTTTTTCA

AAGAACAGTTTGAACTGACCGAGAGCCTGAAAAACATTATTCTGAGCGAAAACATCGATA
ACCTGATTATTCATGGTAACACCCTGTGGTCAATTGATGTGGTGGATATTATCAAAGAAGT
GAACCTGCTGGGTAAAAACATTCCGATTGAACTGCACTTTTATGATGATGGCAGCGCAGA
ATATGTGCGCATTTATGAATTTAGCAAACTGCCGGAAAGCGAACAGAAATACAAAACCAG
CCTGAGCAAAAACAACATCAAATTTAGCATTGATGGCACCGATAGCTTTAAAAACACCATC
GAAAACATTTACGGCTTCAGCCAGCTGTATCCGACCACCTACCACATGCTGCGTGCAGA
TATTTTTGATACCACCCTGAAAATTAACCCGCTGCGTGAACTGCTGAGCAACAACATTAAA
CAAATGAAATGGGACTACTTCAAAGACTTCAACTATAAACAGAAAGACATCTTTTATAGCC
TGACCAACTTTAACCCGAAAGAGATCCAAGAGGACTTTAACAAAAACAGCAATAAAAACT
TCATCTTCATCGGCAGCAATAGCGCAACCGCCACCGCAGAAGAACAAATTAACATTATTA
GCGAAGCCAAAAAAGAAAACAGCAGCATTATTACCAACAGCATCAGCGATTATGACCTGT
TCTTTAAAGGTCATCCGAGCGCAACCTTTAATGAGCAGATTATTAACGCCCACGATATGAT
CGAGATCAACAACAAAATTCCGTTTGAAGCCCTGATCATGACCGGTATTCTGCCGGATG
CAGTTGGTGGTATGGGTAGCAGCGTGTTTTTTAGCATTCCGAAAGAGGTGAAAAACAAAT
TCGTGTTCTATAAAAGCGGCACCGACATTGAAAACAATAGCCTGATTCAGGTTATGCTGA
AACTGAATCTGATTAACCGCGATAACATCAAACTGATCAGCGATATTTAAgtcgaccggctgctaa
caaagcccgaaaggaagctgagttggctgctgccaccgctgagcaataactagcataaccccttggggcctctaaacgggtctt
gaggggttttttgctgaaag

DNA Sequence of HpFutA in pJL1 Context:
gaaattaatacgactcactataggggagaccacaacggtttccctctagaaataattttgtttaactttaagaaggagatatacatAT
GTTCCAACCATTATTAGACGCGTTCATCGAGTCGGCCTCTATCGAGAAGATGGCGTCGA
AGTCACCACCCCCGCCCCTCAAGATCGCCGTGGCCAACTGGTGGGGAGACGAGGAGA
TTAAGGAATTTAAGAAGTTTGTTTTGTACTTCATTTTGAGTCAACGCTACGCGATCACTTT
GCACCAAAACCCTAACGAGTTCTCGGACTTAGTCTTCTCAAACCCTCTTGGCGCGGCCC
GCAAGATCTTGAGTTACCAAAACACGAAGCGTGTTTTCTACACAGGCGAGAACGAGTCC
CCAAATTTTAATTTGTTCGACTACGCGATCGGCTTTGACGAACTTGACTTCAACGACCGC
TACTTGCGCATGCCTTTATACTACAATGAGTTACACATCAAGGCAGAACTCGTGAACGAC
ACTACTGCGCCTTACAAGTTGAAGGACAACAGTTTATATGCGCTCAAGAAGCCTAGTCAC
CACTTCAAGGAGAATCACCCTAACCTCTGCGCTGTCGTCAACGACGAGTCGGACCTGC
TCAAGCGCGGCTTCGCGTCGTTCGTGGCGTCTAACGCGAACGCCCCAATGCGCAACGC
TTTCTACGACGCACTCAACAGTATCGAGCCTGTGACAGGCGGCGGCAGTGTGCGCAAC
ACTTTAGGCTACAAGGTAGGTAATAAGTCGGAGTTCCTCTCGCAATACAAGTTTAACTTGT
GTTTTGAGAATAGTCAGGGCTACGGCTACGTTACGGAGAAGATCCTTGACGCGTACTTTT
CACACACTATCCCTATCTACTGGGGCTCCCCTTCTGTCGCCAAGGACTTTAACCCAAAGT
CGTTCGTCAATGTTCATGACTTTAATAATTTCGACGAGGCAATTGATTATATTAAGTATCTC
CACACTCACCCCAACGCTTACCTTGACATGTTGTACGAGAACCCTCTCAACACCCTCGA
CGGAAAGGCGTACTTTTACCAAGACCTGAGTTTTAAGAAGATTTTGGATTTCTTCAAAAC
TATTCTGGAAAACGACACTATTTACCATAAGTTCTCCACGAGTTTTATGTGGAGTACGAC
TTGCACAAGCCACTCGTCTCAATCGACGACTTGCGCGTTAACTACGGTAGTTCGGCATG
GTCACACCCCCAATTTGAGAAGGGAGGAGGGAGTGGAGGAGGCAGCGGCGGCAGTG
CCTGGTCGCACCCCCAATTTGAGAAGTGAgtcgaccggctgctaacaaagcccgaaaggaagctgagttg
gctgctgccaccgctgagcaataactagcataaccccttggggcctctaaacgggtcttgaggggttttttgctgaaag

DNA Sequence of HpFutC in pJL1 Context:
gaaattaatacgactcactatagggagaccacaacggtttccctctagaaataattttgtttaactttaagaaggagatatacatAT
GGAGAAAAAAATCTGGAGCCATCCGCAGTTCGAAAAAGGCGGATCCGGAGCCTTTAAA
GTTGTTCAGATTTGTGGTGGTCTGGGCAATCAGATGTTTCAGTATGCATTTGCAAAAAGC
CTGCAGAAACATAGCAATACACCGGTTCTGCTGGATATTACCAGCTTTGATTGGAGCGAT
CGTAAAATGCAGCTGGAACTGTTTCCGATTGATCTGCCGTATGCAAGCGCAAAAGAAATT
GCCATTGCAAAGATGCAGCATCTGCCGAAACTGGTTCGTGATGCACTGAAATGTATGGG
TTTTGATCGTGTGAGCCAAGAAATCGTGTTTGAATATGAACCGAAACTGCTGAAACCGAG
CCGTCTGACCTATTTTTTCGGTTATTTTCAAGATCCGCGTTACTTCGATGCAATTAGTCCG
CTGATTAAACAGACCTTTACACTGCCTCCGCCTCCGGAAAATAACAAAAACAACAATAAG
AAAGAAGAGGAATATCAGTGCAAGCTGAGCCTGATTCTGGCAGCAAAAAATAGCGTTTTT
GTGCATATTCGTCGCGGTGATTATGTTGGTATTGGTTGTCAGCTGGGTATCGACTATCAG
AAAAAAGCACTGGAATATATGGCAAAACGTGTGCCGAATATGGAACTTTTTGTTTTTTGTG
AGGACCTGGAATTTACCCAGAATCTGGATCTGGGCTATCCGTTTATGGATATGACCACAC
GTGATAAAGAAGAAGAGGCCTATTGGGATATGCTGCTGATGCAGAGCTGTCAGCATGGT
ATTATTGCAAATAGCACCTATAGTTGGTGGGCAGCCTATCTGATTGAAAATCCGGAAAAAA
TCATCATCGGTCCGAAACATTGGCTGTTTGGCCATGAAAACATTCTGTGTAAAGAATGGG
TGAAAATCGAAAGCCACTTTGAAGTGAAAAGCCAGAAATATAACGCCTAAgtcgaccggctgct
aacaaagcccgaaaggaagctgagttggctgctgccaccgctgagcaataactagcataaccccttggggcctctaaacgggt
cttgaggggttttttgctgaaag

DNA Sequence of BtGGTA in pJL1 Context:
gaaattaatacgactcactatagggagaccacaacggtttccctctagaaataattttgtttaactttaagaaggagatatacat
ATGGAGAAAAAAATCTCTGCGTGGAGCCATCCGCAGTTCGAAAAAGGATCCGAGTCTAA
ACTGAAACTGTCTGACTGGTTTAACCCGTTTAAACGCCCGGAAGTAGTGACTATGACCAA
ATGGAAAGCTCCGGTGGTTTGGGAAGGCACCTACAACCGCGCAGTTCTGGACAATTACT
ATGCAAAACAAAAAATCACTGTTGGTCTGACCGTATTTGCCGTTGGCCGTTACATTGAGC
ATTACCTGGAAGAATTCCTGACCAGCGCAAACAAACACTTCATGGTGGGCCACCCTGTT
ATCTTCTATATTATGGTAGATGATGTTAGCCGTATGCCGCTGATTGAACTGGGCCCGCTGC
GTTCCTTCAAAGTCTTCAAGATCAAACCGGAAAAACGCTGGCAGGACATCTCCATGATG
CGCATGAAAACCATCGGTGAACACATCGTGGCACATATTCAACACGAAGTCGATTTTCTG
TTCTGCATGGATGTTGATCAGGTTTTCCAGGATAAATTCGGCGTTGAAACCCTGGGTGA
GAGCGTGGCACAGCTGCAGGCGTGGTGGTACAAGGCGGACCCGAACGATTTCACCTAT
GAACGTCGTAAAGAAAGCGCCGCTTACATTCCGTTTGGTGAAGGCGATTTCTATTATCAC
GCGGCGATTTTTGGCGGTACCCCGACCCAAGTTCTGAACATCACCCAGGAATGCTTCAA
AGGCATTCTGAAAGACAAAAAAAACGATATCGAAGCACAGTGGCATGACGAATCTCACC
TGAACAAATATTTCCTGCTGAACAAACCGACCAAAATTCTGTCTCCGGAATATTGTTGGG
ACTATCACATCGGTCTGCCGGCCGACATCAAACTGGTGAAAATGTCTTGGCAGACGAAA
GAATATAACGTAGTACGTAACAATGTCTAAgtcgaccggctgctaacaaagcccgaaaggaagctgagttgg
ctgctgccaccgctgagcaataactagcataaccccttggggcctctaaacgggtcttgaggggttttttgctgaaag

DNA Sequence of HdGlcNAcT in pJL1 Context:

gaaattaatacgactcactataggggagaccacaacggtttccctctagaaatctagataaataaggaggtaataaATGACTA
CCCTGGTGTCTGTGCTGATTTGCGCTTACAACGTCGAAAAATATATCGATGAGTGTCTGA
ACGCCGTCATTGCACAGACTTACAAAAACCTGGAAATCATTGTTGTAAACGACGGCTCCA
CGGATGGCACTCTGGCTAAACTGCGCCAGTTCGAGGCGAAAGATCCACGCGTAAAAAT
CATTGACAACATTGTAAACCAGGGTACTTCTAAGTCTCTGAATATCGGTATCCAGTACTGT
CAGGGCGAAATTATCGCACGTACCGACTCCGATGATATCGTGGACATCCATTGGATTGAA
ACGCTGATGCGTGAGCTGGACAATTCCCCGGAAACTATCGCTATCTCTGCGTACCTGGA
ATTCCTGGCGGAGAAAGGTAACGGTAGCAAACTGTCCCGCTCTCGTAAACATGGCAAGA
ATGCAGAGAACCCGATCAGCAGCGAGGCGATCTCCCAGCGTATGCTGTTCGGTAATCC
GGTTCACAACAACGTCGCACTGGTGCGTCGTAAAGTATTCTCCGAGTACGGTCTGCGTT
TCGACCCGGACTATATCCACGCTGAAGACTATAAATTCTGGTTCGAAGTAAGCAAACTGG
GCAAGATGCGTACTTACCCAAAAGCGCTGGTTAAATACCGTCTGCACGCTACCCAGGTT
AGCAGCGCATATAACCAGAAACAGCGTTCTATTGCAAAAAAAATCAAACGTGAGGCCATC
TCTCATTACCTGCAGCAGTACGGCATTCAGCTGCCGGAAAAACTGACTATCCACGACCT
GTTCTCTATCTTCTCCCCGCAGATTGAACTGAGCCTGACCGTTGCGAACAAACAGGAAC
TGTTCTGGTCTCTGGCAACTTCTCTGTCTGAATATCACTTCCGTGATCTGCTGAAAATCTA
TTCCCTGGATATCTTCCACCAACTGTCCTTCAAATACAAAAAGCGCATTTTTCGTAAGTTC
CTGCTGCCGAACCGCTACCCATCTGTAATCTAAgtcgaccggctgctaacaaagcccgaaaggaagctg
agttggctgctgccaccgctgagcaataactagcataaccccttggggcctctaaacgggtcttgaggggttttttgctgaaag

DNA Sequence of NgLgtA in pJL1 Context:
gaaattaatacgactcactataggggagaccacaacggtttccctctagaaataattttgtttaactttaagaaggagatatacatAT
GCCGAGCGAAGCATTTCGTCGTCATCGTGCATATCGTGAAAACAAACTGCAGCCGCTGG
TTAGCGTTCTGATTTGTGCATATAATGTGGAAAAATACTTTGCCCAGAGCCTGGCAGCAG
TTGTTAATCAGACCTGGCGTAATCTGGATATTCTGATTGTTGATGATGGTAGCACCGATGG
CACCCTGGCAATTGCACAGCGTTTTCAAGAACAGGATGGTCGTATTCGTATTCTGGCAC
AGCCTCGTAATAGCGGTCTGATTCCGAGCCTGAATATTGGTCTGGATGAACTGGCAAAAA
GCGGTGGTGGTGGCGAATATATTGCACGTACCGATGCAGATGATATTGCAGCACCGGAT
TGGATTGAAAAAATTGTGGGTGAGATGGAAAAAGATCGCAGCATTATTGCAATGGGTGCA
TGGCTGGAAGTTCTGAGCGAAGAAAAAGATGGTAATCGTCTGGCACGTCATCATGAACA
TGGTAAAATTTGGAAAAAACCGACGCGTCATGAAGATATCGCAGATTTTTTTCCGTTTGG
CAACCCGATTCATAACAACACCATGATTATGCGTCGTAGCGTTATTGATGGTGGTCTGCG
TTATAATACCGAACGTGATTGGGCAGAAGATTATCAGTTTTGGTATGATGTTAGCAAACTG
GGTCGTCTGGCCTATTATCCGGAAGCACTGGTTAAATATCGCCTGCATGCAAATCAGGTT
AGCAGCAAATATAGCATTCGCCAGCATGAAATTGCCCAGGGTATTCAGAAAACCGCACGT
AATGATTTTCTGCAGAGCATGGGCTTTAAAACCCGTTTTGATAGCCTGGAATATCGCCAG
ATTAAAGCAGTTGCCTATGAACTGCTGGAAAAGCATCTGCCGGAAGAAGATTTTGAACG
CGCACGTCGTTTTCTGTATCAGTGTTTTAAACGCACCGATACACTGCCTGCCGGTGCAT
GGTTAGATTTTGCAGCAGATGGTCGCATGCGTCGTCTGTTTACCCTGCGTCAGTATTTTG
GTATTCTGCATCGTCTGCTGAAAAACCGTTAAgtcgaccggctgctaacaaagcccgaaaggaagctgag
ttggctgctgccaccgctgagcaataactagcataaccccttggggcctctaaacgggtcttgaggggttttttgctgaaag

DNA Sequence of PdST6 in pJL1 Context:

gaaattaatacgactcactatagggagaccacaacggtttccctctagaaataattttgtttaactttaagaaggagatatacatAT
GGAGAAAAAAATCTGGAGCCATCCGCAGTTCGAAAAAGGCGGATCCGGACTGGTTCCG
CGTGGTAGCCACATGTGTAATAGCGATAACACCAGCCTGAAAGAAACCGTTAGCAGCAA
TAGCGCAGATGTTGTTGAAACCGAAACCTATCAGCTGACCCCGATTGATGCACCGAGCA
GCTTTCTGAGCCATAGCTGGGAACAGACCTGTGGCACCCCGATTCTGAATGAAAGCGAT
AAACAGGCAATCAGCTTTGATTTTGTTGCACCGGAACTGAAACAGGATGAGAAATATTGC
TTTACCTTCAAAGGCATTACCGGTGATCATCGTTATATTACCAATACCACCCTGACCGTTG
TGGCACCGACCCTGGAAGTTTATATTGATCATGCAAGCCTGCCGAGCCTGCAGCAGCTG
ATTCATATTATTCAGGCCAAAGATGAATATCCGAGCAATCAGCGTTTTGTTAGCTGGAAAC
GTGTTACCGTTGATGCAGATAATGCCAACAAACTGAACATTCATACCTATCCGCTGAAAG
GCAATAATACCAGTCCGGAAATGGTTGCAGCAATTGATGAATATGCACAGAGCAAAAATC
GCCTGAACATCGAGTTTTATACCAATACAGCCCACGTGTTTAATAACCTGCCTCCGATTAT
TCAGCCGCTGTATAATAACGAGAAAGTGAAAATTAGCCACATCAGCCTGTATGATGACGG
TAGCAGCGAATATGTTAGCCTGTATCAGTGGAAAGATACCCCGAACAAAATTGAAACACT
GGAAGGTGAAGTTAGCCTGCTGGCAAATTATCTGGCAGGCACCTCACCGGATGCTCCG
AAAGGTATGGGTAATCGTTATAATTGGCACAAACTGTATGACACCGACTATTACTTTCTGC
GCGAAGATTATCTGGATGTTGAAGCAAATCTGCATGATCTGCGTGATTACCTGGGTAGCA
GTGCAAAACAAATGCCGTGGGATGAATTTGCAAAACTGAGCGATAGCCAGCAGACCCTG
TTTCTGGATATTGTTGGTTTTGATAAAGAACAGCTGCAGCAACAGTATAGCCAGAGTCCG
CTGCCGAATTTTATCTTTACCGGCACCACCACCTGGGCAGGCGGTGAAACCAAAGAATA
TTATGCCCAGCAGCAGGTTAACGTGATTAACAATGCAATTAATGAAACCAGCCCGTACTAT
CTGGGTAAAGATTATGACCTGTTTTTCAAAGGTCATCCTGCCGGTGGTGTGATTAATGAT
ATTATTCTGGGTAGCTTCCCGGATATGATTAACATTCCGGCAAAAATTAGCTTCGAGGTTC
TGATGATGACCGATATGCTGCCGGATACCGTTGCAGGTATTGCAAGCAGTCTGTATTTCA
CAATTCCGGCAGATAAAGTGAACTTCATTGTTTTTACCAGCAGCGATACCATTACCGATC
GTGAAGAAGCACTGAAAAGTCCGCTGGTTCAGGTTATGCTGACCCTGGGTATTGTTAAA
GAAAAAGATGTTCTGTTTTGGGCATAAgtcgaccggctgctaacaaagcccgaaaggaagctgagttggctg
ctgccaccgctgagcaataactagcataaccccttggggcctctaaacgggtcttgaggggttttttgctgaaag

DNA Sequence of PIST6 in pJL1 Context:

gaaattaatacgactcactatagggagaccacaacggtttccctctagaaataattttgtttaactttaagaaggagatatacatAT
GGAGAAAAAAATCTGGAGCCATCCGCAGTTCGAAAAAGGCGGATCCGGAGGATGTAAT
GATAATCAGAATACCGTTGATGTTGTTGTGAGCACCGTGAATGATAACGTGATTGAAAATA
ACACCTACCAGGTGAAACCGATTGATACCCCGACCACCTTTGATAGCTATAGTTGGATTC
AGACCTGTGGCACCCCGATTCTGAAAGATGATGAGAAATATAGCCTGAGCTTTGATTTTG
TTGCACCGGAACTGGATCAGGATGAAAAATTCTGTTTTGAGTTTACCGGTGATGTGGATG
GTAAACGTTATGTTACCCAGACCAATCTGACCGTTGTGGCACCGACCCTGGAAGTTTATG
TTGATCATGCAAGCCTGCCGAGCCTGCAGCAGCTGATGAAAATTATCCAGCAGAAAAAC
GAGTATAGCCAGAACGAACGTTTTATTAGCTGGGGTCGTATTGGTCTGACCGAAGATAAT
GCCGAAAAACTGAATGCACATATTTATCCGCTGGCAGGTAATAATACCAGCCAAGAACTG
GTTGATGCCGTTATTGATTATGCCGATAGCAAAAATCGTCTGAACCTGGAACTGAATACCA
ATACCGCACATAGCTTTCCGAATCTGGCACCGATTCTGCGTATTATTAGCAGCAAAAGCA
ATATCCTGATCAGCAACATTAACCTGTATGATGATGGTAGCGCAGAATATGTGAATCTGTAT

AACTGGAAAGACACCGAGGATAAAAGCGTTAAACTGAGCGATAGCTTTCTGGTGCTGAA
AGATTATTTCAATGGCATCAGCAGCGAAAAACCGAGCGGTATTTATGGTCGTTATAATTGG
CACCAGCTGTATAACACCAGCTATTACTTTCTGCGCAAAGATTATCTGACAGTTGAACCG
CAGCTGCATGATCTGCGTGAATATCTGGGTGGTAGCCTGAAACAAATGAGCTGGGATGG
TTTTAGCCAGCTGAGCAAAGGTGATAAAGAACTGTTTCTGAACATCGTGGGCTTCGATCA
AGAAAAACTGCAGCAAGAATATCAGCAGAGCGAACTGCCGAATTTTGTTTTTACCGGCA
CCACCACCTGGGCAGGCGGTGAAACCAAAGAATATTATGCACAGCAGCAGGTTAACGTG
GTGAATAATGCAATTAATGAAACCAGCCCGTATTATCTGGGTCGTGAACATGACCTGTTTT
TCAAAGGTCATCCGCGTGGTGGTATTATCAACGATATTATTCTGGGCAGCTTCAACAACAT
GATTGACATTCCGGCAAAAGTCAGCTTTGAAGTTCTGATGATGACCGGTATGCTGCCGG
ATACCGTGGGTGGTATTGCAAGCAGCCTGTATTTTTCAATTCCGGCAGAAAAAGTGAGCT
TCATTGTGTTTACCAGCAGCGATACCATTACCGATCGTGAAGATGCACTGAAAAGTCCGC
TGGTTCAGGTTATGATGACCCTGGGTATTGTGAAAGAAAAAGATGTGCTGTTTTGGAGC
GATCTGCCGGATTGTAGCAGCGGTGTTTGTATTGCACAGTATTAAgtcgaccggctgctaacaaag
cccgaaaggaagctgagttggctgctgccaccgctgagcaataactagcataaccccttggggcctctaaacgggtcttgaggg
gttttttgctgaaag

DNA Sequence of PpST3 in pJL1 Context:
gaaattaatacgactcactatagggagaccacaacggtttccctctagaaataattttgtttaactttaagaaggagatatacatAT
GGGAAAAAACAAAACCATCGAAGTTTATGTTGATCGTGCAACCCTGCCGACCATTCAGC
AGATGACCCAGATTATTAACGAAAACAGCAACAACAAAAAACTGATCAGCTGGTCACGCT
ATCCGATTAATGATGAAACCCTGCTGGAAAGCATTAACGGCAGCTTTTTCAAAAATCGTC
CGGAACTGATTAAAAGCCTGGATAGCATGATTCTGACCAACGAGATCAAAAAAGTGATTA
TCAATGGCAATACCCTGTGGGCAGTTGATGTTGTGAATATCATTAAAAGCATTGAGGCCC
TGGGCAAAAAAACCGAAATTGAACTGAACTTCTATGATGATGGCAGCGCAGAATATGTTC
GCCTGTATGATTTTAGCCGTCTGCCGGAAAGCGAACAAGAATACAAAATTAGCCTGAGCA
AAGACAACATTCAGAGCAGCATTAATGGCACCCAGCCGTTTGATAATAGCATCGAAAACA
TTTATGGCTTTAGCCAGCTGTATCCGACCACCTACCACATGCTGCGTGCAGATATCTTTG
AAACCAATCTGCCGCTGACCAGCCTGAAACGTGTTATTAGCAATAATATCAAACAAATGAA
ATGGGATTACTTCACCACCTTCAATAGCCAGCAGAAAAACAAATTCTATAACTTTACCGGC
TTTAACCCGGAAAAAATCAAAGAGCAGTATAAAGCAAGTCCGCACGAAAACTTTATCTTTA
TTGGCACCAATAGCGGCACCGCAACCGCAGAACAGCAGATTGATATTCTGACCGAAGCC
AAAAAACCGGATAGCCCGATTATTACCAATAGCATTCAGGGTCTGGACCTGTTTTTCAAA
GGTCATCCGAGCGCAACCTATAACCAGCAGATTATTGATGCCCATAACATGATCGAGATC
TATAACAAAATTCCGTTCGAAGCCCTGATTATGACCGATGCACTGCCGGATGCAGTTGGT
GGTATGGGTAGCAGCGTGTTTTTTAGCCTGCCGAATACCGTGGAAAACAAATTTATCTTC
TACAAAAGCGACACCGACATTGAAAACAATGCACTGATTCAGGTGATGATCGAACTGAAT
ATTGTGAATCGCAACGACGTGAAACTGATTAGCGATCTGCAGTAAgtcgaccggctgctaacaaa
gcccgaaaggaagctgagttggctgctgccaccgctgagcaataactagcataaccccttggggcctctaaacgggtcttgagg
ggttttttgctgaaag

DNA Sequence of H1HA10 in pJL1 Context:
gaaattaatacgactcactatagggagaccacaacggtttccctctagaaataattttgtttaactttaagaaggagatatacat

ATGGAGAAAAAAATCCATCACCATCATCACCATGGTAGCAAAGCGACTACC**GGAGGTAA**
CTGGACAACAGCTGGCGGCAAAGGATCCGATACCGTTGATACCGTGCTGGAAAAAAAT
GTTACCGTTACACATAGCGTGAACCTGCTGGAAGATAGCCATCGTAGCGCAAATAGCAG
CCTGCCGTATCAGAATACCCATCCGACCACCAATGGTGAAAGCCCGAAATATGTTCGTAG
CGCCAAACTGCGTATGGTTACCGGTCTGCGTAATGGTAGCGCAGGTAGCGCGACCCAG
AATGCAATTAATGGTATTACCAATAAGGTGAACACCGTGATCGAGAAAATGAACATTCAGG
ATACCGCAACCGGCAAAGAATTTAACAAAGATGAAAAGCGCATGGAAAACCTGAACAAA
AAAGTGGATGATGGCTTTCTGGATATCTGGACCTATAATGCAGAACTGCTGGTGTTACTG
GAAAACGAACGTACCCTGGATGCACATGATAGCCAAGGCACCGGTGGTGGTTATATTCC
GGAAGCACCGCGTGATGGTCAGGCCTATGTTCGTAAAGATGGTGAATGGGTTCTGCTGA
GCACCTTTCTGTAAgtcgaccggctgctaacaaagcccgaaaggaagctgagttggctgctgccaccgctgagcaat
aactagcataacccttggggcctctaaacgggtcttgaggggttttttgctgaaag

DNA Sequence of ApNGT in pMAF10 Context:

ttgctatgccatagcatttttatccataagattagcggatcctacctgacgctttttatcgcaactctctactgtttctccatacccgttttttt
gggctagcaggaggaattccATGGAAAACGAGAATAAACCGAACGTGGCAAATTTTGAAGCAGCA
GTTGCAGCCAAAGATTATGAAAAAGCATGTAGCGAGCTGCTGCTGATTCTGAGCCAGCT
GGATAGCAATTTTGGTGGCATTCATGAAATCGAGTTCGAGTACCCAGCTCAGCTGCAGG
ATCTGGAGCAGGAAAAAATTGTGTACTTTTGCACCCGTATGGCGACTGCCATCACCACC
CTGTTCTCTGACCCGGTTCTGGAAATCTCCGACCTGGGTGTGCAGCGTTTCCTGGTTTA
TCAGCGTTGGCTGGCGCTGATCTTCGCTAGCAGCCCGTTCGTGAACGCAGACCACATC
CTGCAGACTTACAACCGTGAACCGAACCGCAAAAACTCCCTGGAAATTCACCTGGACTC
TAGCAAGTCCTCTCTGATTAAATTTTGCATCCTGTACCTGCCGGAATCTAATGTGAACCTG
AATCTGGATGTGATGTGGAACATTTCCCCGGAGCTGTGTGCCTCTCTGTGCTTTGCGCT
GCAAAGCCCGCGTTTTGTTGGCACCAGCACCGCCTTTAACAAACGCGCGACCATTCTG
CAGTGGTTCCCGCGTCATCTGGACCAGCTGAAAAACCTGAACAACATCCCGTCCGCTAT
CTCTCATGACGTGTATATGCACTGTTCTTACGACACCAGCGTTAACAAGCACGATGTTAA
GCGCGCGCTGAATCACGTGATTCGTCGCCACATCGAATCCGAATACGGTTGGAAAGATC
GTGATGTGGCTCACATCGGTTATCGCAACAACAAACCGGTTATGGTCGTTCTGCTGGAA
CATTTTCATAGCGCGCACTCTATCTACCGTACTCACTCTACCAGCATGATCGCCGCGCGC
GAACACTTCTATCTGATCGGCCTGGGTTCCCCGAGCGTTGACCAGGCCGGTCAGGAGG
TTTTCGATGAATTCCACCTGGTAGCGGGTGACAACATGAAGCAAAAACTGGAATTCATTC
GTTCTGTGTGCGAAAGCAATGGTGCGGCAATTTTCTACATGCCGAGCATCGGTATGGATA
TGACCACCATCTTCGCGTCCAATACCCGTCTGGCGCCGATTCAGGCAATCGCCCTGGG
CCACCCGGCGACTACTCACTCCGACTTCATTGAATACGTTATCGTGGAAGACGACTACG
TCGGCTCTGAGGAATGCTTCTCTGAAACCCTGCTGCGTCTGCCGAAAGACGCTCTGCC
GTATGTTCCGTCCGCCCTGGCTCCGGAGAAAGTTGATTACCTGCTGCGTGAAAACCCTG
AAGTTGTCAACATCGGTATTGCCTCTACCACTATGAAGCTGAACCCGTACTTCCTGGAAG
CACTGAAGGCCATTCGTGACCGTGCGAAGGTGAAAGTGCACTTCCACTTCGCACTGGG
CCAGTCCAATGGTATCACTCACCCTTACGTTGAACGCTTTATCAAATCTTACCTGGGCGA
CAGCGCTACCGCGCACCCGCACTCTCCGTACCACCAGTACCTGCGTATTCTGCACAACT
GCGATATGATGGTAAACCCTTTTCCGTTTGGTAATACCAATGGTATTATTGACATGGTAAC
CCTGGGTCTGGTAGGTGTTTGCAAAACCGGTGCGGAAGTCCACGAACATATCGATGAAG

GCCTGTTCAAACGTCTGGGCCTGCCGGAATGGCTGATTGCAAACACCGTGGACGAATA
CGTGGAACGTGCAGTGCGCCTGGCCGAGAACCATCAGGAACGTCTGGAACTGCGTCG
TTACATTATTGAAAACAATGGCCTGAACACCCTGTTCACCGGCGACCCACGCCCGATGG
GTCAGGTGTTCCTGGAAAAACTGAACGCATTCCTGAAGGAAAACGGCGGCGACTACAA
GGACGATGACGACAAGGGATAAaagcttggctgttttggcggatgagagaagattttcagcctgatacagattaaat
cagaac

DNA Sequence of NmLgtB.ApNGT in pMAF10 Context:

ttgctatgccatagcatttttatccataagattagcggatcctacctgacgctttttatcgcaactctctactgtttctccatacccgttttttt
gggctagcaggaggaattccATGGAAAACGAGAATAAACCGAACGTGGCAAATTTTGAAGCAGCA
GTTGCAGCCAAAGATTATGAAAAAGCATGTAGCGAGCTGCTGCTGATTCTGAGCCAGCT
GGATAGCAATTTTGGTGGCATTCATGAAATCGAGTTCGAGTACCCAGCTCAGCTGCAGG
ATCTGGAGCAGGAAAAAATTGTGTACTTTTGCACCCGTATGGCGACTGCCATCACCACC
CTGTTCTCTGACCCGGTTCTGGAAATCTCCGACCTGGGTGTGCAGCGTTTCCTGGTTTA
TCAGCGTTGGCTGGCGCTGATCTTCGCTAGCAGCCCGTTCGTGAACGCAGACCACATC
CTGCAGACTTACAACCGTGAACCGAACCGCAAAAACTCCCTGGAAATTCACCTGGACTC
TAGCAAGTCCTCTCTGATTAAATTTTGCATCCTGTACCTGCCGGAATCTAATGTGAACCTG
AATCTGGATGTGATGTGGAACATTTCCCCGGAGCTGTGTGCCTCTCTGTGCTTTGCGCT
GCAAAGCCCGCGTTTTGTTGGCACCAGCACCGCCTTTAACAAACGCGCGACCATTCTG
CAGTGGTTCCCGCGTCATCTGGACCAGCTGAAAAACCTGAACAACATCCCGTCCGCTAT
CTCTCATGACGTGTATATGCACTGTTCTTACGACACCAGCGTTAACAAGCACGATGTTAA
GCGCGCGCTGAATCACGTGATTCGTCGCCACATCGAATCCGAATACGGTTGGAAAGATC
GTGATGTGGCTCACATCGGTTATCGCAACAACAAACCGGTTATGGTCGTTCTGCTGGAA
CATTTTCATAGCGCGCACTCTATCTACCGTACTCACTCTACCAGCATGATCGCCGCGCGC
GAACACTTCTATCTGATCGGCCTGGGTTCCCCGAGCGTTGACCAGGCCGGTCAGGAGG
TTTTCGATGAATTCCACCTGGTAGCGGGTGACAACATGAAGCAAAAACTGGAATTCATTC
GTTCTGTGTGCGAAAGCAATGGTGCGGCAATTTTCTACATGCCGAGCATCGGTATGGATA
TGACCACCATCTTCGCGTCCAATACCCGTCTGGCGCCGATTCAGGCAATCGCCCTGGG
CCACCCGGCGACTACTCACTCCGACTTCATTGAATACGTTATCGTGGAAGACGACTACG
TCGGCTCTGAGGAATGCTTCTCTGAAACCCTGCTGCGTCTGCCGAAAGACGCTCTGCC
GTATGTTCCGTCCGCCCTGGCTCCGGAGAAAGTTGATTACCTGCTGCGTGAAAACCCTG
AAGTTGTCAACATCGGTATTGCCTCTACCACTATGAAGCTGAACCCGTACTTCCTGGAAG
CACTGAAGGCCATTCGTGACCGTGCGAAGGTGAAAGTGCACTTCCACTTCGCACTGGG
CCAGTCCAATGGTATCACTCACCCTTACGTTGAACGCTTTATCAAATCTTACCTGGGCGA
CAGCGCTACCGCGCACCCGCACTCTCCGTACCACCAGTACCTGCGTATTCTGCACAACT
GCGATATGATGGTAAACCCTTTTCCGTTTGGTAATACCAATGGTATTATTGACATGGTAAC
CCTGGGTCTGGTAGGTGTTTGCAAAACCGGTGCGGAAGTCCACGAACATATCGATGAAG
GCCTGTTCAAACGTCTGGGCCTGCCGGAATGGCTGATTGCAAACACCGTGGACGAATA
CGTGGAACGTGCAGTGCGCCTGGCCGAGAACCATCAGGAACGTCTGGAACTGCGTCG
TTACATTATTGAAAACAATGGCCTGAACACCCTGTTCACCGGCGACCCACGCCCGATGG
GTCAGGTGTTCCTGGAAAAACTGAACGCATTCCTGAAGGAAAACGGCGGCGACTACAA
GGACGATGACGACAAGGGATAAggtaccctcgaggataaggaggataagATGGAGAAAAAAATCTC
TGCGTGGAGCCATCCGCAGTTCGAAAAAGGATCCCAGAACCATGTTATTAGCCTGGCAA

GCGCAGCAGAACGTCGTGCACATATTGCAGATACCTTTGGTCGTCATGGTATTCCGTTTC
AGTTTTTTGATGCACTGATGCCGAGCGAACGTCTGGAACAGGCAATGGCAGAACTGGTT
CCGGGTCTGAGCGCACATCCGTATCTGAGCGGTGTTGAAAAAGCATGTTTTATGAGCCA
TGCAGTTCTGTGGAAACAGGCACTGGATGAAGGTCTGCCGTATATTACCGTTTTTGAAGA
TGATGTTCTGCTGGGTGAAGGTGCAGAAAAATTTCTGGCAGAAGATGCCTGGCTGCAAG
AACGTTTTGATCCGGATACCGCATTTATTGTTCGTCTGGAAACCATGTTTATGCATGTTCT
GACCAGCCCGAGCGGTGTGGCAGATTATTGTGGTCGTGCATTTCCGCTGCTGGAAAGC
GAACATTGGGGCACCGCAGGTTATATCATTAGCCGTAAAGCAATGCGCTTTTTTCTGGAT
CGTTTTGCAGCACTGCCTCCGGAAGGCCTGCATCCGGTTGATCTGATGATGTTTAGCGA
TTTTTTTGATCGTGAAGGTATGCCGGTTTGTCAGCTGAATCCGGCACTGTGTGCACAAG
AACTGCACTATGCAAAATTTCATGATCAGAATAGCGCACTGGGTAGCCTGATTGAACATG
ATCGTCTGCTGAATCGTAAACAGCAGCGTCGTGATAGTCCGGCAAATACCTTTAAACATC
GTCTGATTCGTGCCCTGACCAAAATTAGCCGTGAACGTGAAAAACGTCGTCAGCGTCGC
GAACAGTTTATTGTGCCGTTTCAGTAAaagcttggctgttttggcggatgagagaagattttcagcctgatacag
attaaatcagaac

DNA Sequence of CjCST-I.NmLgtB.ApNGT in pMAF10 Context:

ttgctatgccatagcatttttatccataagattagcggatcctacctgacgctttttatcgcaactctctactgtttctccatacccgtttttt
gggctagcaggaggaattccATGGAGAAAAAAATCTGGAGCCATCCGCAGTTCGAAAAAGGCGG
ATCCGGAGGCAGCCACATGACCCGTACCCGTATGGAAAATGAACTGATTGTGAGCAAAA
ACATGCAGAACATTATCATTGCAGGTAATGGTCCGAGCCTGAAAAACATTAACTATAAACG
TCTGCCTCGCGAGTATGATGTTTTTCGTTGTAACCAGTTCTATTTCGAGGATAAATACTAT
CTGGGCAAAAAAATCAAAGCCGTGTTTTTCAATCCGGGTGTTTTTCTGCAGCAGTATCAT
ACCGCAAAACAGCTGATTCTGAAAAACGAGTACGAGATCAAAAACATCTTTTGCAGCAC
CTTTAACCTGCCGTTTATTGAAAGCAACGATTTCCTGCACCAGTTTTACAACTTTTTTCCG
GATGCAAAACTGGGCTATGAAGTGATTGAAAACCTGAAAGAGTTCTACGCCTATATCAAA
TACAACGAGATCTATTTCAACAAACGCATTACCAGCGGTGTTTATATGTGTGCAATTGCCA
TTGCCCTGGGCTATAAAACCATTTATCTGTGCGGTATCGATTTCTATGAAGGCGACGTTAT
TTATCCGTTTGAAGCAATGAGCACCAACATTAAAACAATCTTCCCTGGCATCAAAGACTT
CAAACCGAGCAATTGTCACAGCAAAGAATATGATATTGAGGCCCTGAAACTGCTGAAAAG
CATCTATAAAGTGAACATCTATGCCCTGTGTGATGATAGCATTCTGGCAAATCATTTTCCG
CTGAGCATTAACATCAACAACAACTTTACCCTGGAAAACAAACACAACAACAGCATCAAT
GATATCCTGCTGACCGATAATACACCGGGTGTTAGCTTTTACAAAAATCAGCTGAAAGCC
GATAACAAAATTATGCTGAACTTTTATTAAggtaccctcgaggataaggaggataagATGGAGAAAAA
AATCTCTGCGTGGAGCCATCCGCAGTTCGAAAAAGGATCCCAGAACCATGTTATTAGCC
TGGCAAGCGCAGCAGAACGTCGTGCACATATTGCAGATACCTTTGGTCGTCATGGTATT
CCGTTTCAGTTTTTTGATGCACTGATGCCGAGCGAACGTCTGGAACAGGCAATGGCAGA
ACTGGTTCCGGGTCTGAGCGCACATCCGTATCTGAGCGGTGTTGAAAAAGCATGTTTTA
TGAGCCATGCAGTTCTGTGGAAACAGGCACTGGATGAAGGTCTGCCGTATATTACCGTT
TTTGAAGATGATGTTCTGCTGGGTGAAGGTGCAGAAAAATTTCTGGCAGAAGATGCCTG
GCTGCAAGAACGTTTTGATCCGGATACCGCATTTATTGTTCGTCTGGAAACCATGTTTAT
GCATGTTCTGACCAGCCCGAGCGGTGTGGCAGATTATTGTGGTCGTGCATTTCCGCTGC

TGGAAAGCGAACATTGGGGCACCGCAGGTTATATCATTAGCCGTAAAGCAATGCGCTTTT
TTCTGGATCGTTTTGCAGCACTGCCTCCGGAAGGCCTGCATCCGGTTGATCTGATGATG
TTTAGCGATTTTTTTGATCGTGAAGGTATGCCGGTTTGTCAGCTGAATCCGGCACTGTGT
GCACAAGAACTGCACTATGCAAAATTTCATGATCAGAATAGCGCACTGGGTAGCCTGATT
GAACATGATCGTCTGCTGAATCGTAAACAGCAGCGTCGTGATAGTCCGGCAAATACCTTT
AAACATCGTCTGATTCGTGCCCTGACCAAAATTAGCCGTGAACGTGAAAAACGTCGTCA
GCGTCGCGAACAGTTTATTGTGCCGTTTCAGTAAtgaaggtctagaggaggtaaaaATGGAAAAC
GAGAATAAACCGAACGTGGCAAATTTTGAAGCAGCAGTTGCAGCCAAAGATTATGAAAAA
GCATGTAGCGAGCTGCTGCTGATTCTGAGCCAGCTGGATAGCAATTTTGGTGGCATTCA
TGAAATCGAGTTCGAGTACCCAGCTCAGCTGCAGGATCTGGAGCAGGAAAAAATTGTGT
ACTTTTGCACCCGTATGGCGACTGCCATCACCACCCTGTTCTCTGACCCGGTTCTGGAA
ATCTCCGACCTGGGTGTGCAGCGTTTCCTGGTTTATCAGCGTTGGCTGGCGCTGATCTT
CGCTAGCAGCCCGTTCGTGAACGCAGACCACATCCTGCAGACTTACAACCGTGAACCG
AACCGCAAAAACTCCCTGGAAATTCACCTGGACTCTAGCAAGTCCTCTCTGATTAAATTT
TGCATCCTGTACCTGCCGGAATCTAATGTGAACCTGAATCTGGATGTGATGTGGAACATT
TCCCCGGAGCTGTGTGCCTCTCTGTGCTTTGCGCTGCAAAGCCCGCGTTTTGTTGGCA
CCAGCACCGCCTTTAACAAACGCGCGACCATTCTGCAGTGGTTCCCGCGTCATCTGGA
CCAGCTGAAAAACCTGAACAACATCCCGTCCGCTATCTCTCATGACGTGTATATGCACTG
TTCTTACGACACCAGCGTTAACAAGCACGATGTTAAGCGCGCGCTGAATCACGTGATTC
GTCGCCACATCGAATCCGAATACGGTTGGAAAGATCGTGATGTGGCTCACATCGGTTAT
CGCAACAACAAACCGGTTATGGTCGTTCTGCTGGAACATTTTCATAGCGCGCACTCTATC
TACCGTACTCACTCTACCAGCATGATCGCCGCGCGCGAACACTTCTATCTGATCGGCCT
GGGTTCCCCGAGCGTTGACCAGGCCGGTCAGGAGGTTTTCGATGAATTCCACCTGGTA
GCGGGTGACAACATGAAGCAAAAACTGGAATTCATTCGTTCTGTGTGCGAAAGCAATGG
TGCGGCAATTTTCTACATGCCGAGCATCGGTATGGATATGACCACCATCTTCGCGTCCAA
TACCCGTCTGGCGCCGATTCAGGCAATCGCCCTGGGCCACCCGGCGACTACTCACTCC
GACTTCATTGAATACGTTATCGTGGAAGACGACTACGTCGGCTCTGAGGAATGCTTCTCT
GAAACCCTGCTGCGTCTGCCGAAAGACGCTCTGCCGTATGTTCCGTCCGCCCTGGCTC
CGGAGAAAGTTGATTACCTGCTGCGTGAAAACCCTGAAGTTGTCAACATCGGTATTGCC
TCTACCACTATGAAGCTGAACCCGTACTTCCTGGAAGCACTGAAGGCCATTCGTGACCG
TGCGAAGGTGAAAGTGCACTTCCACTTCGCACTGGGCCAGTCCAATGGTATCACTCACC
CTTACGTTGAACGCTTTATCAAATCTTACCTGGGCGACAGCGCTACCGCGCACCCGCAC
TCTCCGTACCACCAGTACCTGCGTATTCTGCACAACTGCGATATGATGGTAAACCCTTTT
CCGTTTGGTAATACCAATGGTATTATTGACATGGTAACCCTGGGTCTGGTAGGTGTTTGC
AAAACCGGTGCGGAAGTCCACGAACATATCGATGAAGGCCTGTTCAAACGTCTGGGCC
TGCCGGAATGGCTGATTGCAAACACCGTGGACGAATACGTGGAACGTGCAGTGCGCCT
GGCCGAGAACCATCAGGAACGTCTGGAACTGCGTCGTTACATTATTGAAAACAATGGCC
TGAACACCCTGTTCACCGGCGACCCACGCCCGATGGGTCAGGTGTTCCTGGAAAAACT
GAACGCATTCCTGAAGGAAAACggcggcGACTACAAGGACGATGACGACAAGGGATAAaag
cttggctgttttggcggatgagagaagattttcagcctgatacagattaaatcagaac

DNA Sequence of PdST6.NmLgtB.ApNGT in pMAF10 Context:
ttgctatgccatagcatttttatccataagattagcggatcctacctgacgctttttatcgcaactctctactgtttctccatacccgttttttt

gggctagcaggaggaattccATGGAGAAAAAAATCTGGAGCCATCCGCAGTTCGAAAAAGGCGG
ATCCGGACTGGTTCCGCGTGGTAGCCACATGTGTAATAGCGATAACACCAGCCTGAAAG
AAACCGTTAGCAGCAATAGCGCAGATGTTGTTGAAACCGAAACCTATCAGCTGACCCCG
ATTGATGCACCGAGCAGCTTTCTGAGCCATAGCTGGGAACAGACCTGTGGCACCCCGAT
TCTGAATGAAAGCGATAAACAGGCAATCAGCTTTGATTTTGTTGCACCGGAACTGAAACA
GGATGAGAAATATTGCTTTACCTTCAAAGGCATTACCGGTGATCATCGTTATATTACCAATA
CCACCCTGACCGTTGTGGCACCGACCCTGGAAGTTTATATTGATCATGCAAGCCTGCCG
AGCCTGCAGCAGCTGATTCATATTATTCAGGCCAAAGATGAATATCCGAGCAATCAGCGT
TTTGTTAGCTGGAAACGTGTTACCGTTGATGCAGATAATGCCAACAAACTGAACATTCATA
CCTATCCGCTGAAAGGCAATAATACCAGTCCGGAAATGGTTGCAGCAATTGATGAATATG
CACAGAGCAAAAATCGCCTGAACATCGAGTTTTATACCAATACAGCCCACGTGTTTAATAA
CCTGCCTCCGATTATTCAGCCGCTGTATAATAACGAGAAAGTGAAAATTAGCCACATCAG
CCTGTATGATGACGGTAGCAGCGAATATGTTAGCCTGTATCAGTGGAAAGATACCCCGAA
CAAAATTGAAACACTGGAAGGTGAAGTTAGCCTGCTGGCAAATTATCTGGCAGGCACCT
CACCGGATGCTCCGAAAGGTATGGGTAATCGTTATAATTGGCACAAACTGTATGACACCG
ACTATTACTTTCTGCGCGAAGATTATCTGGATGTTGAAGCAAATCTGCATGATCTGCGTGA
TTACCTGGGTAGCAGTGCAAAACAAATGCCGTGGGATGAATTTGCAAAACTGAGCGATA
GCCAGCAGACCCTGTTTCTGGATATTGTTGGTTTTGATAAAGAACAGCTGCAGCAACAGT
ATAGCCAGAGTCCGCTGCCGAATTTTATCTTTACCGGCACCACCACCTGGGCAGGCGGT
GAAACCAAAGAATATTATGCCCAGCAGCAGGTTAACGTGATTAACAATGCAATTAATGAAA
CCAGCCCGTACTATCTGGGTAAAGATTATGACCTGTTTTTCAAAGGTCATCCTGCCGGTG
GTGTGATTAATGATATTATTCTGGGTAGCTTCCCGGATATGATTAACATTCCGGCAAAAATT
AGCTTCGAGGTTCTGATGATGACCGATATGCTGCCGGATACCGTTGCAGGTATTGCAAG
CAGTCTGTATTTCACAATTCCGGCAGATAAAGTGAACTTCATTGTTTTTACCAGCAGCGAT
ACCATTACCGATCGTGAAGAAGCACTGAAAAGTCCGCTGGTTCAGGTTATGCTGACCCT
GGGTATTGTTAAAGAAAAAGATGTTCTGTTTTGGGCATAAggtaccctcgaggataaggaggataag
ATGGAGAAAAAAATCTCTGCGTGGAGCCATCCGCAGTTCGAAAAAGGATCCCAGAACCA
TGTTATTAGCCTGGCAAGCGCAGCAGAACGTCGTGCACATATTGCAGATACCTTTGGTC
GTCATGGTATTCCGTTTCAGTTTTTTGATGCACTGATGCCGAGCGAACGTCTGGAACAG
GCAATGGCAGAACTGGTTCCGGGTCTGAGCGCACATCCGTATCTGAGCGGTGTTGAAA
AAGCATGTTTTATGAGCCATGCAGTTCTGTGGAAACAGGCACTGGATGAAGGTCTGCCG
TATATTACCGTTTTTGAAGATGATGTTCTGCTGGGTGAAGGTGCAGAAAAATTTCTGGCA
GAAGATGCCTGGCTGCAAGAACGTTTTGATCCGGATACCGCATTTATTGTTCGTCTGGAA
ACCATGTTTATGCATGTTCTGACCAGCCCGAGCGGTGTGGCAGATTATTGTGGTCGTGC
ATTTCCGCTGCTGGAAAGCGAACATTGGGGCACCGCAGGTTATATCATTAGCCGTAAAG
CAATGCGCTTTTTTCTGGATCGTTTTGCAGCACTGCCTCCGGAAGGCCTGCATCCGGTT
GATCTGATGATGTTTAGCGATTTTTTTGATCGTGAAGGTATGCCGGTTTGTCAGCTGAATC
CGGCACTGTGTGCACAAGAACTGCACTATGCAAAATTTCATGATCAGAATAGCGCACTG
GGTAGCCTGATTGAACATGATCGTCTGCTGAATCGTAAACAGCAGCGTCGTGATAGTCC
GGCAAATACCTTTAAACATCGTCTGATTCGTGCCCTGACCAAAATTAGCCGTGAACGTGA
AAAACGTCGTCAGCGTCGCGAACAGTTTATTGTGCCGTTTCAGTAAtgaaggtctagaggaggta
aaaATGGAAAACGAGAATAAACCGAACGTGGCAAATTTTGAAGCAGCAGTTGCAGCCAAA
GATTATGAAAAAGCATGTAGCGAGCTGCTGCTGATTCTGAGCCAGCTGGATAGCAATTTT

GGTGGCATTCATGAAATCGAGTTCGAGTACCCAGCTCAGCTGCAGGATCTGGAGCAGG
AAAAAATTGTGTACTTTTGCACCCGTATGGCGACTGCCATCACCACCCTGTTCTCTGACC
CGGTTCTGGAAATCTCCGACCTGGGTGTGCAGCGTTTCCTGGTTTATCAGCGTTGGCTG
GCGCTGATCTTCGCTAGCAGCCCGTTCGTGAACGCAGACCACATCCTGCAGACTTACAA
CCGTGAACCGAACCGCAAAAACTCCCTGGAAATTCACCTGGACTCTAGCAAGTCCTCTC
TGATTAAATTTTGCATCCTGTACCTGCCGGAATCTAATGTGAACCTGAATCTGGATGTGAT
GTGGAACATTTCCCCGGAGCTGTGTGCCTCTCTGTGCTTTGCGCTGCAAAGCCCGCGT
TTTGTTGGCACCAGCACCGCCTTTAACAAACGCGCGACCATTCTGCAGTGGTTCCCGC
GTCATCTGGACCAGCTGAAAAACCTGAACAACATCCCGTCCGCTATCTCTCATGACGTG
TATATGCACTGTTCTTACGACACCAGCGTTAACAAGCACGATGTTAAGCGCGCGCTGAAT
CACGTGATTCGTCGCCACATCGAATCCGAATACGGTTGGAAAGATCGTGATGTGGCTCA
CATCGGTTATCGCAACAACAAACCGGTTATGGTCGTTCTGCTGGAACATTTTCATAGCGC
GCACTCTATCTACCGTACTCACTCTACCAGCATGATCGCCGCGCGCGAACACTTCTATCT
GATCGGCCTGGGTTCCCCGAGCGTTGACCAGGCCGGTCAGGAGGTTTTCGATGAATTC
CACCTGGTAGCGGGTGACAACATGAAGCAAAAACTGGAATTCATTCGTTCTGTGTGCGA
AAGCAATGGTGCGGCAATTTTCTACATGCCGAGCATCGGTATGGATATGACCACCATCTT
CGCGTCCAATACCCGTCTGGCGCCGATTCAGGCAATCGCCCTGGGCCACCCGGCGACT
ACTCACTCCGACTTCATTGAATACGTTATCGTGGAAGACGACTACGTCGGCTCTGAGGA
ATGCTTCTCTGAAACCCTGCTGCGTCTGCCGAAAGACGCTCTGCCGTATGTTCCGTCCG
CCCTGGCTCCGGAGAAAGTTGATTACCTGCTGCGTGAAAACCCTGAAGTTGTCAACATC
GGTATTGCCTCTACCACTATGAAGCTGAACCCGTACTTCCTGGAAGCACTGAAGGCCAT
TCGTGACCGTGCGAAGGTGAAAGTGCACTTCCACTTCGCACTGGGCCAGTCCAATGGT
ATCACTCACCCTTACGTTGAACGCTTTATCAAATCTTACCTGGGCGACAGCGCTACCGCG
CACCCGCACTCTCCGTACCACCAGTACCTGCGTATTCTGCACAACTGCGATATGATGGTA
AACCCTTTTCCGTTTGGTAATACCAATGGTATTATTGACATGGTAACCCTGGGTCTGGTAG
GTGTTTGCAAAACCGGTGCGGAAGTCCACGAACATATCGATGAAGGCCTGTTCAAACGT
CTGGGCCTGCCGGAATGGCTGATTGCAAACACCGTGGACGAATACGTGGAACGTGCAG
TGCGCCTGGCCGAGAACCATCAGGAACGTCTGGAACTGCGTCGTTACATTATTGAAAAC
AATGGCCTGAACACCCTGTTCACCGGCGACCCACGCCCGATGGGTCAGGTGTTCCTGG
AAAAACTGAACGCATTCCTGAAGGAAAACGGCGGCGACTACAAGGACGATGACGACAA
GGGATAAaagcttggctgttttggcggatgagagaagattttcagcctgatacagattaaatcagaac

DNA sequence of pCon.ConNeuA:

gatgcatttgacggctagctcagtcctaggtacagtgctagcgaattcgagctcccgggaggaggaacgATGAGAACAA
AAATTATTGCGATAATTCCAGCCCGTAGTGGATCTAAAGGGTTGAGAAATAAAAATGCTTT
GATGCTGATAGATAAACCTCTTCTTGCTTATACAATTGAAGCTGCCTTGCAGTCAGAAATG
TTTGAGAAAGTAATTGTGACAACTGACTCCGAACAGTATGGAGCAATAGCAGAGTCATAT
GGTGCTGATTTTTTGCTGAGACCGGAAGAACTAGCAACTGATAAAGCATCATCATTTGAA
TTTATAAAACATGCGTTAAGTATATATACTGATTATGAGAACTTTGCTTTATTACAACCAACT
TCACCCTTTAGAGATTCGACCCATATTATTGAGGCTGTAAAGTTATATCAAACTTTAGAAAA
ATACCAATGTGTTGTTTCTGTTACTAGAAGCAATAAGCCATCACAAATAATTAGACCATTAG
ATGATTACTCGACACTGTCTTTTTTTGACCTTGATTATAGTAAATATAATCGAAACTCAATAG
TAGAATATCATCCGAATGGAGCTATATTTATAGCTAATAAGCAGCATTATCTTCATACAAAGC

ATTTTTTTGGTCGCTATTCACTAGCTTATATTATGGATAAGGAAAGCTCTTTAGATATAGATG
ATAGAATGGATTTCGAACTTGCAATTACCATTCAGCAAAAAAAAAATAGACAAAAAATACTT
TATCAAAACATACATAATAGAATCAATGAGAAACGAAATGAATTTGATAGTGTAAGTGATAT
AACTTTAATTGGACACTCGCTGTTTGATTATTGGGACGTAAAAAAAATAAATGATATAGAAG
TTAATAACTTAGGTATCGCTGGTATAAACTCGAAGGAGTACTATGAATATATTATTGAGAAA
GAGCGGATTGTTAATTTCGGAGAGTTTGTTTTCATCTTTTTTGGAACTAATGATATAGTTGT
TAGTGATTGGAAAAAAGAAGACACATTGTGGTATTTGAAGAAAACATGCCAGTATATAAAG
AAGAAAAATGCTGCATCAAAAATTTATTTATTGTCGGTTCCTCCTGTTTTTGGGCGTATTG
ATCGAGATAATAGAATAATTAATGATTTAAATTCTTATCTTCGAGAGAATGTAGATTTTGCGA
AGTTTATTAGCTTGGATCACGTTTTAAAAGACTCTTATGGCAATCTAAATAAAATGTATACTT
ATGATGGCTTACATTTTAATAGTAATGGGTATACAGTATTAGAAAACGAAATAGCGGAGATT
GTTAAATGActtaattaatctagagtcgacctgcaggcatgcaagcttggctgttttggcggatgagagaagattttcagcctg
atacagattaaatcagaacgcagaagcggtctgataaaacagaatttgcctggcggcagtagcgcggtggtcccacctgaccc
catgccgaactcagaagtgaaacgccgtagcgccgatggtagtgtggggtctccccatgcgagagtagggaactgccaggca
tcaaataaaacgaaaggctcagtcgaaagactgggcctttcgttttatctgttgtttgtcggtgaacgctctcctgagtaggacaaat

Sequence of pBR322.Fc-6 in Context:

gaaatgagctgttgacaattaatcatccggctcgtataatgtgtggaattgtgagcggataacaatttcacacaggaaacagacc
ATGGAACCGAAAAGCTGTGATAAAACCCATACCTGTCCGCCTTGTCCGGCACCGGAACT
GCTGGGTGGTCCGAGCGTTTTTCTGTTTCCGCCTAAACCGAAAGATACCCTGATGATTA
GCCGTACACCGGAAGTTACCTGTGTTGTTGTTGATGTTAGCCATGAAGATCCGGAAGTG
AAATTTAACTGGTATGTTGATGGTGTGGAAGTGCATAATGCAAAAACCAAACCGCGTGAA
GAAGCGACTACC**GGAGGTAACTGGACAACA**GCGGGAGGACGTGTTGTTAGCGTTCTG
ACCGTTCTGCATCAGGATTGGCTGAATGGTAAAGAATACAAATGCAAAGTGAGCAACAAA
GCACTGCCTGCACCGATTGAAAAAACCATTAGCAAAGCAAAAGGTCAGCCTCGTGAACC
GCAGGTTTATACCCTGCCTCCGAGCCGTGATGAACTGACCAAAAATCAGGTTAGCCTGA
CCTGTCTGGTGAAAGGTTTTTATCCGAGCGATATTGCAGTTGAATGGGAAAGCAATGGTC
AGCCGGAAAATAACTATAAAACCACCCCTCCGGTTCTGGATAGTGATGGTAGCTTTTTTC
TGTATAGCAAACTGACCGTTGATAAAAGCCGTTGGCAGCAGGGTAATGTTTTTAGCTGTA
GCGTTATGCATGAAGCCCTGCATAATCATTATACCCAGAAAAGCCTGAGCCTGAGTCCGG
GTAAAGGTAGCCATCATCATCACCATCATTAAaagcttggctgttttggcggatgagagaagattttcagcctg
atacagattaaatcagaacgcagaagcggtctgataaaacagaatttgcctggcggcagtagcgcggtggtcccacctgaccc
catgccgaactcagaagtgaaacgccgtagcgccgatggtagtgtggggtctccccatgcgagagtagggaactgccaggca
tcaaataaaacgaaaggctcagtcgaaagactgggcctttcgttttatctgttgtttgtcggtgaacgctctcctgagtaggacaaat

Sequence of pBR322.Im7-6 in Context:

gaaatgagctgttgacaattaatcatccggctcgtataatgtgtggaattgtgagcggataacaatttcacacaggaaacagacc
ATGGAACTGGAAAATAGTATTAGTGATTACACAGAGGCTGAGTTTGTTCAACTTCTTAAGG
AAATTGAAAAAGAGGCGACTACC**GGAGGTAACTGGACAACA**GCGGGAGGAGATGTGTT
AGATGTGTTACTCGAACACTTTGTAAAAATTACTGAGCATCCAGATGGAACGGATCTGAT
CTATTATCCTAGTGATAATAGAGACGATAGCCCCGAAGGGATTGTCAAGGAAATTAAAGAA
TGGCGAGCTGCTAACGGTAAGCCAGGATTTAAACAGGGCGGATCCCATCACCATCATCA
CCATTAAaagcttggctgttttggcggatgagagaagattttcagcctgatacagattaaatcagaacgcagaagcggtctga

taaaacagaatttgcctggcggcagtagcgcggtggtcccacctgaccccatgccgaactcagaagtgaaacgccgtagcgc
cgatggtagtgtggggtctccccatgcgagagtagggaactgccaggcatcaaataaaacgaaaggctcagtcgaaagactg
ggcctttcgttttatctgttgtttgtcggtgaacgctctcctgagtaggacaaat

Supplementary Information References

1. Martin, R.W. et al. Cell-free protein synthesis from genomically recoded bacteria enables multisite incorporation of noncanonical amino acids. *Nature Communications* **9**, 1203 (2018).
2. Bundy, B.C. & Swartz, J.R. Site-Specific Incorporation of p-Propargyloxyphenylalanine in a Cell-Free Environment for Direct Protein−Protein Click Conjugation. *Bioconjugate chemistry* **21**, 255-263 (2010).
3. Kightlinger, W. et al. Design of glycosylation sites by rapid synthesis and analysis of glycosyltransferases. *Nature Chemical Biology* **14**, 627-635 (2018).
4. Ollis, A.A., Zhang, S., Fisher, A.C. & DeLisa, M.P. Engineered oligosaccharyltransferases with greatly relaxed acceptor-site specificity. *Nature Chemical Biology* **10**, 816-822 (2014).
5. Glasscock, C.J. et al. A flow cytometric approach to engineering Escherichia coli for improved eukaryotic protein glycosylation. *Metabolic Engineering* **47**, 488-495 (2018).
6. Valentine, Jenny L. et al. Immunization with Outer Membrane Vesicles Displaying Designer Glycotopes Yields Class-Switched, Glycan-Specific Antibodies. *Cell Chemical Biology* **23**, 655-665 (2016).
7. Naegeli, A. et al. Substrate Specificity of Cytoplasmic N-Glycosyltransferase. *Journal of Biological Chemistry* **289**, 24521-24532 (2014).
8. Schwarz, F., Fan, Y.-Y., Schubert, M. & Aebi, M. Cytoplasmic N-Glycosyltransferase of Actinobacillus pleuropneumoniae Is an Inverting Enzyme and Recognizes the NX(S/T) Consensus Sequence. *Journal of Biological Chemistry* **286**, 35267-35274 (2011).
9. Park, J.E., Lee, K.Y., Do, S.I. & Lee, S.S. Expression and characterization of beta-1,4-galactosyltransferase from Neisseria meningitidis and Neisseria gonorrhoeae. *Journal of biochemistry and molecular biology* **35**, 330-336 (2002).
10. Peng, W. et al. Helicobacter pylori β1,3-N-acetylglucosaminyltransferase for versatile synthesis of type 1 and type 2 poly-LacNAcs on N-linked, O-linked and I-antigen glycans. *Glycobiology* **22**, 1453-1464 (2012).
11. Ramakrishnan, B. & Qasba, P.K. Crystal structure of lactose synthase reveals a large conformational change in its catalytic component, the beta1,4-galactosyltransferase-I. *Journal of Molecular Biology* **310**, 205-218 (2001).
12. Aanensen, D.M., Mavroidi, A., Bentley, S.D., Reeves, P.R. & Spratt, B.G. Predicted Functions and Linkage Specificities of the Products of the Streptococcus pneumoniae Capsular Biosynthetic Loci. *Journal of bacteriology* **189**, 7856-7876 (2007).
13. Ban, L. et al. Discovery of glycosyltransferases using carbohydrate arrays and mass spectrometry. *Nature Chemical Biology* **8**, 769-773 (2012).
14. Blixt, O., van Die, I., Norberg, T. & van den Eijnden, D.H. High-level expression of the Neisseria meningitidis lgtA gene in Escherichia coli and characterization of the encoded N-acetylglucosaminyltransferase as a useful catalyst in the synthesis of GlcNAcβ1→3Gal and GalNAcβ1→3Gal linkages. *Glycobiology* **9**, 1061-1071 (1999).
15. Higuchi, Y. et al. A rationally engineered yeast pyruvyltransferase Pvg1p introduces sialylation-like properties in neo-human-type complex oligosaccharide. *Scientific*

reports **6**, 26349 (2016).
16. Sun, S., Scheffler, N.K., Gibson, B.W., Wang, J. & Munson Jr., R.S. Identification and Characterization of the N-Acetylglucosamine Glycosyltransferase Gene of Haemophilus ducreyi. *Infection and immunity* **70**, 5887-5892 (2002).
17. Wang, G., Ge, Z., Rasko, D.A. & Taylor, D.E. Lewis antigens in Helicobacter pylori: biosynthesis and phase variation. *Molecular Microbiology* **36**, 1187-1196 (2000).
18. Persson, K. et al. Crystal structure of the retaining galactosyltransferase LgtC from Neisseria meningitidis in complex with donor and acceptor sugar analogs. *Nature Structural Biology* **8**, 166 (2001).
19. Fang, J. et al. Highly Efficient Chemoenzymatic Synthesis of α-Galactosyl Epitopes with a Recombinant α(1→3)-Galactosyltransferase. *Journal of the American Chemical Society* **120**, 6635-6638 (1998).
20. Hidari, K.I. et al. Purification and characterization of a soluble recombinant human ST6Gal I functionally expressed in Escherichia coli. *Glycoconjugate Journal* **22**, 1-11 (2005).
21. Yamamoto, T. Marine Bacterial Sialyltransferases. *Marine Drugs* **8**, 2781 (2010).
22. Chiu, C.P.C. et al. Structural Analysis of the α-2,3-Sialyltransferase Cst-I from Campylobacter jejuni in Apo and Substrate-Analogue Bound Forms. *Biochemistry* **46**, 7196-7204 (2007).
23. Keys, T.G. et al. A biosynthetic route for polysialylating proteins in Escherichia coli. *Metabolic Engineering* **44**, 293-301 (2017).
24. Kim, D.M. & Swartz, J.R. Efficient production of a bioactive, multiple disulfide-bonded protein using modified extracts of Escherichia coli. *Biotechnology and bioengineering* **85**, 122-129 (2004).
