# Supplementary material for: A cell-free biosynthesis platform for modular construction of protein glycosylation pathways
Source: Nat Commun. 2019 Nov 27;10:5404. doi: 10.1038/s41467-019-12024-9 (PMC6881289; doi:10.1038/s41467-019-12024-9)
Supplement: Supplementary file 2 — Reporting Summary [file 41467_2019_12024_MOESM2_ESM.pdf]

nature research

Corresponding author(s): Michael C. Jewett

Last updated by author(s): 2019/07/22

Reporting Summary

Nature Research wishes to improve the reproducibility of the work that we publish. This form provides structure for consistency and transparency in reporting. For further information on Nature Research policies, see Authors & Referees and the Editorial Policy Checklist.

Statistics

For all statistical analyses, confirm that the following items are present in the figure legend, table legend, main text, or Methods section.

n/a	Confirmed	
☐	☒	The exact sample size (*n*) for each experimental group/condition, given as a discrete number and unit of measurement
☐	☒	A statement on whether measurements were taken from distinct samples or whether the same sample was measured repeatedly
☒	☐	The statistical test(s) used AND whether they are one- or two-sided<br>*Only common tests should be described solely by name; describe more complex techniques in the Methods section.*
☒	☐	A description of all covariates tested
☐	☒	A description of any assumptions or corrections, such as tests of normality and adjustment for multiple comparisons
☐	☒	A full description of the statistical parameters including central tendency (e.g. means) or other basic estimates (e.g. regression coefficient) AND variation (e.g. standard deviation) or associated estimates of uncertainty (e.g. confidence intervals)
☒	☐	For null hypothesis testing, the test statistic (e.g. *F*, *t*, *r*) with confidence intervals, effect sizes, degrees of freedom and *P* value noted<br>*Give P values as exact values whenever suitable.*
☒	☐	For Bayesian analysis, information on the choice of priors and Markov chain Monte Carlo settings
☒	☐	For hierarchical and complex designs, identification of the appropriate level for tests and full reporting of outcomes
☒	☐	Estimates of effect sizes (e.g. Cohen's *d*, Pearson's *r*), indicating how they were calculated

Our web collection on statistics for biologists contains articles on many of the points above.

Software and code

Policy information about availability of computer code

Data collection	All data was collected using stated instruments and associated commercially-available software. LC-MS(/MS) spectra were collected using Bruker Compass HyStar v4.1 (Bruker Daltonics, Inc.). SDS-PAGE and autoradiogram gel images were acquired using Image Lab software version 6.0.0 and Typhoon FLA 7000 Version 1.2 Build 1.2.1.93, respectively. No custom algorithms or softwares were used.
Data analysis	All data was analyzed using stated instruments and associated commercially-available software. LC-MS(/MS) spectra were analyzed using Bruker Compass Data Analysis v4.1 (Bruker Daltonics, Inc.). The resulting protein or peptide mass spectra were plotted using Microsoft Office Excel 365 (Microsoft). No custom algorithms or softwares were used.

For manuscripts utilizing custom algorithms or software that are central to the research but not yet described in published literature, software must be made available to editors/reviewers. We strongly encourage code deposition in a community repository (e.g. GitHub). See the Nature Research guidelines for submitting code & software for further information.

Data

Policy information about availability of data

All manuscripts must include a data availability statement. This statement should provide the following information, where applicable:

- Accession codes, unique identifiers, or web links for publicly available datasets
- A list of figures that have associated raw data
- A description of any restrictions on data availability

All data generated or analyzed during this study are included in this article and its supplementary materials or are available from the corresponding authors upon reasonable request. The source data underlying the averages reported in Supplementary Table 2 are provided as a Source Data file.

Field-specific reporting

Please select the one below that is the best fit for your research. If you are not sure, read the appropriate sections before making your selection.

☒ Life sciences ☐ Behavioural & social sciences ☐ Ecological, evolutionary & environmental sciences

For a reference copy of the document with all sections, see nature.com/documents/nr-reporting-summary-flat.pdf

Life sciences study design

All studies must disclose on these points even when the disclosure is negative.

Sample size	Exact sample numbers are provided in figure legends, including for representative spectra. Deconvoluted protein for identification of biosynthetic pathway products used at least n=2 in vitro glycosylation reactions. Qualitative determination of exoglycosidase digestion patterns used n=1 in vitro glycosylation reactions. Qualitative determination of peptide fragmentation patterns are representative of many MS/MS acquisitions from n=1 in vitro glycosylation reaction. Due to the high specificity of this technique, the analysis of n=1 samples for qualitative identification is regular practice in LC-MS studies. As is regular practice in cell-free protein synthesis (CFPS) literature, the mean and standard errors of CFPS yields of NGTs were determined by radioactive quantification from at least n=3 independent reactions.
Data exclusions	No data was excluded.
Replication	All attempts at replication were successful. The design of the study also demonstrated reproducibility of biosynthetic pathways because the products of many in vitro glycosylation reactions were generated again as substrates for more complex pathways. Selected pathways were also shown to be functional in different contexts, such as one-pot cell-free protein synthesis driven glycoprotein synthesis (CFPS-GpS) reactions and engineered bacterial strains.
Randomization	We performed systematic synthesis and evaluation of biosynthetic pathways in which samples underwent controlled enzymatic glycosylation and could be analyzed to completion by mass spectrometry. Therefore, no randomization was used. No animals or human participants were used in this study.
Blinding	Blinding was is not relevant to this study because all protein samples were glycosylated in vitro or in lab strains of E. coli bacteria and were fully characterized by mass spectrometry. No animals or human participants were used in this study.

Reporting for specific materials, systems and methods

We require information from authors about some types of materials, experimental systems and methods used in many studies. Here, indicate whether each material, system or method listed is relevant to your study. If you are not sure if a list item applies to your research, read the appropriate section before selecting a response.

Materials & experimental systems

n/a	Involved in the study	
☒	☐	Antibodies
☒	☐	Eukaryotic cell lines
☒	☐	Palaeontology
☒	☐	Animals and other organisms
☒	☐	Human research participants
☒	☐	Clinical data

Methods

n/a	Involved in the study	
☒	☐	ChIP-seq
☒	☐	Flow cytometry
☒	☐	MRI-based neuroimaging
